# Rationale and Roadmap for Developing Panels of Hotspot Cancer Driver Gene Mutations as Biomarkers of Cancer Risk

**DOI:** 10.1002/em.22326

**Published:** 2019-10-06

**Authors:** Kelly L. Harris, Meagan B. Myers, Karen L. McKim, Rosalie K. Elespuru, Barbara L. Parsons

**Affiliations:** ^1^ Division of Genetic and Molecular Toxicology National Center for Toxicological Research, US Food and Drug Administration Jefferson Arkansas; ^2^ Division of Biology, Chemistry and Materials Science CDRH/OSEL, US Food and Drug Administration Silver Spring Maryland

**Keywords:** mutation, biomarker, carcinogenesis, cell signaling, cell communication, cancer risk assessment

## Abstract

Cancer driver mutations (CDMs) are necessary and causal for carcinogenesis and have advantages as reporters of carcinogenic risk. However, little progress has been made toward developing measurements of CDMs as biomarkers for use in cancer risk assessment. Impediments for using a CDM‐based metric to inform cancer risk include the complexity and stochastic nature of carcinogenesis, technical difficulty in quantifying low‐frequency CDMs, and lack of established relationships between cancer driver mutant fractions and tumor incidence. Through literature review and database analyses, this review identifies the most promising targets to investigate as biomarkers of cancer risk. Mutational hotspots were discerned within the 20 most mutated genes across the 10 deadliest cancers. Forty genes were identified that encompass 108 mutational hotspot codons overrepresented in the COSMIC database; 424 different mutations within these hotspot codons account for approximately 63,000 tumors and their prevalence across tumor types is described. The review summarizes literature on the prevalence of CDMs in normal tissues and suggests such mutations are direct and indirect substrates for chemical carcinogenesis, which occurs in a spatially stochastic manner. Evidence that hotspot CDMs (hCDMs) frequently occur as tumor subpopulations is presented, indicating COSMIC data may underestimate mutation prevalence. Analyses of online databases show that genes containing hCDMs are enriched in functions related to intercellular communication. In its totality, the review provides a roadmap for the development of tissue‐specific, CDM‐based biomarkers of carcinogenic potential, comprised of batteries of hCDMs and can be measured by error‐correct next‐generation sequencing. Environ. Mol. Mutagen. 61:152–175, 2020. Published 2019. This article is a U.S. Government work and is in the public domain in the USA. *Environmental and Molecular Mutagenesis* published by Wiley Periodicals, Inc. on behalf of Environmental Mutagen Society.

**Abbreviations**
ACB‐PCR: allele‐specific competitive blocker–polymerase chain reaction
AML: acute myeloid leukemia
AOP: adverse outcome pathway
CD: cancer driver
CDMs: cancer driver mutations
ctDNA: circulating tumor DNA; EC‐NGS, error‐corrected next generation sequencing
hCDMs: hotspot cancer driver mutations
ICH: International Conference on Harmonization
MoA: mode‐of‐action
MF: mutant fraction
NGS: next‐generation sequencing
NMU: *N*‐nitroso‐*N*‐methylurea
TSG: tumor suppressor gene
UFs: uncertainty factors.

## INTRODUCTION

Cancer driver mutations (CDMs) have potential as biomarkers for use in carcinogenicity testing and assessing potential cancer risks associated with exogenous exposures, whether therapeutic, occupational, or environmental. While several valuable analyses identifying cancer driver (CD) genes have been published (Kandoth et al. 2013; Tokheim et al. 2016; Bailey et al. 2018; Iranzo et al. 2018), this review is focused on identifying the most useful hotspot cancer driver mutations (hCDMs, the most prevalent mutations detected in cancers) that could be employed as cancer biomarkers measured by error‐corrected next‐generation sequencing (EC‐NGS). To achieve this, the review includes summaries of available literature and reports the results of novel COSMIC database and text mining analyses.

The rationale for developing hCDMs as biomarkers of cancer risk is presented in sections describing carcinogenicity testing, unmet needs related to cancer risk assessment, and the theoretical advantages of CDMs as biomarkers of cancer risk. Studies that inform how levels of CDMs change through the stages of carcinogenesis are described in sections on the prevalence of hCDMs in normal tissues and the prevalence of hCDMs in tumors (including as mutant subpopulations). A major focus of the review is on identifying the most promising hCDMs to investigate, along with the properties they are expected to possess. For the purpose of this review, hCDMs are defined as base substitution mutations (missense and nonsense), and frame shifts occurring at codons accounting for at least 1% of each tumor type characterized with respect to a given gene. The choice was made to investigate mutations that occur in tumors with a prevalence of at least 1% in at least one of the 10 deadliest cancers based on two offsetting ideas. Mutations that appear in less than 1% of tumors are expected to contribute little to the usefulness of a composite biomarker because they would be enriched in relatively few tumors; however, mutations detected by standard DNA sequencing have increased prevalence when tumors are assessed using more sensitive methods than standard DNA sequencing (Parsons and Myers 2013b; Myers et al. 2015).

Finally, the review describes a field cancerization‐based view of carcinogenesis, in which multiclonal tumor initiation and interclonal cooperation are highlighted as important features. It utilized text mining of available literature to explore which CD gene products have functions related to cell communication and describes investigational paradigms that could be used to establish the utility of panels of hCDMs as reporters of cancer risk.

### Current Approaches for Carcinogenicity Testing and Evaluation

Regulatory agencies [US Food and Drug Administration (FDA), European Medicines Agency, US Environmental Protection Agency, and European Chemical Agency] have different laws and guidelines (e.g., ICH S1B or OECD test guideline 451) that identify what triggers a requirement for carcinogenicity testing (Corvi et al. 2017; OECD 2018; https://www.ema.europa.eu/en/ich-s1b-carcinogenicity-testing-carcinogenicity-pharmaceuticals). Regulatory requirement for carcinogenicity testing may be related to duration of drug exposure or tonnage of chemical use. While the use of CDMs as biomarkers has the theoretical potential to improve carcinogenicity assessment in relation to environmental, occupational, dietary, and therapeutic chemical exposures, this review will focus primarily on the carcinogenicity testing of products regulated by the FDA. Most major categories of products reviewed at the FDA undergo assessment for cancer hazard identification and risk analysis. This includes an assessment of qualitative findings from genotoxicity tests, relevant data assessments, and in some instances, 2‐year rodent bioassays. In the attempt to minimize the use of animals, more recently alternative approaches have included the assessment of data from chemical characterizations, quantitative structure–activity relationships, and toxicology risk assessments. Although the focus is primarily on hazard identification, or classification of agents as genotoxic or nongenotoxic, FDA regulatory review also addresses identification of potential nongenotoxic carcinogens and consideration of negative factors for carcinogenesis, such as the absence of hormonal action, rodent neoplasia, and certain histopathological risk factors.

A diversity of laws, regulations, standards, and products affect the approach and use of cancer risk assessments in different FDA centers (see Parsons 2018a for a history of regulatory assessments in the Center for Drugs). However, there are commonalities considered across FDA centers and regulatory agencies, including human exposure, genotoxicity assessment, available acceptable exposure limits, and the use or indication of the product. All regulatory entities generally try to limit the use of animals, including animal use in long‐term carcinogenicity bioassays. In some regulatory contexts, prioritization of risk of many agents, for example, environmental chemicals, may be important. In other cases, single agents under regulatory consideration are assessed on a case by case basis.

One approach to cancer risk analysis is to stratify long‐term bioassay testing dependent on risk. Thus, genotoxic drugs may be considered as a cancer risk *a priori* without additional testing, non‐genotoxic agents are not tested, and testing is restricted to cases where a rodent bioassay would be informative. However, there is a great need for new approaches and new tools for assessing cancer risk, particularly within the framework of alternatives to long‐term rodent bioassays.

Several relatively new initiatives are addressing how to improve cancer risk assessment. These include describing adverse outcome pathways (AOPs) leading to cancer, analyses of how chemical mode‐of‐action (MoA) translates into carcinogenesis and considering real‐world exposures to complex low‐dose mixtures. Key events in carcinogenesis can be identified based on Bradford Hill considerations and used to postulate a MoA, which is further evaluated as to weight of evidence and human relevance (Meek et al. 2014). AOPs provide a framework for capturing existing knowledge concerning the linkage (linear sequence) of key events between a molecular initiating event and cancer, bringing a biological understanding to the mechanisms of carcinogenesis and identifying data gaps (Sachana and Leinala 2017). The Organization for Economic Co‐operation and Development is investigating the utility of AOPs in regulatory decision‐making (Sachana and Leinala 2017). Mechanisms of carcinogenesis have been distilled into 10 key characteristics of carcinogens (Smith et al. 2016), which are used to integrate what is known about carcinogen MoA into traits corresponding to the hallmarks of cancer (Hanahan and Weinberg 2011). The Halifax project is aimed at transforming cancer risk assessment paradigms from single agent type consideration to one that acknowledges the cumulative effects of low‐dose exposures to chemical mixtures (Miller et al. 2017; Bopp et al. 2019). These cancer risk evaluation strategies are being supported by new “omics” technologies (Buesen et al. 2017). For example, there are efforts to create *in vitro* transcriptional biomarkers to assist in identification of carcinogens and elucidating MoA (Jonker et al. 2009; Hochstenbach et al. 2012; Reis et al. 2016; Li et al. 2017).

The International Conference on Harmonization (ICH) guidance documents M3, S1A, S1B, S1C, S2, and S6 identify regulatory requirements for carcinogenicity testing of new pharmaceuticals. The ICH S1A guidance defines the conditions under which carcinogenicity studies of human pharmaceuticals should be conducted to provide consistency in worldwide regulatory assessments of applications (see https://www.ich.org/products/guidelines/safety/article/safety-guidelines.html). ICH S1B provides guidance on approaches for evaluating the carcinogenic potential of pharmaceuticals. In the past, the requirements for carcinogenicity testing were frequently met by performing 2‐year tumor bioassays in rats and mice. Over the past two decades, however, FDA's regulatory requirement for carcinogenicity testing was most often met using a 2‐year rat study and a 6‐month transgenic mouse study, an approach that reduced the use of animals (Jacobs and Brown 2015). Currently, a proposal to modify the ICH S1 Guidance [S1(R1)] is being considered by the ICH Association, which comprises regulatory agencies and industry members around the world (see https://www.ich.org/about/members-observers.html). The proposal identifies the conditions under which the rat 2‐year bioassay could be waived without impacting patient safety, specifically based on evaluation of hormonal perturbation, the results of genetic toxicology assessments, and the findings of a 6‐month transgenic mouse carcinogenicity study (Sistare et al. 2011; van der Laan et al. 2017). The proposal to modify guidelines for carcinogenicity testing is, at least in part, a response to criticism of high‐dose single agent testing in animals as a poor model for human cancer risk, particularly given the expense, time and large numbers of animals required (Alden et al. 2011; Friedrich and Olejniczak 2011).

Thus, there is a recognized need for *in vivo* biomarkers that can be integrated with other information and predict the carcinogenic impact of intermittent and chronic exposures. It would be invaluable to be able to predict the tumorigenic response of lifetime rodent exposures from shorter term studies (28 days to 6 months; Parsons 2018a). Measurements of hCDMs from subchronic repeat‐dose rodent studies could provide highly cancer‐relevant information, which would complement analyses of genotoxicity, mode of action, human susceptibility, organ‐specific effects, as well as absorption, distribution, metabolism, and excretion in a weight of evidence approach.

### Theoretical Advantages of CDMs as Biomarkers of Cancer Risk

The most consequential advantage of CDMs as biomarkers of cancer risk is their inherent relevance to carcinogenesis in both animals and humans. CDMs are causally implicated in oncogenesis and confer a growth advantage (positive selective advantage) on the cancer cell in the microenvironment of the tissue in which the cancer arises (Stratton et al. 2009). Functionally, therefore, CDMs cause clonal expansion. Many lines of evidence, accumulating over decades, have led to the conclusion that CDMs are causal and necessary for carcinogenesis (Vogelstein et al. 2013). More recently, technological advances in NGS and other types of genetic analyses have provided clinicians with tools to aid in the identification and management of cancer. Knowledge describing how CDMs impact tumor phenotype is being derived from clinical investigations, and bioinformatic tools are available, which allow clinicians to interpret the clinical significance of variants detected by NGS (Tamborero et al. 2018).

Analyses of mutations in CD genes have been incorporated into all aspects of clinical oncology (see Supporting Information Table S1). A comprehensive summary of how CDMs are being used in oncology is beyond the scope of this review. Instead, instructive examples are provided (Table S1), which illustrate the range of clinical applications based on analyses of CDMs. The examples illustrate how detection of CDMs is being utilized in cancer screening, cancer diagnosis (i.e., distinguishing benign from malignant neoplasms), cancer prognosis, selecting the most effective available therapy for individual patients (personalized cancer treatment), and managing the development of resistance to therapy. In recent years, significant advances have been made in identifying genetic alterations that predict patient responses. In non‐small‐cell lung cancer patients, for example, *EGFR* exon 19 deletions and the L858R mutation impact the efficacy of erlotinib, afatinib, or gefitinib; *EGFR* T790M mutation impacts the efficacy of osimertinib; and *BRAF* V600E impacts the efficacy of dabrafenib in combination with trametinib. In melanoma patients, *BRAF* V600E impacts the efficacy of vemurafenib or dabrafenib. Matching patient tumor mutations to therapy is leading to improved patient outcomes. As a second‐ or third‐line therapy, for example, osimertinib is effective for treating T790M mutation‐positive non‐small‐cell lung cancer patients, with median overall survival of 26.8 months (with 12‐, 24‐ and 36‐month survival rates of 80%, 55%, or 37%, respectively; Ahn et al. 2019).

It has been shown that preexisting, subpopulations of cells carrying CDMs can cause resistance to therapy (Diaz Jr et al. 2012). The outgrowth of untargeted resistance mutants is now recognized as a major obstacle to achieving durable patient responses to molecularly targeted therapies. Measurements of CDMs in circulating tumor DNA (ctDNA) isolated from the blood of cancer patients is being assessed to monitor disease burden, to predict the development of resistance, and to identify when it is beneficial to alter therapy (Garcia‐Murillas et al. 2015; Siravegna et al. 2015; Van Emburgh et al. 2016). The analysis of ctDNA as a means to monitor minimally residual disease holds great promise and its value is being investigated in clinical trials, but it is not yet used commonly in the management of cancer patients (Ahlborn and Østrup 2019). The clinical importance of CDMs underscores their relevance to cancer phenotypes and provides justification for developing CDMs as biomarkers of cancer risk.

An important advantage of CDMs as biomarkers is that the mutations potentiate clonal expansion, an obligatory characteristic of carcinogenesis. Clonal expansion of driver mutations has the potential to amplify their signal, making them more sensitive biomarkers of cancer risk than neutral reporter gene mutations or passenger mutations.

Additional advantages of CDMs as cancer risk biomarkers are related to the interpretation of changes in CD mutant fraction ([MF] for a particular gene sequence is calculated as the number of mutant alleles in a sample divided by the total number of mutant and wild‐type alleles in the sample). There is an extensive amount of information available on CDM prevalence in human tumors and the cellular functions of wild‐type and mutant proteins. The occurrence of CDMs in tumors is well described and searchable in a variety of databases [Catalogue of Somatic Mutations in Cancer (COSMIC), https://cancer.sanger.ac.uk/cosmic]; The Cancer Genome Atlas (TCGA), NIH, National Cancer Institute Genomic Data Commons Data Portal, https://portal.gdc.cancer.gov/; International Cancer Genome Consortium (ICGC), https://dcc.icgc.org/]. Searchable databases describe CDM pathway participation, functions of wild‐type CD proteins, and how their functions relate to the hallmarks of cancer, including OMIM (the Online Mendelian Inheritance in Man, resource of The National Center for Biotechnology Information; https://www.omim.org/), GeneCards (GeneCards® Human Gene Database, https://www.genecards.org/), and KEGG (KEGG: Kyoto Encyclopedia of Genes and Genomes, https://www.genome.jp/kegg/).

Assuming an appropriate CDM (or panel of CDMs) has been selected relevant to the analysis of a specific tumor type, increases in CD MF can straightforwardly be interpreted as key events in an AOP. This can be contrasted with biomarkers based on changes in gene expression or methylation, which have the potential to be adaptive responses, rather than adverse events. Although an increase in a CD MF in an individual does not guarantee a tumor will develop in that individual, it does denote an increased probability a cancer will develop (i.e., increased cancer risk). Therefore, for groups of exposed rodents or human populations, an increase in load of CD mutations is expected to correlate with an increased tumor burden.

Another major advantage of CDMs as biomarkers of cancer risk is that they are primarily DNA based measurements and, thus, can be performed on any tissue from any species from which enough DNA can be isolated. Indeed, studies may analyze CDMs in DNA isolated from many types of samples, including fresh frozen tissues, formaldehyde‐fixed paraffin embedded tissues, single cells and circulating tumor cells, as well as free circulating DNA isolated from plasma. Furthermore, using justifiable assumptions of mutant zygosity (e.g., heterozygosity for a dominant oncogene mutation), CD MF can be straightforwardly translated into a mutant cell number or proportion, information that may be useful for mathematical modeling (Soh et al. 2009).

### Error‐Corrected NGS Will Enable Analyses of Panels of hCDMs

The development of EC‐NGS methods is revolutionizing the field of genetic toxicology (see review by Salk and Kennedy in this same issue) and has created an opportunity to analyze panels of amplicons encompassing many hCDMs at the same time. The error correction incorporated within these methods is based on molecularly tagging individual molecules, repeatedly sequencing them, bioinformatically sorting molecules with the same unique molecular identifier into read families, then discarding base changes not well conserved within a family as errors. Base changes that are well conserved within a read family (consensus sequences) are compared to a reference sequence to identify mutations. A variety of methods for EC‐NGS have been developed (Salk et al. 2018). Methods based on construction of single‐strand consensus sequences or two‐strand consensus sequences have been reported to detect MFs between 10^−3^ and 10^−6^ (Kinde et al. 2011; Gregory et al. 2015; Young et al. 2015). Duplex sequencing, which is based on constructing double‐strand consensus sequences, has been reported to detect mutant fractions as low as 10^−8^ (Salk et al. 2018). Thus, analyses of panels of hCDMs are now an achievable goal and their development as biomarkers should proceed.

CDMs can and have been used individually to assess the carcinogenic potential of test articles (Parsons 2018a). Dose‐related increases in the MF of single CDMs can be sufficient to establish that a test article has carcinogenic potential. However, individual analytical methods based on the detection of single CDMs are unlikely to be applicable across all target organs or useful reporters for all types of mutagens. Developing panels that include mutations represented across tumor sites and all possible mutational specificities, therefore, is a more robust approach.

### Cancer‐Specific Prevalence of Mutated CD Genes and hCDMs

Analyses over the last 20 years have yielded a quantitative understanding of CDM levels across normal tissues and tumors, particularly for hCDMs. The literature indicating CDMs are prevalent in normal human tissues and occur in remarkably high percentages of tumors as mutant subpopulations are presented here, along with a systematic analysis of the most highly mutated genes, based on COSMIC data.

Table 1 identifies 94 different CD genes that are the most prevalent in the 10 deadliest cancers (top 20 mutated genes for each cancer type and mutations within a particular codon that account for at least 1% of tumors, for each gene sequenced). Because cancer rates differ in different countries, this review used worldwide cancer rates to identify the 10 deadliest cancer types. The 10 deadliest cancers worldwide and the number of deaths due to each cancer type in 2017 (Table 1) were extracted from Roth et al. (2018). The COSMIC list of top 20 mutated genes for each cancer type considers the total number of mutants reported, as well as the percentages of characterized genes determined to be mutant. In this analysis of the 10 deadliest tumor types, leukemia and non‐Hodgkin's lymphoma are represented by the COSMIC mutational analyses of acute myelogenous leukemia and diffuse large B‐cell lymphoma, respectively, because these were the largest searchable and relevant classifications within COSMIC. COSMIC mutational analyses described in this review are based on COSMIC v87 and v88, which were released on November 13, 2018, and March 19, 2019, respectively. For the 94 CD genes identified in Table 1, gene IDs, gene aliases, and the functions ascribed to each gene (primarily extracted from GeneCards) are provided in Table S2. Potential sources of uncertainty in these analyses are the unreported sensitivities of the mutation detection methods underlying studies compiled in COSMIC, which are likely to be variable, and the continual incorporation of new data into COSMIC.

**Table 1:** Top 20 Mutated Genes Reported in COSMIC^a^ for the 10 Deadliest Cancers

Neoplasm site or type		Trachea, bronchus, and lung	Colon and rectum	Stomach	Liver	Breast	Pancreas	Esophagus	Prostate	Leukemia^b^	Non‐Hodgkin lymphoma^c^
2017 deaths (×1000)		1883.1	896.0	865.0	819.4	611.6	441.1	436.0	415.9	347.6	248.6
Tissue‐specific mutant gene ranking (percent representation of reported mutations)	1	*TP53* (38)	*APC* (47)	*TP53* (34)	*TP53* (27)	*PIK3CA* (27)	*KRAS* (64)	*TP53* (52)	*TP53* (17)	*NPM1* (32)	*KMTD* (29)
	2	*EGFR* (27)	*TP53* (45)	*LRP1B* (15)	*CTNNB1* (20)	*TP53* (27)	*TP53* (44)	*LRP1B* (12)	*SPOP* (8)	*FLT3* (24)	*BCL2* (23)
	3	*LRP1B* (21)	*KRAS* (34)	*FAT4* (13)	*TERT* (20)	*CDH1* (11)	*SMAD4* (15)	*CDKN2A* (10)	*AR* (7)	*DNMT3A* (19)	*TP53* (21)
	4	*KRAS* (16)	*FAT4* (18)	*ARID1A* (12)	*ARID1A* (8)	*GATA3* (10)	*CDKN2A* (13)	*KMT2D* (10)	*FOXA1* (7)	*NRAS* (13)	*CREBBP* (17)
	5	*KEAP1* (11)	*LRP1B* (15)	*CDH1* (11)	*LRP1B* (8)	*KMT2C* (8)	*CTNNB1* (20)	*FAT1* (8)	*PTEN* (6)	*TET2* (12)	*MYD88* (15)
	6	*FAT4* (10)	*PIK3CA* (14)	*PIK3CA* (9)	*AXIN* (7)	*ESR1* (6)	*GNAS* (5)	*NOTCH1* (8)	*KMT2C* (5)	*CEBPA* (10)	*SOCS1* (15)
	7	*KMT2C* (8)	*TGFBR2* (14)	*PREX2* (9)	*ARID2* (6)	*ERBB2* (5)	*ARID1A* (5)	*PIK3CA* (7)	*FAT4* (4)	*IDH2* (9)	*PIM1* (15)
	8	*CDKN2A* (8)	*ACVR2A* (12)	*KMT2C* (9)	*CDKN2A* (4)	*PTEN* (4)	*LRP1B* (4)	*FAT4* (6)	*LRP1B* (4)	*RET* (10)	*EZH2* (12)
	9	*FAT1* (8)	*KMT2C* (11)	*TGFBR2* (9)	*FAT4* (4)	*ARID1A* (4)	*RNF43* (4)	*NFE2L2* (5)	*KMT2D* (4)	*WT1* (9)	*B2M* (13)
	10	*STK11* (7)	*ZFHX3* (11)	*KMT2D* (8)	*KMT2C* (4)	*TBX3* (4)	*TGFBR2* (4)	*EP300* (5)	*ZFHX3* (4)	*KIT* (8)	*CD79B* (10)
	11	*KMT2D* (7)	*BRAF* (10)	*TRRAP* (7)	*TSC2* (4)	*NCOR1* (4)	*APC* (4)	*KMT2C* (5)	*APC* (3)	*TP53* (8)	*TNFAIP3* (10)
	12	*RB1* (7)	*KMT2D* (10)	*PTPRT* (6)	*ATM* (4)	*NF1* (4)	*KMT2C* (4)	*PREX2* (4)	*ATM* (3)	*ASXL1* (8)	*CARD11* (9)
	13	*NF1* (6)	*FBXW7* (10)	*RNF43* (6)	*NFE2L2* (3)	*SPEN* (4)	*ZNF521* (3)	*FBXW7* (4)	*CTNNB1* (3)	*RUNX1* (8)	*STAT6* (9)
	14	*PTPRT* (6)	*SMAD4* (10)	*KRAS* (6)	*PREX2* (3)	*KMT2D* (3)	*KMT2D* (3)	*PTPRT* (4)	*CDK12* (3)	*MAP2K2* (8)	*CDKN2A* (8)
	15	*SMARCA4* (6)	*TRRAP* (9)	*ERBB3* (6)	*PIK3CA* (3)	*RB1* (3)	*RBM10* (3)	*CREBBP* (3)	*KRAS* (3)	*PBRM1* (8)	*TET2* (7)
	16	*GRIN2A* (6)	*ARID1A* (9)	*SPEN* (6)	*KMT2D* (3)	*LRP1B* (3)	*ATM* (3)	*ERBB4* (3)	*PIK3CA* (2)	*ATM* (8)	*KMT2C* (7)
	17	*ARID1A* (5)	*RNF43* (9)	*ZFHX3* (6)	*TRRAP* (3)	*MAP2K4* (3)	*PIK3CA* (3)	*PTCH1* (3)	*BRCA2* (2)	*IDH1* (6)	*TNFRSF14* (7)
	18	*ATM* (5)	*FAT1* (9)	*APC* (5)	*PTPRB* (3)	*AKT1* (3)	*BRCA2* (2)	*ARID1A* (3)	*GRIN2A* (2)	*SRSF2* (6)	*BTG1* (7)
	19	*ZNF21* (5)	*TCF7L2* (8)	*RHOA* (5)	*APC* (3)	*FOXA1* (3)	*FAT4* (2)	*ZFHX3* (3)	*SPEN* (2)	*PTPN11* (6)	*LRP1B* (7)
	20	*PREX2* (5)	*PREX2* (8)	*FAT1* (5)	*KMT2A* (3)	*RUNX1* (3)	*ACVR2A* (2)	*BRCA2* (3)	*RB1* (2)	*DNM2* (6)	*PRDM1* (6)

a COSMIC v87, 28 February 2019, database (https://cancer.sanger.ac.uk/cosmic). This analysis includes tumor cell lines.

b Acute myeloid leukemia.

c Diffuse large B‐cell lymphoma.

**Figure 1:**
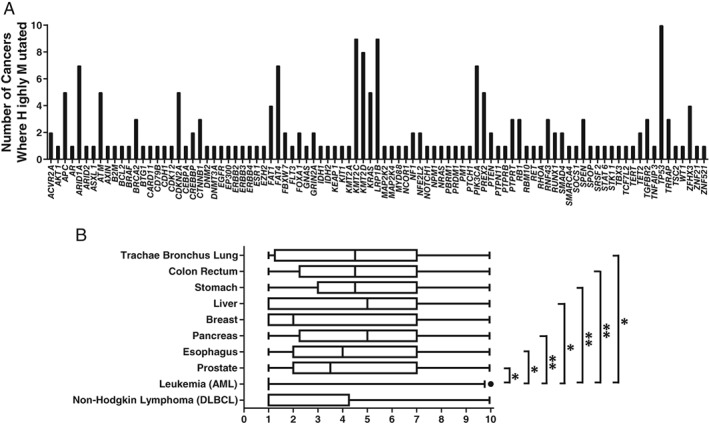
Distribution and sharing of highly mutated CD genes across the 10 deadliest cancers. (**A**) Number of cancers in which each of the 94 highly mutated CD genes are found (genes identified in Table 1). (**B**) Analysis of the degree to which top 20 mutant CD genes are shared across the 10 deadliest cancers. For each cancer type, its top 20 mutated genes were assigned values (from 1 to 10) based on the number of cancers in which each gene is among the top 20 most mutated. The box and whisker plots (whiskers represent the 5th and 95th percentiles) show that AML has significantly fewer shared CD genes relative to other cancer types. Significance levels are indicated as: *P value between 0.01 and 0.05; **P value between 0.001 and 0.005.

Figure 1A shows that some mutated CD genes occur in more than one type of cancer, while others are unique to one cancer. Table S3 identifies the tumor types in which CD genes containing hotspot mutations accounting for ≥1% of tumors occur. The most frequently mutated CD genes detected in multiple tumor types are *ARID1A*, *FAT4*, *KMT2C*, *KMT2D*, *KRAS*, *LRP1B*, *PIK3CA*, *PREX2*, and *TP53*, all of which are identified as Tier 1 genes in the COSMIC Cancer Gene Census. *TP53* was the only gene detected in all 10 of the deadliest cancers. Acute myeloid leukemia (AML) is unique among the 10 deadliest cancers because its most mutated genes are not shared with other cancers (except *TP53*). A Kruskal–Wallis analysis with Dunn's multiple comparison test indicated there were significant differences among cancers in terms of numbers of shared mutant CD genes (*P* = 0.0002), with AML significantly different from cancers of the trachea/bronchus/lung, colon/rectum, stomach, liver, pancreas, esophagus, and prostate (see Fig. 1B). This is consistent with observations for AML relative to other cancer types as reported by Kandoth et al. (2013). Specifically, it was shown that AML had fewer significantly mutated genes relative to 11 other cancer types and AML had the lowest number of mutations per megabase of DNA. Together, these observations indicate leukemias may follow a different carcinogenic path than other cancers. Perhaps, they have more large‐scale structural rearrangements than other cancers (Kandoth et al. 2013).

Not all mutations detected in tumors would be useful early biomarkers of cancer risk if incorporated into a panel of hCDMs. Mutations that are among the most prevalent will be the most useful because they will serve as reporters for the largest fraction of cancers achievable for a given panel size. All CD genes identified in Table 2 were analyzed for hotspot mutations using COSMIC data (this analysis excluded mutations detected within cell lines). Hotspot mutational targets were defined as codons mutated in at least 1% of each cancer analyzed. Although a prevalence of 1% is low, this cutoff was selected for the analysis based on the idea of casting a large net and the knowledge that genes with a prevalence of 1% as determined by standard DNA sequencing will likely represent larger percentages of tumors when methods that can detect mutant subpopulations are employed (Parsons and Myers 2013b; Myers et al. 2015). Hotspot codons were identified within 40 of the 94 genes listed in Table 2. Thus, no hotspot mutational target was identified in 54 highly mutated CD genes. Table S4 identifies the codons within CD genes that are hotspots for mutation, the cancer types in which the hotspot mutations are observed, the percentages of cancers having a hotspot mutant codon, and the percentages of mutant cancers represented by a hotspot codon.

**Table 2:** Incidence of CD Genes with Hotspots

Gene ID	Number of cancer types where the gene is among top 20 mutated and represents > 1% of cancers	COSMIC mutated gene incidence across the 10 deadliest cancers (percent)^a^	Mutated gene incidence based on ACB‐PCR measured mutant subpopulations (percent)^b^
*TP53*	10	13.58	
*KRAS*	5	11.77	Breast: 13.33 Colon: 63.63 Lung: 90.90 Thyroid: 47.06
*PIK3CA*	4	4.56	Breast: 48.88 Colon: 45.00 Lung: 41.67 Thyroid: 45.00
*RET*	1	3.67	
*BCL2*	1	2.47	
*CTNNB1*	2	2.14	
*PBRM1*	1	1.95	
*APC*	1	1.42	
*MYD88*	1	1.32	
*NRAS*	1	1.24	
*MAP2K2*	1	1.19	
*DNMT3A*	1	1.09	
*BRAF*	1	0.97	Breast: 78.89
*ATM*	1	0.90	
*SOCS1*	1	0.88	
*IDH2*	1	0.87	
*CD79B*	1	0.80	
*SRSF2*	1	0.80	
*KIT*	1	0.70	
*STAT6*	1	0.65	
*GNAS*	1	0.62	
*DNM2*	1	0.57	
*ESR1*	1	0.57	
*EZH2*	1	0.57	
*AR*	1	0.48	
*SPOP*	1	0.47	
*EGFR*	1	0.43	
*CREBBP*	1	0.40	
*SMAD4*	2	0.36	
*FBXW7*	1	0.33	
*ZNF521*	1	0.30	
*B2M*	1	0.29	
*AKT1*	1	0.24	
*BTG1*	1	0.21	
*RBM10*	1	0.21	
*FLT3*	1	0.19	
*TGFBR2*	1	0.19	
*IDH1*	1	0.16	
*RHOA*	1	0.15	
*ERBB2*	1	0.14	

a For each gene, the total incidence of hotspot mutations (percentage of total cancers mutated, see Table S3) was added for the 10 cancer types, then the total incidence was divided by 10, to express hCDM representation as a percentage of the 10 deadliest cancers.

b Percentages of cancers with MFs greater than the upper 95th confidence level of that present in normal tissue is provided. For *KRAS*, the values indicate percentages of tumors with *KRAS* G12D, G12V, or both. For *PIK3CA*, the values indicate percentages of tumors with *PIK3CA* H1047R, E545K, or both. For *BRAF*, the values indicate percentages of tumors with *BRAF* V600E presented from previously published ACB‐PCR analyses, along with the cancer types analyzed (Myers et al. 2016; Parsons et al. 2017).

**Figure 2:**
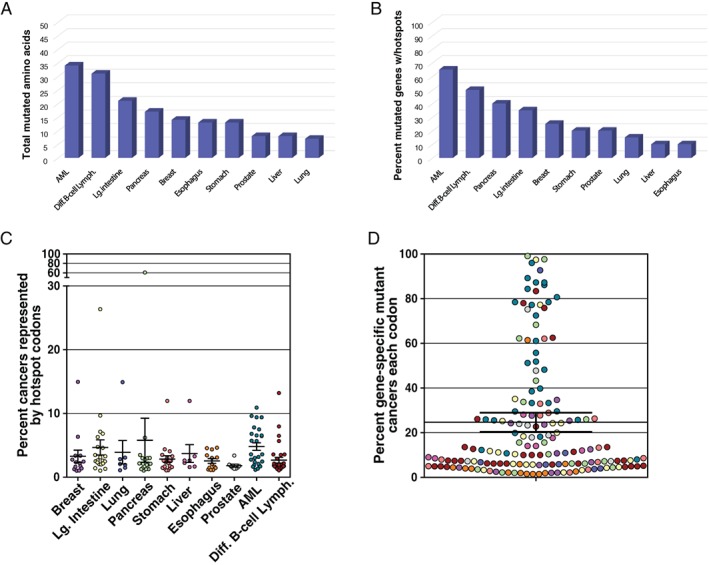
Prevalence of hotspot codon targets within the top 20 most mutated CD genes, analyzed by cancer type. The numbers of mutants occurring at specific codons within cancers reported in the COSMIC database were analyzed (different mutations occurring at the same codon were combined). Some of the most mutated CD genes did not contain hotspots for mutation. The percentages of the 20 most mutated genes for which hotspot codons were identified is depicted in (**A**), for the different cancer types. The total numbers of hotspot codons in the top 20 most highly mutated genes for each cancer type are shown in (**B**). The fractions of total tumors represented by identified hotspot codons are shown in (**C**). The factions of mutated tumors represented by identified hotspot codons are shown in (**D**), with MFs color coded by tumor type, using the color coding identified as in (**C**).

The different types of cancer varied in terms of the percentages of their highly mutated genes that were found to possess a mutational hotspot; percentages ranged from 10% to 65% (2/20 CD genes in esophagus and liver cancers had hotspots, whereas 13/20 CD genes in AML had hotspots, see Fig. 2A). The number of codons that were identified as hotspots for mutation in the top 20 genes for each cancer type are shown in Figure 2B. The percentage of gene‐specific mutations that each mutant codon represents is depicted in Figure 2C. For example, *KRAS* codon 12 mutations are detected in 60% of pancreatic cancers (Fig. 2C and Table S4 “Percent of Total Cancers the Codon Represents”), whereas *KRAS* codon 12 mutations account for 92.74% of *KRAS*‐mutated pancreatic cancers (Fig. 2D and Table S4 “Percent of Gene‐specific Mutant Cancers each Codon Represents”). Also, the highly mutated CD genes varied in terms of the number of mutational hotspots they encompass; 18 of 40 CD genes listed in Table S3 had only one mutational hotspot. The CD gene that encompassed the largest number of mutational hotspots was *TP53*, in which 16 different codons are mutated in at least 1% of the 10 cancers analyzed. A total of 108 different mutational hotspot codons were detected in the 40 genes identified in Table S3. The COSMIC database includes reports of 424 different missense or nonsense mutations within these 108 hotspot codons (along with 37 silent mutations).

Hotspot mutations within these codons account for almost 63,000 COSMIC cancers, with the greatest number ascribed to large intestine cancers (~31,000) and the least ascribed to prostate and esophageal cancers (~500 each). Table 2 ranks CD genes in the 10 deadliest cancers based on the fraction of cancers with hCDMs. For example, *TP53* hotspot mutations were detected in all 10 cancers, with a percent incidence of 13.58%. The percent incidence was calculated as the sum of percentages of total cancers each codon represents for a given gene, dividing by 10, to summarize representation across the 10 cancer types (see Table S4). All the genes identified in Table 2 are categorized as COSMIC Tier 1 genes, meaning they have a documented activity relevant to cancer, along with evidence that their mutations change the activity of the gene product in a way that promotes oncogenic transformation. The synthesis of COSMIC data presented in Table 2 indicates that, on average, a hCDM in at least one of these 40 genes is expected to be present ~60% of the time in the 10 deadliest cancers.

It might be presumed that the genes containing hCDMs would be enriched in oncogenes as compared to tumor suppressor genes (TSGs) because the traditional oncogene mutation causes a gain of function and has a dominant phenotype, whereas the traditional TSG causes loss of function and is recessive (may require both copies of the gene to be inactivated to confer a selective advantage). Sondka et al. (2018) describe how COSMIC Tier 1 and Tier 2 genes were classified in the COSMIC Cancer Gene Census and how the above view of oncogenes and TSGs does not accurately capture the complexity of CD genes. *TP53* provides a good example of the complexity in categorizing a gene as an oncogene or TSG; while generally viewed as a TSG, *TP53* has also been described as “an oncogene in disguise” (Soussi and Wiman 2015). Although TSGs comprise a greater portion of the COSMIC Tier 1 genes than oncogenes (140 vs. 79, respectively; Sondka et al. 2018), the 40 genes identified as encompassing hCDMs were enriched in oncogenes (22 genes were identified in the COSMIC Gene Census as oncogenes, 5 identified as having evidence of both oncogene and TSG functions, with only 13 were identified as TSGs). Several of the TSGs were identified as dominant. While this distribution of oncogenes and TSGs is interesting, it should be noted that the approach used to select CD genes containing hotspots was agnostic with respect to function, rather it was simply based on reported mutational prevalence in COSMIC.

There are two limitations of the analysis that should be noted. First, because the analysis is solely based on submissions to the COSMIC database, which is enriched in coding sequences compared to noncoding regions, the approach will not capture promoter mutations. Another potential caveat to this analysis was raised by a report indicating that hotspot passenger mutations can occur via APOBEC3A‐mediated mutagenesis (Buisson et al. 2019). This report provided examples of true hCDMs, as well as APOBEC‐induced hotspot passenger mutations. The 40 gene targets identified in our analysis included some of the former, but none of the latter.

**Figure 3:**
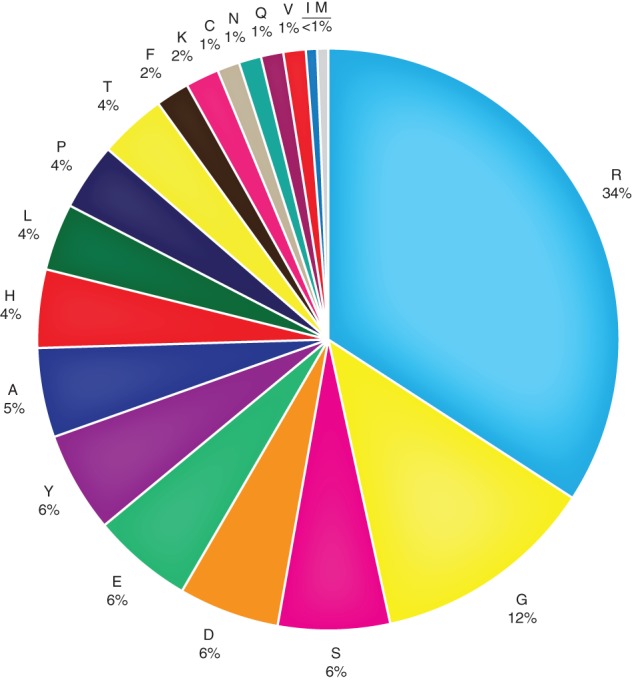
Distribution of amino acids encoded by hotspot mutational targets. The data summarize all the hotspot mutational targets identified in Table S4. The figure presents the distribution of amino acids encoded by hotspot mutational targets observed across all 10 cancer types. Distributions for individual cancer types are provided in Figure S1.

Different amino acid substitutions are known to impact protein function in different ways (Preeprem and Gibson 2014). Therefore, the extent to which different amino acids serve as targets for hCDMs was examined. The distribution of amino acids encoded by the mutational hotspots varied across the different cancer types and is reported in Figure S1. Figure 3 and Figure S1 show that arginine is the predominant amino acid target for mutation in the identified hotspots. For example, all five hotspots detected in APC coded for arginine (R213, R216, R976, R114, and R1450). The observation that arginine is the predominant hotpot for mutation across the most highly mutated COSMIC CD genes is consistent with the observed loss of arginine on the proteome level for 2000 protein coding genes from the Cancer Cell Line Encyclopedia (Tsuber et al. 2017). Internal arginine residues are abundant in proteins and have the unique capacity to remain protonated in environments otherwise incompatible with charge; this makes them useful in situations where a charge is needed in the interior of a protein (Harms et al. 2011). Thus, substitution of another amino acid in place of arginine likely disrupts protein structure.

Alternatively, arginine codons may be more mutable targets than other codons because they are enriched in CpG sites. The germ line mutation rate is considerably higher for cytosines at CpG sites than for any other nucleotide in the human genome (Mugal and Ellegren 2011), and 4 of 6 arginine codons contain a CpG sites (0.66%) compared to 8 of 64 for all codons (0.13%).

### COSMIC Underreports the Prevalence of hCDMs, When Mutant Subpopulations Are Considered

Data from the COSMIC database, including that described above, have been generated using standard Sanger or NGS sequencing methods. Generally, these technologies have sufficient sensitivity to detect variant allele fractions between 1% and 100% (Stead et al. 2013; Strom 2016; Williams et al. 2018). When more sensitive mutation detection methods are employed, significantly larger percentages of tumors are identified as mutant (Parsons et al. 2010; Myers et al. 2014; Myers et al. 2015; Myers et al. 2016; Parsons et al. 2017; Myers et al. 2019). For example, *KRAS* G12D and G12V were reported to occur in 6.9% and 8.2% of colon tumors described in the COSMIC database but were detected in 48.6% and 51.4% of colon tumors analyzed by ACB‐PCR, which has a sensitivity of 10^−5^ (Parsons and Myers 2013b). In COSMIC, the *KRAS* G12D and G12V mutations are reported to occur in 3.3% and 4.1% of lung adenocarcinomas but were detected in 47.6% and 57.1% of lung adenocarcinomas analyzed by ACB‐PCR, respectively (Myers et al. 2015). In COSMIC, *KRAS* G12D and G12V are reported to occur in only 0.6% and 0.2% of papillary thyroid carcinomas but were detected in 88.2.0% and 58.8% of papillary thyroid carcinomas analyzed by ACB‐PCR, respectively (Myers et al. 2014). Furthermore, a significant portion of tumors analyzed in these studies carried both *KRAS* mutations (G12D and G12V): colon, 29.7%; lung, 28.6%; and thyroid, 58.8% (Parsons and Myers 2013b; Myers et al. 2014; Myers et al. 2015).

Mutant tumor subpopulations have been studied in ductal carcinomas and invasive ductal carcinomas of the breast (Myers et al. 2016; Myers et al. 2019). According to the COSMIC database, *PIK3CA* H1047R, *PIK3CA* E545K, *KRAS* G12D, *KRAS* G12V, *HRAS* G12D, and *BRAF* V600E mutations occur in 10.5%, 5.41%, 0.31%, 0.16%, 0%, and 0% of invasive ductal carcinomas, respectively (Myers et al. 2019). However, when invasive ductal carcinomas were analyzed by ACB‐PCR, these mutations were observed in 42%, 36%, 63%, 11%, 86%, and 59% of invasive ductal carcinomas, respectively (Myers et al. 2019). Furthermore, all 81 tumors analyzed in the study contained subpopulations of three or more of the six mutations analyzed (Myers et al. 2019).

There are a number of observations to consider regarding the interpretation of mutant subpopulations. First, mutations that are driving carcinogenesis are expected to occur in cancers at levels exceeding that present in normal tissue. There is a literature supporting the idea that mutant subpopulation can be important for driving tumor progression. Tumor cells of different subtypes and different mutational profiles can interact to enhance tumor growth (Cleary et al. 2014), and a minor mutant subpopulations can drive the growth of the predominant tumor cell population (Inda et al. 2010). There are a several mechanisms that could explain how mutant tumor subpopulations arise, including (1) acquisition of the mutation occurring at a late stage of tumor development with subsequent positive selection, (2) multiclonal tumor initiation with positive selection of one clone exceeding the other, or (3) multiclonal tumor initiation with negative selection of one clone as the tumor microenvironment changes during tumor progression. The hCDMs that initiate carcinogenesis and become enriched early in tumorigenesis may be selected against in advanced cancers. Support for this comes from the observation that *KRAS* G12V mutation is less abundant in colorectal adenocarcinomas than adenomas and the inverse correlations observed between *KRAS* G12V MF and maximum tumor dimension for both colon and thyroid cancers (Losi et al. 2009a; Parsons et al. 2010; Parsons and Myers 2013a).

**Figure 4:**
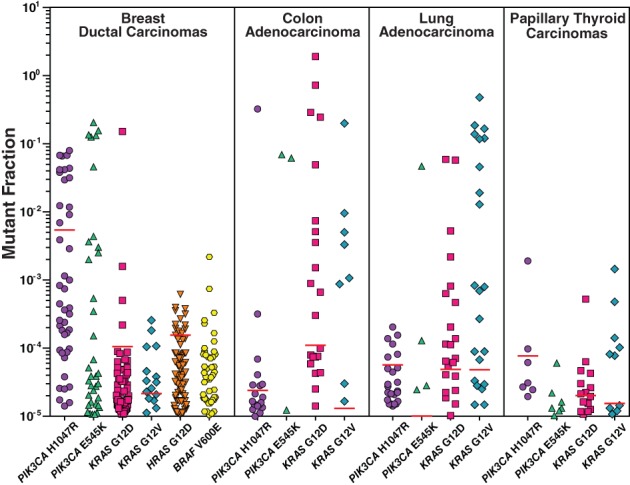
HCDMs are prevalent as mutant tumor subpopulations at levels above the upper 95th confidence level of that in the corresponding normal tissue. A synthesis of published ACB‐PCR data is presented (Parsons et al. 2010; Myers et al. 2014; Myers et al. 2015; Myers et al. 2016; Parsons et al. 2017; Myers et al. 2019). Cancers with mutant tumor subpopulations ≥10^−5^ are plotted. Red lines denote the upper 95th confidence limit for the same hCDM measured in the corresponding normal tissue. The absence of a red line indicates the upper 95th confidence limit for the CDM was below 10^−5^. Correction added on 18 October 2019, after first online publication: Figure 4 has been revised to change labels from “Adenocarcino‐” to “Adenocarcinoma”.

ACB‐PCR has been used to analyze DNA from normal tissues and different tumor types. Each ACB‐PCR assay includes concurrently analyzed MF standards and the assays have the same sensitivity (10^−5^). Therefore, the relative abundance of hCDMs can be compared across studies and sample types. For a limited number of hCDMs, the prevalence of mutations in tumors, including subpopulations, was compared to that of normal samples, and the percentages of cancers with hCDM levels greater than the upper 95th confidence interval of that measured in the corresponding normal tissue were extracted (see Fig. 4 and Table 2).

Together, these studies demonstrate that (1) large percentages of tumors carry multiple CD mutant subpopulations and (2) some hCDMs are present in much larger proportions of tumors than indicated in COSMIC, when mutant subpopulations are considered. Thus, the conclusion derived from the COSMIC analysis that at least one of the 40 CD genes identified in Table 2 is expected to be present in ~60% of the 10 deadliest cancers is an underestimate. From these data, therefore, we conclude that multiple hCDMs can be selected that, together, can well represent the breadth of human cancers.

### Prevalence of CDMs in Normal Human Tissues

It is now well established that CDMs are present in normal tissues. Sensitive, and in some cases quantitative, technologies have been used to study the prevalence of hCDMs in normal tissues. Specifically, allele‐specific PCR methodologies, droplet digital PCR, and high‐accuracy NGS methodologies have been used for this purpose. The data obtained using these technologies indicate that hCDMs are prevalent in the DNA of normal tissues, including breast (Myers et al. 2016), buffy coat (Young et al. 2016), colon (Parsons et al. 2010; Parsons et al. 2017), endometrium (Suda et al. 2018), esophagus (Martincorena et al. 2018; Yokoyama et al. 2019), hematopoietic stem/progenitor cells (Welch et al. 2012), lung (Gao et al. 2009; Myers et al. 2015), peripheral leukocytes (Wong et al. 2015), thyroid (Myers et al. 2014), skin (Martincorena et al. 2015), tracheal–bronchial epithelium (Sudo et al. 2006), and uterine lavage samples (Salk et al. 2019). In his book chapter “How do mutant clones expand in normal tissue,” Brash indicates that clones of mutant cells often occur at high frequency in human skin, breast, lung, colon, pancreas and blood, the clones seem to be early stages in cancer development, and clonal expansion appears to be driven by physiological events affecting the entire tissue, rather than by the mutant cell acquiring additional mutation (Brash 2016). Hafner et al. have reported that seborrheic keratoses carrying *bona fide* oncogenic mutations lack malignant potential (Hafner et al. 2010). Also, it has been recognized that progression of clones is impacted by genetic makeup, preexisting differentiation state, and signals that are received from the surrounding microenvironment (Graham et al. 2011; Marusyk et al. 2012). Thus, mutant clones in the well‐structured environment of normal tissue may not progress without additional events.

The prevalence of hCDMs in normal tissues and how levels in normal tissue relate to cancer risk can be illustrated by a few examples. In normal esophagus, somatic mutations accumulated with age (age is the most important risk factor for cancer) and a strong positive selection was observed for clones carrying 14 cancer genes, such that tens to hundreds of clones were detected per square centimeter (Martincorena et al. 2018). A sensitive and quantitative allele‐specific PCR approach (ACB‐PCR) was used to measure *PIK3CA* H1047R mutation in normal breast tissues from cancer‐free individuals, focusing on this mutation because it is the most prevalent hCDM detected in breast carcinomas. It was determined that 4 of 10 normal breast tissues had MFs >10^−2^, meaning ~1:50 normal breast cells in these samples were *PIK3CA* H1047R mutant (Myers et al. 2016). Importantly, measurements of four different hCDMs in four different normal human tissues (breast, colon, lung, and thyroid) revealed that interindividual variability in normal tissues is correlated with hCDM importance in tissue‐specific carcinogenicity, as measured by the frequency with which each hCDM is detected in cancers that developed from the corresponding normal tissue (Parsons et al. 2017). This is consistent with the idea that the hCDMs prevalent in normal tissues contribute in the carcinogenesis of those tissues because they confer a tissue‐specific selective advantage. Importantly, this result also indicates that the degree of interindividual variability can be used to identify the hCDMs that will be most relevant biomarkers for studying tissue‐specific carcinogenesis, because the variability likely reflects both clonal expansion and the stochastic nature of carcinogenesis (Parsons et al. 2017).

### Integrating Knowledge Regarding the Nature of hCDMs with a Field Cancerization View of Carcinogenesis

Our understanding of how to use hCDMs as biomarkers of cancer risk must be grounded upon evidence regarding how such mutations accumulate during carcinogenesis and a valid understanding of carcinogenesis itself. For many decades, it was assumed/expected that CDMs would be ultra‐rare in normal tissues and that all tumors were the outgrowth of a single initiated cell, with acquisition of additional mutations producing tumor heterogeneity during progression (Nowell 1976; Fearon et al. 1987; Weinberg 2013). These preconceptions were based on data derived using available technologies of the time, which constituted primarily of analyses of fully developed tumors using relatively insensitive technologies.

**Figure 5:**
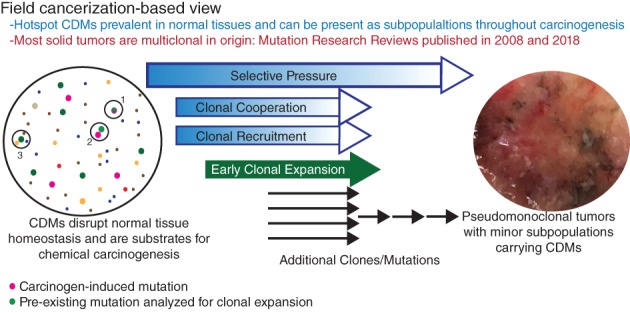
Integrating properties of hCDMs with a field cancerization view of carcinogenesis. The coloring of “clones” is meant to represent different mutations, with pink indication mutations induced by genotoxic carcinogens and green indicating clones carrying spontaneous, preexisting mutations. Three potential avenues to carcinogenesis are depicted. Genotoxic carcinogens may induce mutations (pink) in preexisting clones carrying hCDMs (green) (1). Genotoxic carcinogens may induce genetic or epigenetic lesions in cells adjacent to pre‐existing clones hCDMs (2), with either event leading to clonal expansion of cells carrying hCDMs. Also, spontaneous tumor induction (3) may occur through interaction between mutant clones, including those carrying hCDMs (green). Expansion of clones carrying hCDMs may also be driven by exposure to non‐genotoxic carcinogens. References are Parsons 2008 and Parsons 2018b.

Figure 5 depicts a field cancerization view of carcinogenesis based on more recent data obtained using more powerful technologies. Figure 5 reflects the assumption that most solid tumors are initiated by more than one clone of cells. This assumption is supported by evidence obtained from the re‐analyses of X‐chromosome inactivation studies, information from chimeric and lineage tracing mouse models, and studies identifying clonal cooperation in tumor initiation, progression, and metastasis (Parsons 2008; Parsons 2018b). Evidence that most solid tumors are multiclonal in origin stands on its own, apart from recognition that multiclonality is more consistent with how measurements of CDMs fluctuate through stages of carcinogenesis. Blood cancers may be the exception. Although there is evidence of multiclonal blood cancers, they may occur as smaller percentages of blood cancers compared to solid cancers (Parsons 2018b). In this regard, the analysis presented in Figure 1A is interesting; it shows leukemia shares significantly fewer of the highly mutated CD genes than are shared among solid tumor types.

Figure 5 incorporates previously summarized information, specifically (1) hCDMs are prevalent in normal tissues, (2) hCDM levels in normal tissue correlate with their carcinogenic impact, as indicated by tumor representation, (3) subpopulations carrying hCDMs are remarkably prevalent across cancers, and (4) evidence that some hCDMs are selected against in advanced cancers. Mutations may arise by chance, but mutant clones may persist and expand early in carcinogenesis because the mutations confer a tissue‐specific selective advantage. Clearly, not all hCDMs progress to cancer. Indeed, the abundance of hCDMs in normal tissues indicates that most are insufficient to produce a cancer. Thus, Figure 5 depicts a spatially stochastic model of tumor initiation, where cooperating clones must occur within sufficiently close proximity to support each other's participation in tumor initiation. This idea is supported by examples of clonal recruitment during tumor progression (Thliveris et al. 2013; McCreery and Balmain 2016). Selective advantage is expected to shift during carcinogenesis, as the tumor microenvironment changes. Some cooperating clones may be necessary participants in tumor initiation, but no longer required or selected against, once more robust and potentially multiply‐mutated clones are produced.

In terms of serving as biomarkers of cancer risk that can be assessed in subchronic rodent studies, hCDMs that are abundant in normal tissues may be the ideal early reporters of carcinogenesis. As prevalent preexisting mutations, they may be generic reporters of clonal expansion due to cooperation with clones carrying other carcinogen‐induced mutations (in the case of genotoxic carcinogens). A report by Cha et al. (1994) provided proof of principle that exposure to a genotoxic carcinogen [*N*‐nitroso‐*N*‐methylurea (NMU)] could induce clonal expansion of preexisting *Hras* mutants. Specifically, they observed that NMU did not increase the number of *Hras* mutant‐positive sectors of NMU‐treated rat mammary tissue compared to controls but did increase the number of *Hras* mutants in positive sectors. The authors concluded that NMU‐induced *Hras* mutant tumors arose from preexisting *Hras* mutants, with the carcinogen directly or indirectly responsible for the clonal expansion associated with tumor formation. Potentially, clonal expansion due to non‐genotoxic, carcinogen‐induced mechanisms (e.g., immunosuppression, induction of cell proliferation, frank cytotoxicity and regenerative proliferation, or epigenetic effects) may also be discerned using hCDM end points.

### CDMs in Cell Communication

The view of carcinogenesis presented in Figure 5 can serve as a basis for testing the hypothesis that hCDMs are drivers of tumor initiation and early clonal expansion because they confer the ability to cooperate with other carcinogen‐induced clones (or become insensitive to the repression of co‐localized wild‐type cells). To test this hypothesis, the four forms of cell communication were compared with that of all COSMIC Tier 1 and Tier 2 CD genes. The four types of cell communication found in multicellular organisms are autocrine, juxtacrine, paracrine, and endocrine (Robert 2015). These refer to the release of a chemical signal or ligand that targets the releasing cell itself (autocrine, intracellular communication), targets adjacent cells *via* adherens, tight, or gap junctions (juxtacrine), targets cells near the releasing cell via ligand diffusion through extracellular matrix (paracrine), or targets cells elsewhere in the body (endocrine/hormonal). Thus, mutations in genes that have functions related to juxtacrine, paracrine, or endocrine cell communication have the potential to impact carcinogenesis through a mechanism that involves clonal cooperation.

**Figure 6:**
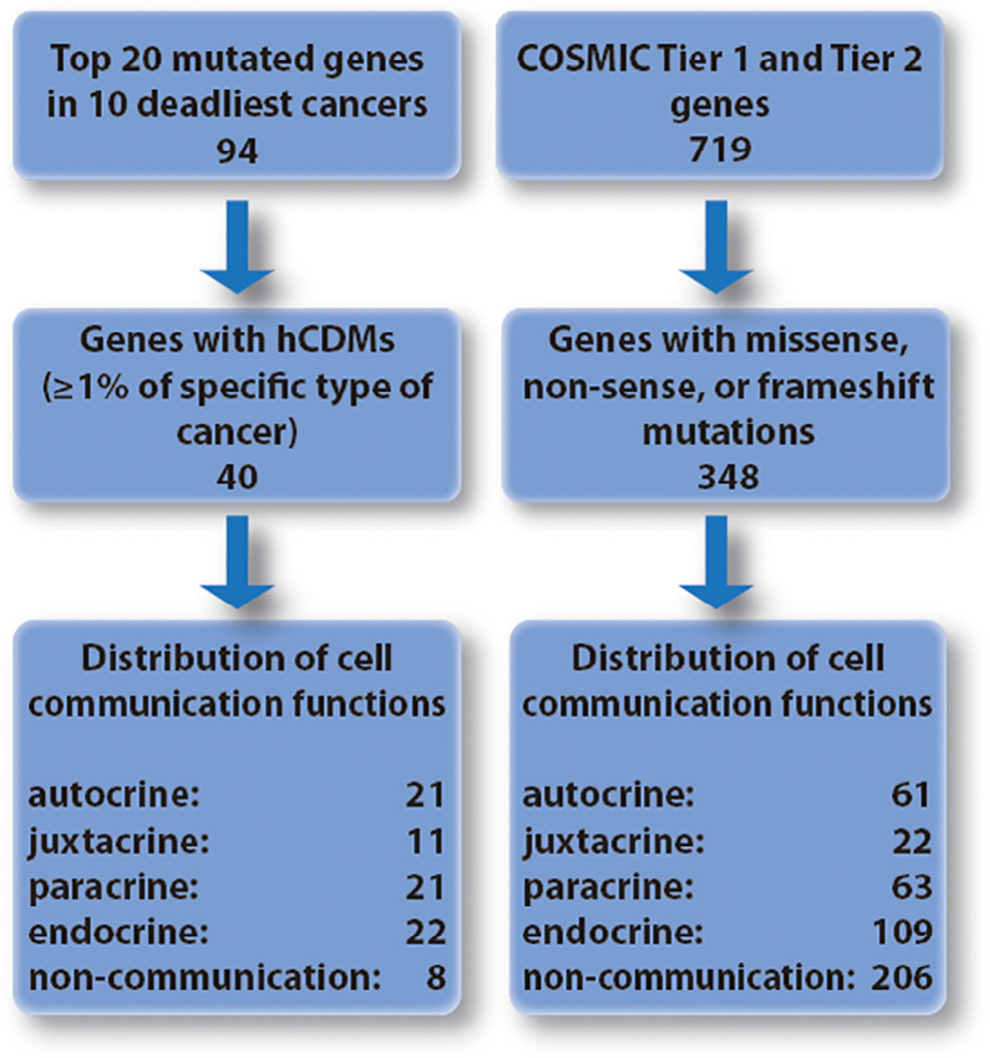
Schematic of the analysis performed to determine the subset of genes carrying hCDMs are enriched in functions related to intercellular communication compared to all COSMIC Tier 1 and Tier 2 targets of point mutation.

Our analysis began by collecting COSMIC Tier 1 and Tier 2 genes (n = 719 genes) and filtering them to include only gene targets of missense, nonsense, or frameshift mutations (based on COSMIC data). This generated a subset of 348 genes that are comparable to the genes encompassing hCDMs (see Fig. 6). This subset included *BCL2*, *BTG1*, and *STAT6*, which were identified as targets of translocation in the COSMIC list of Tier 1 and Tier 2 genes but were determined to be targets of point mutation by our analysis of hotspot mutations. Both groups of genes were analyzed to identify which have functions related to any of the four forms of cell communication.

Information used to identify and categorize gene function in cell communication was derived from multiple sources. OMIM (the Online Mendelian Inheritance in Man, resource of The National Center for Biotechnology Information; https://www.omim.org/) was searched using the terms “autocrine,” “paracrine,” “juxtacrine,” “endocrine,” and “hormonal.” Similarly, it was searched using the individual gene IDs. The involvement of gene products in cellular pathways was visualized using KEGG: Kyoto Encyclopedia of Genes and Genomes (https://www.genome.jp/kegg/) and reactome (https://reactome.org/). Finally, literature searches were conducted, searching for the cell communication terms (“autocrine,” “paracrine,” “juxtacrine,” and “endocrine” and “hormonal”) in combination with each gene ID. Genes were often found to be involved in more than one type of cell communication. References documenting associations between the 40 CD genes encompassing an hCDM and one or more of the forms of cell communication are provided in Table 3.

**Table 3:** Genes with hCDMs and Functions Related to Cell Communication

Gene ID	Cell communication function	Signaling pathway participation	References
*AKT1*	Autocrine; juxtacrine; paracrine; endocrine	PI3 kinase signaling pathway	Seo et al. 2016, Armstrong and Drummond‐Barbosa 2018, Ju et al. 2007, Singh et al. 2016
*APC*	Autocrine; juxtacrine; paracrine; endocrine	Wnt–β‐catenin signaling pathway	Zhu et al. 2014, Ølstørn et al. 2003, Buller et al. 2015, Schinner et al. 2009
*AR*	Autocrine; juxtacrine; paracrine; endocrine	Androgen receptor signaling pathway	Ware et al. 2014, Lydka et al. 2011, Takuwa et al. 2018, Tanner et al. 2011
*ATM*	Juxtacrine; paracrine; endocrine	PI3 kinase signaling pathway	Lang et al. 2018, Chen et al. 2015, Karayazi Atici et al. 2017
*B2M*	Autocrine	Class I MHC mediated antigen processing and presentation pathway	
*BCL2*	Autocrine	NF‐kappa B signaling pathway; HIF‐1 signaling pathway; sphingolipid signaling pathway; p53 signaling pathway; PI3K‐Akt signaling pathway; Hedgehog signaling pathway; JAK–STAT signaling pathway; estrogen signaling pathway; parathyroid hormone synthesis, secretion, and action	Bold et al. 2001
*BRAF*	Autocrine; endocrine	MAPK signaling pathway	Huntington et al. 2004,Chernaya et al. 2018
*BTG1*		Cell cycle pathway	
*CD79B*		B‐cell receptor signaling pathway	
*CREBBP*		cAMP signaling pathway	
*CTNNB1*	Juxtacrine; paracrine; endocrine	Wnt signaling pathway; Hippo signaling pathway; Rap1 signaling pathway	Bai et al. 2013, Boyer et al. 2012, Youngblood et al. 2018
*DNM2*		Phospholipase D signaling pathway; Toll‐like receptor pathway; neurotrophic receptor Sauer et al. 2017 signaling pathway	
*DNMT3A*		Chromatin organization pathway	
*EGFR*	Autocrine; juxtacrine; paracrine; endocrine	MAPK signaling pathway; PI3K‐Akt signaling pathway; JAK–STAT signaling pathway	Chen et al. 2018, Dubé et al. 2012, Zhang et al. 2017, Sauer et al. 2017
*ERBB2*	Autocrine; juxtacrine; paracrine; endocrine	ErbB signaling pathway	Li et al. 2004, Hofer et al. 1996, Kim et al. 2011, Chowdhury et al. 2017
*ESR1*	Autocrine; endocrine	Estrogen receptor mediated signaling pathway	
*EZH2*	Autocrine; paracrine; endocrine	Oxidative stress induced senescence pathway	Hartman et al. 2013, Dudakovic et al. 2016, Mathieu et al. 2018
*FBXW7*	Endocrine	Notch signaling pathway	Sancho et al. 2014, Mathieu et al. 2018
*FLT3*	Autocrine; paracrine	MAPK signaling pathway; Ras signaling pathway; PI3K‐Akt signaling pathway	Zheng et al. 2004, Markovic et al. 2012
*GNAS*	Endocrine	cAMP signaling pathway	Jin et al. 2019
*IDH1*	Paracrine	Citrate cycle TCA cycle pathway	Mao and Leonardi 2019
*IDH2*	Paracrine	Citrate Cycle TCA cycle Pathway	Mao and Leonardi 2019
*KIT*	Autocrine; paracrine	MAPK signaling pathway; Ras signaling pathway; Rap1 signaling pathway; PI3K‐Akt signaling pathway; phospholipase D signaling pathway	Li et al. 2018a, Kim et al. 2018
*KRAS*	Autocrine; juxtacrine; paracrine; endocrine	MAPK signaling pathway; Ras signaling pathway; PI3K‐Akt signaling pathway; ErbB signaling pathway	Zhu et al. 2014, McKenzie et al. 2016, Liu et al. 2016, Chamberlain et al. 2014
*MAP2K2*	Endocrine	MAPK signaling pathway; Ras signaling pathway; PI3K‐Akt signaling pathway; cAMP signaling pathway	Kim et al. 2018
*MYD88*	Autocrine; paracrine; endocrine	MAPK signaling pathway; NFκβ signaling pathway	Cataisson et al. 2012, Perkins et al. 2018, Guo et al. 2016
*NRAS*	Endocrine	MAPK signaling pathway; Ras signaling pathway; ErbB signaling pathway; PI3K‐Akt signaling pathway; mTOR signaling pathway	Argyropoulou et al. 2018
*PBRM1*		MAPK signaling pathway	
*PIK3CA*	Autocrine; paracrine; endocrine	ErbB signaling pathway; Ras signaling pathway; Rap1 signaling pathway; cAMP signaling pathway; Chemokine signaling pathway; HIF‐1 signaling pathway; FoxO signaling pathway; mTOR signaling pathway; PI3K‐Akt signaling pathway; AMPK signaling pathway; apoptosis signaling pathway; VEGF signaling pathway; JAK–STAT signaling pathway; T‐cell receptor signaling pathway; TNF signaling pathway	Thakur and Ray 2017, Young et al. 2015, Stratikopoulos et al. 2019
*RBM10*	Autocrine	Notch signaling pathway	
*RET*	Autocrine; juxtacrine; paracrine; endocrine	RET signaling pathway	Li et al. 2009a, Mazzaferri et al. 1996, Shabtay‐Orbach et al. 2015, Horibata et al. 2018
*RHOA*	Autocrine; juxtacrine; paracrine; endocrine	Ras signaling pathway; cAMP signaling pathway; mTOR signaling pathway; Wnt signaling pathway	Zhao et al. 2014, Hendrick and Olayioye 2019, Daubriac et al. 2018, Petersen et al. 2018
*SMAD4*	Autocrine; juxtacrine; paracrine; endocrine	TGF‐β signaling pathway	Shiou et al. 2006, Wang et al. 2019, Jiang et al. 2015, Li et al. 2018b
*SOCS1*	Autocrine; paracrine; endocrine	JAK–STAT signaling pathway; Insulin signaling pathway; Prolactin signaling pathway	Niwa et al. 2018, Jørgensen et al. 2013, Chan et al. 2014
*SPOP*		Hedgehog signaling pathway	
*SRSF2*		RNA metabolism pathway	
*STAT6*	Autocrine; paracrine	JAK–STAT signaling pathway	Olsan et al. 2011, Passerini et al. 2018
*TGFBR2*	Paracrine; endocrine	TGF‐β signaling pathway	Li et al. 2009b, Busch et al. 2015
*TP53*	Endocrine	MAPK signaling pathway; p53 signaling pathway; PI3K‐Akt signaling pathway; Wnt signaling pathway	Rieber and Strasberg‐Rieber 2014
*ZNF521*	Endocrine	Cell cycle pathway	Addison et al. 2014

Of the 40 genes with hCDMs, this analysis ascribed functions related to autocrine/intercellular communication for 21, juxtacrine/extracellular communication for 11, paracrine/extracellular communication for 21, endocrine/extracellular communication for 22, and 8 with functions unrelated to cell communication. Of the 348 COSMIC Tier 1 and Tier 2 genes encompassing point mutations, this analysis ascribed functions related to: autocrine/intercellular communication for 61, juxtacrine/extracellular communication for 22, paracrine/extracellular communication for 63, endocrine/extracellular communication for 109, and 206 with functions unrelated to cell communication (see Fig. 6 and Table 3). According to chi‐square analysis, this indicates a difference in functions related to cell communication between the two groups (*P* ≤ 0.0001). When the two groups were compared in terms of the numbers of genes involved in either autocrine, juxtacrine, paracrine, and endocrine signaling using Fisher's exact test (two‐sided), significant enrichment of functions related to cell communication was observed in the genes identified as encompassing hCDMs compared to Tier 1 and Tier 2 genes with point mutations (autocrine, *P* < 0.0001; juxtacrine, *P* = 0.001; paracrine, *P* < 0.0001; and endocrine, *P* = 0.0043). Conversely, Tier 1 and Tier 2 genes with point mutations were enriched in genes without functions related to cell communication compared to the genes identified as encompassing hCDMs (206/348 vs. 9/40, respectively, *P* < 0.0001). The literature indicates that many CD gene products impact more than one type of cell communication. In Figure S2, a number of CD genes that have been associated with different combinations of the types of cell communication are depicted. The percentages indicated in Figure S2 correspond to each subset relative to the total number of genes considered, including those without known function in cell communication (40 for hotspot CD genes or 348 for COSMIC Tier 1 and 2 genes, respectively).

This analysis, therefore, supports the hypothesis that hCDMs can be drivers of tumor initiation and progression by altering communication between clones with complementing phenotypes. However, it cannot be ruled out that the results were influenced by publication bias, given that the subset of hCDM‐containing genes includes many extensively investigated genes.

### How Measurements of Panels of hCDMs Could Be Validated as Biomarkers of Cancer Risk and Incorporated into Cancer Risk Assessment

There are three possible approaches whereby measurements of hCDMs could improve cancer risk assessment: (1) by enabling prediction of chronic tumor incidence (bioassay results) from panel MF measurements, (2) by providing a cancer‐relevant point of departure (PoD) for low‐dose extrapolation or margin of exposure type risk assessments, and (3) strengthening the scientific knowledge underpinning decision‐making related to rodent to human extrapolation.

**Figure 7:**
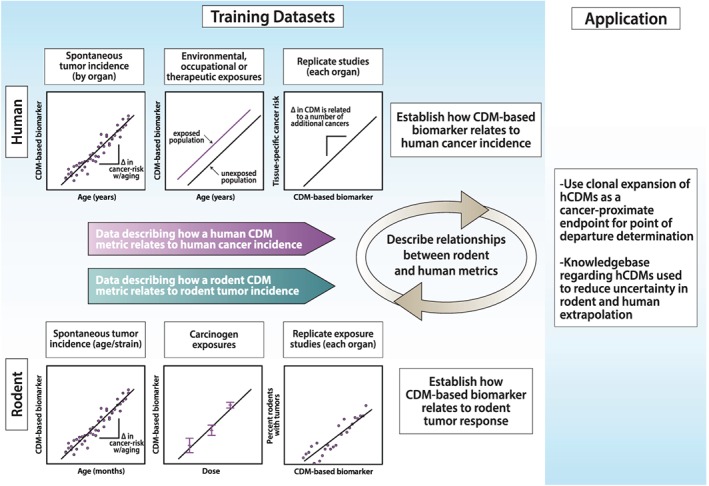
Strategies to incorporate knowledge of hotspot CD MF into cancer risk assessment. The figure depicts experimental paradigms that could be used to relate tumor incidence in human or rodent to a metric based on analyzing batteries of hCDMs. The metric could be used to predict rodent tumor response based on relationships to other carcinogens with known potency or provide a cancer‐relevant point of departure for dose extrapolation. Data on the same cancer‐relevant metric for rodent and human could reduce uncertainty in rodent to human extrapolation.

If panels of hCDMs are to be used to predict rodent tumor incidences associated with chronic exposures, then (1) training sets of rodent samples subchronically exposed to carcinogens and for which tumor bioassay outcomes are known must be developed and (2) a metric based on panel hCDM measurements must be derived that can be related to percentages of rodents that develop tumors. Once such relationships are established, they must be tested with additional rodent samples exposed to carcinogens to validate the metric as a meaningful and reproducible reporter of carcinogenic potential. This will need to be done in an organ‐specific manner (see Fig. 7).

Examples exist of correlations between measurements of hCDMs following carcinogen exposure and tumor incidences due to longer exposures for rodents treated with the same doses of carcinogens (Parsons 2018a). This analysis showed that the more proximate the measurement of mutation was to the scoring of tumors, the better the observed correlation. These data illustrate the idea of relating measured MF at a particular dose to tumor response measured at the same dose. However, cancer risk predictions cannot be made based on the analysis of a single hCDM. Instead, a composite metric based on a battery of hCDM measurements and data from multiple carcinogen exposures are needed to meaningfully relate a hCDM‐based metric to rodent tumor responses in the corresponding tissues. Using a panel encompassing many hCDMs and the types of analyses discussed above, it should be possible to discern meaningful relationships between a metric obtained from subchronic exposures and experimentally established chronic tumor responses (see Fig. 7). If this is achieved and rodent tumor responses can be predicted from the subchronic exposures (28‐day to 6‐month repeat‐dose studies), this would eliminate the need to conduct additional 2‐year rodent tumor bioassays.

The second way that hCDMs could improve cancer risk assessment would be to incorporate these cancer‐relevant measurements into current risk assessment paradigms. Increasingly, non‐tumor data are being incorporated into cancer risk assessment, including observations of mutation in CD genes (Albertini et al. 2003; MacGregor et al. 2015). An increase in a metric based on a battery of hCDMs could be used as a PoD in quantitative cancer risk assessment. The International Workshops on Genotoxicity Testing subgroup working group on Quantitative Approaches to Genetic Toxicology Risk Assessment examined the relationship between the BMD_10_ (the benchmark dose responsible for a 10% response) for cancer induction with the BMD_5_ for *in vivo* micronucleus induction, where data of both types were available for 25 agents (MacGregor et al. 2015). The group concluded there was a general correlation between cancer induction and mutagenic and/or clastogenic damage for agents thought to act via a genotoxic mechanism. Dose–response has been analyzed for key events along the path to carcinogenesis: DNA adducts, mutations, preneoplasia, and tumors (Fukushima et al. 2018). Induction of hCDMs due to clonal expansion is expected to be a more proximate event to cancer than neutral gene mutations or clastogenic effects, a step away from that which occurs in pre‐neoplastic lesions. Consequently, they may constitute a more relevant end point for PoD determination. The degree to which batteries of hCDMs acting through different pathways will be able to report on the actions of carcinogens that operate through various modes of action is currently an open question.

The third way in which hCDMs could be used to improve cancer risk assessment is by providing a sound scientific basis for rodent to human extrapolation. Human CD MF in specific tissues of populations with different risk profiles (profiles related to age, familial cancer syndromes, or known carcinogen exposures) could be correlated with human tumor incidence and then juxtaposed with the analogous panel measurements from rodents (see Fig. 7). In this regard, data on age‐associated cancer risk from the SEER database will be particularly useful (https://seer.cancer.gov/statistics/). Valuable data could be developed through analyses of human populations from different geographical regions, with and without occupational exposures, or individuals with therapeutic exposures vs. controls, provided differences in cancer incidence are known for the different subgroups. Several studies have already demonstrated that human mutational load increases as a function of human age (Myers et al. 2016; Martincorena et al. 2018; Salk et al. 2019). Takeshima and Ushijima (2019) write that “recent technological advancements have enabled measurement of rare point mutations, and studies have shown that their accumulation levels are indeed correlated with cancer risk.”

One aspect of rodent to human extrapolation where hCDMs could provide a scientific foundation relates to the use of uncertainty factors (UFs). UFs are incorporated into cancer risk assessment “based on the assumption that a sufficient reduction in exposure from those at the boundary for the onset of adverse effects will yield a safe exposure level” (Dankovic et al. 2015). Five UFs may be used when setting an acceptable daily intake or occupational exposure limit to account for (1) differences in uncertainty between animals and the average human, (2) differences in the average human and the most sensitive subpopulation, (3) uncertainty in estimating a no effect level if a lowest observable effect dose was used to determine the PoD, (4) extrapolating from a short‐duration study to a life‐time exposure, and (5) the possibility that the most sensitive end point may not have been analyzed. Measurements of analogous, panels of hCDMs in rodent and human samples could (1) inform the extent to which rodents and humans are different in their background levels of hCDMs at different ages, (2) provide data on the variability in CD gene MFs between individual humans at the same age, compared to that within and across different rodent strains (inbred and outbred), and (3) show whether or not panels of hCDMs are more sensitive end points of carcinogenic potency (due to clonal expansion) than other end points. Potentially, the development of such data could reduce uncertainty in species extrapolation and possibly justify replacing default UFs with better estimates. Importantly, using an EC‐NGS approach, acquisition of data on clonal expansion of hCDM targets can be obtained while simultaneously collecting data on non‐hotspot mutations to inform test article‐induced mutation spectra.

While the approaches proposed and illustrated in Figure 7 may be an oversimplification of how panels encompassing hCDMs are eventually validated as biomarkers, the use of panels, the idea of correlating MF measurements with tumor response (rodent) or cancer risk (human), and the measurement of hCDM‐encompassing amplicons using an EC‐NGS or high‐accuracy NGS approach are clearly on the horizon and gathering of the required knowledgebase must proceed. A key point to be investigated is what hCDMs are shared and have the same tissue specificity between human and rodents because EC‐NGS should focus on those. Compared to the richness of human tumor mutation databases, only a Sleeping Beauty Cancer Driver Database is publicly available for mouse (Newberg et al. 2018), although a collaboration between the Wellcome Sanger Institute and the National Toxicology Program may generate needed rodent tumor data (https://www.sanger.ac.uk/science/collaboration/mutographs-cancer-cruk-grand-challenge-project).

## CONCLUSIONS

This review summarizes literature relevant to the concept of using measurements of hCDMs (with mutant fractions measured in amplicon panels by EC‐NGS) as an approach to develop composite, molecular, safety biomarkers, with a context of use related to the analysis of test articles in short‐term rodent studies (see FDA guidance regarding the evidentiary requirements for biomarker qualification, https://www.fda.gov/regulatory-information/search-fda-guidance-documents/biomarker-qualification-evidentiary-framework).

Considerable evidence indicates that hCDMs are well suited to serve as biomarkers of cancer risk. Progress has been made toward understanding how population‐based or treatment group‐based increases in CD MF can be related to tumor incidence. It has been discerned that the analysis of interindividual variability can be used as an approach to identify the most relevant mutational targets for tissue‐specific carcinogenesis. However, many more studies examining the relationships between hCDMs and tumor responses are needed, with sufficiently large data sets to establish the mathematical relationships necessary for progress in this area and to provide the evidence needed for biomarker qualification (https://stm.sciencemag.org/content/9/417/eaal4599.short). This review can serve as a roadmap to guide such studies. Efficient development of this area is dependent upon investigating the CDMs that have the greatest potential to be useful biomarkers of cancer risk, and this review identified properties that will assist in selecting CDMs as components of a composite safety biomarker relevant to cancer risk.

**Figure 8:**
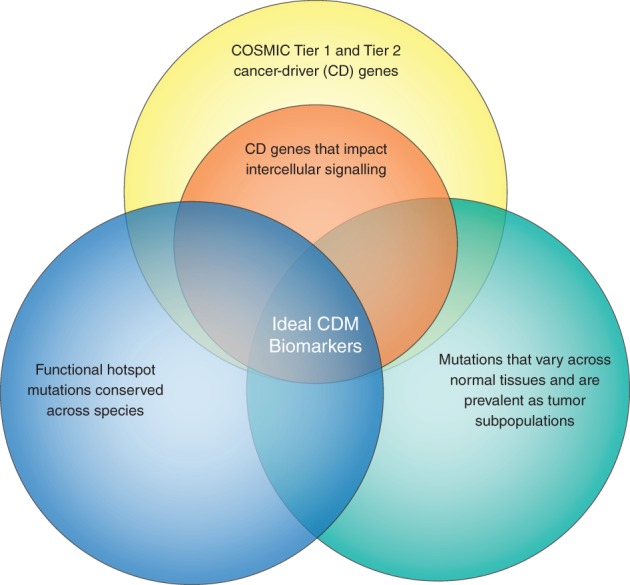
Properties expected to identify the ideal CDMs to incorporate into multipartite biomarkers of cancer risk.

The properties that we conclude will be the most important for the component targets of such a biomarker are shown in Figure 8. These include (1) authoritative designation as a CD gene, (2) identification of functional hotspots conserved across species, (3) targets that encode a wild‐type gene product with a function related to intercellular communication, and (4) experimental evidence indicating levels of the mutation vary across tissues (i.e., evidence of positive selection/clonal expansion) and are prevalent in tumors (including as subpopulations). The more prevalent the mutations in the panel, the larger percentages of cancers that could potentially be predicted by incorporating the complex biomarker (i.e., amplicon panel) measurements into subchronic repeat dose studies. The 40 mutational targets identified are all COSMIC Tier 1 genes, 30 have functions related to intercellular communication, and many of these genes are conserved across species. However, few have been analyzed with sufficient sensitivity in normal tissues and tumors to know whether they meet the criterion of prevalence in both situations. *KRAS* and *PIK3CA* CDMs have been shown to meet all the criteria indicated in Figure 8.

In conclusion, the outlook for progress in this area is promising. Furthermore, advances in the development and use of EC or high‐precision NGS methods (e.g., duplex sequencing) will speed data acquisition and, therefore, aid greatly in translating measurements of hCDMs into biomarkers that improve estimates of cancer risk.

## AUTHOR CONTRIBUTIONS

K.L. Harris analyzed which CD genes are involved in the different forms of cell communication. M.B. Myers performed the COSMIC database analyses of hCDMs. K.L. McKim collected GeneCard information on hCDMs. R.K. Elespuru summarized the status of carcinogenicity testing. B.L. Parsons contributed the remaining sections of the manuscript. All authors contributed to the figures, tables, and manuscript text. All authors approved the final manuscript.

## Supporting information


**Appendix S1**: Supporting Information
