# Supplementary material for: Rationale and Roadmap for Developing Panels of Hotspot Cancer Driver Gene Mutations as Biomarkers of Cancer Risk
Source: Environ Mol Mutagen. 2019 Oct 6;61(1):152–75. doi: 10.1002/em.22326 (PMC6973253; doi:10.1002/em.22326)

**Supporting Information Table 1. Representative examples describing how analyses of CDMs have been integrated into the practice of oncology.**

| **Oncology Application** | **Test;**  **Cancer Type** | **Sample Type Analyzed** | **CDMs Analyzed** | **CDM Interpretation (Reference)** |
| --- | --- | --- | --- | --- |
| Cancer screening | Cologuard; colorectal | Stool | *KRAS* | Patients with a positive test result should be referred for a diagnostic colonoscopy for further evaluation (Sweetser and Ahlquist 2017) |
|  | Inherited Cancer Pre-disposition; breast or ovarian | Blood or saliva | *BRCA1/2* | Women with pathogenic mutations able to make preventive choices to reduce cancer incidence, including preventative mastectomy or oophorectomy (Foulkes et al. 2015) |
| Cancer diagnosis | ThyroSeq v3; thyroid | Fine needle aspirate of thyroid nodules | Mutations in 115 genes | Improve diagnosis of atypia of undetermined significance, follicular lesion of undetermined significance, and follicular neoplasm/suspicious for follicular neoplasm without surgery (Ohori et al. 2019) |
|  | Targeted Sanger or NGS sequencing; pancreatic | Resected cysts or aspirate of cyst fluid | *KRAS*, *GNAS*, *CTNNB1*, *VHL* *DAXX*/*ATRX*, *MEN1*, *CDK2N2*, *RNF43* | Mutational biomarkers currently under investigation which may aid in diagnosis or sub-classification of pancreatic cystic neoplasms (Kulzer et al. 2018) |
| Cancer prognosis | Targeted sequencing; non-small cell lung cancer | Fresh frozen or formalin fixed tumor tissue | Mutations in 82 genes | Concurrent mutations in *TP53* and *STK11* were shown to confer poor survival in the *KRAS*-positive adenocarcinoma subgroup (La Fleur et al. 2019) |
|  | ARMS-PCR; colorectal | FFPE tumor samples | *KRAS* | Patients with left-side colorectal cancers have shorter overall survival than those with *KRAS* wild-type cancers (Xie et al. 2019) |
| Therapy selection | FoundationOne CDx comprehensive genomic profiling assay, solid tumors | FFPE tumor samples | Mutation in 324 genes | Detects mutational markers that predict responses to particular treatments (Ramanayake et al. 2019) |
|  | MSK-IMPACT; pancreatic | FFPE tumor samples and matched normal DNA | Mutations in 256 genes | Identified prevalent driver alterations typical of pancreatic ductal adenocarcinoma and low frequency genetic alterations in a wide variety of genes, some of which are targetable with  available agents. Activating mutations in *KRAS* are drivers of almost every pancreatic adenocarcinoma (Lowery et al. 2017) |
| Patient Monitoring | Ligation and BEAMing assays; colorectal | Circulating cell-free plasma DNA | *KRAS* | Pre-existing *KRAS* mutant tumor subpopulations account for the consistent development of resistance to EGFR blockage, occurring 5 to 6 months following treatment (Diaz Jr et al. 2012) |
|  | NGS, Sanger, and ddPCR; early breast cancer patients receiving neoadjuvant chemotherapy | Primary tumor and circulating cell-free plasma DNA | Mutations in exons of 273 genes | Circulating tumor DNA (ctDNA) in plasma was used to monitor for minimal residual disease, with a median 7.9 month lead time for predicting relapse (Garcia-Murillas et al. 2015) |

Diaz Jr LA, Williams RT, Wu J, Kinde I, Hecht JR, Berlin J, Allen B, Bozic I, Reiter JG, Nowak MA, Kinzler KW, Oliner KS, Vogelstein B. 2012. The molecular evolution of acquired resistance to targeted EGFR blockade in colorectal cancers. Nature 486:537.

Foulkes WD, Knoppers BM, Turnbull C. 2015. Population genetic testing for cancer susceptibility: founder mutations to genomes. Nature Reviews Clinical Oncology 13:41.

Garcia-Murillas I, Schiavon G, Weigelt B, Ng C, Hrebien S, Cutts RJ, Cheang M, Osin P, Nerurkar A, Kozarewa I, Garrido JA, Dowsett M, Reis-Filho JS, Smith IE, Turner NC. 2015. Mutation tracking in circulating tumor DNA predicts relapse in early breast cancer. Science Translational Medicine 7(302):302ra133.

Kulzer M, Singhi AD, Furlan A, Heller MT, Katabathina VS, McGrath KM, Zeh HJ, Zureikat A, Dasyam AK. 2018. Current concepts in molecular genetics and management guidelines for pancreatic cystic neoplasms: an essential update for radiologists. Abdominal Radiology 43(9):2351-2368.

La Fleur L, Falk-Sörqvist E, Smeds P, Berglund A, Sundström M, Mattsson JSM, Brandén E, Koyi H, Isaksson J, Brunnström H, Nilsson M, Micke P, Moens L, Botling J. 2019. Mutation patterns in a population-based non-small cell lung cancer cohort and prognostic impact of concomitant mutations in KRAS and TP53 or STK11. Lung Cancer 130:50-58.

Lowery MA, Jordan EJ, Basturk O, Ptashkin RN, Zehir A, Berger MF, Leach T, Herbst B, Askan G, Maynard H, Glassman D, Covington C, Schultz N, Abou-Alfa GK, Harding JJ, Klimstra DS, Hechtman JF, Hyman DM, Allen PJ, Jarnagin WR, Balachandran VP, Varghese AM, Schattner MA, Yu KH, Saltz LB, Solit DB, Iacobuzio-Donahue CA, Leach SD, Reilly EM. 2017. Real-Time Genomic Profiling of Pancreatic Ductal Adenocarcinoma: Potential Actionability and Correlation with Clinical Phenotype. Clinical Cancer Research 23(20):6094.

Ohori NP, Landau MS, Carty SE, Yip L, LeBeau SO, Manroa P, Seethala RR, Schoedel KE, Nikiforova MN, Nikiforov YE. 2019. Benign call rate and molecular test result distribution of ThyroSeq v3. Cancer Cytopathology 127(3):161-168.

Ramanayake N, Zein Y, Burn J, Levingston R, Hall G, Edwards M, Lim C, Delprado W, Turner J, Cheah A, Suthers G, Harraway J, Vargas C. 2019. Extended gene panels identify rare but potentially targetable mutations; initial experience at douglass hanly moir pathology. Pathology 51:S96.

Sweetser S, Ahlquist DA. 2017. Utility of the multitarget stool DNA test for detection of colorectal neoplasia. Colorectal Cancer 6(3):83-94.

Xie M-z, Li J-l, Cai Z-m, Li K-z, Hu B-l. 2019. Impact of primary colorectal Cancer location on the KRAS status and its prognostic value. BMC Gastroenterology 19(1):46.

**Supporting Information Table 2. Identification of cancer driver genes with hotspots, their aliases and functions^1, 2^**

| **Gene ID** | **Gene Name** | **Aliases** | **GeneCard^1^ Summary** |
| --- | --- | --- | --- |
| *ACVR2A* | Activin A Receptor Type 2A | Activin A Receptor Type 2A; Activin A Receptor, Type IIA; Activin Receptor Type-2A; EC 2.7.11.30; Activin A Receptor, Type II; Activin Receptor Type IIA; EC 2.7.11; ACTR-IIA; ACTRIIA; ACTRII | This gene encodes a receptor that mediates the functions of activins, which are members of the transforming growth factor-beta (TGF-beta) superfamily involved in diverse biological processes. The encoded protein is a transmembrane serine-threonine kinase receptor which mediates signaling by forming heterodimeric complexes with various combinations of type I and type II receptors and ligands in a cell-specific manner. The encoded type II receptor is primarily involved in ligand-binding and includes an extracellular ligand-binding domain, a transmembrane domain and a cytoplasmic serine-threonine kinase domain. This gene may be associated with susceptibility to preeclampsia, a pregnancy-related disease which can result in maternal and fetal morbidity and mortality. Alternative splicing results in multiple transcript variants of this gene. |
| *AKT1* | AKT Serine/Threonine Kinase 1 | AKT Serine/Threonine Kinase 1; V-Akt Murine Thymoma Viral Oncogene Homolog 1; RAC-Alpha Serine/Threonine-Protein Kinase; Protein Kinase B Alpha; Proto-Oncogene C-Akt;  Protein Kinase B; RAC-PK-Alpha; EC 2.7.11.1; PKB Alpha; PKB; RAC; V-Akt Murine Thymoma Viral Oncogene-Like Protein 1; Serine-Threonine Protein Kinase; Rac Protein Kinase Alpha; PKB-ALPHA; RAC-ALPHA; EC 2.7.11; AKT1m; PRKBA; CWS6; AKT | The serine-threonine protein kinase encoded by the *AKT1* gene is catalytically inactive in serum-starved primary and immortalized fibroblasts. AKT1 and the related AKT2 are activated by platelet-derived growth factor. The activation is rapid and specific, and it is abrogated by mutations in the pleckstrin homology domain of *AKT1*. It was shown that the activation occurs through phosphatidylinositol 3-kinase. In the developing nervous system AKT is a critical mediator of growth factor-induced neuronal survival. Survival factors can suppress apoptosis in a transcription-independent manner by activating the serine/threonine kinase AKT1, which then phosphorylates and inactivates components of the apoptotic machinery. Mutations in this gene have been associated with the Proteus syndrome. Multiple alternatively spliced transcript variants have been found for this gene. |
| *APC* | APC, WNT Signaling Pathway Regulator | Protein Phosphatase 1, Regulatory Subunit 46; Adenomatous Polyposis Coli Protein; Deleted In Polyposis 2.5; DP2.5; Adenomatosis Polyposis Coli Tumor Suppressor; Epididymis Secretory Sperm Binding Protein; Truncated Adenomatosis Polyposis Coli;  Adenomatous Polyposis Coli (APC); WNT Signaling Pathway Regulator; Adenomatosis Polyposis Coli; Adenomatous Polyposis Coli;  Protein APC; PPP1R46; BTPS2; DP2; DP3; GS | This gene encodes a tumor suppressor protein that acts as an antagonist of the Wnt signaling pathway. It is also involved in other processes including cell migration and adhesion, transcriptional activation, and apoptosis. Defects in this gene cause familial adenomatous polyposis (FAP), an autosomal dominant pre-malignant disease that usually progresses to malignancy. Disease-associated mutations tend to be clustered in a small region designated the mutation cluster region (MCR) and result in a truncated protein product. |
| *AR* | Androgen Receptor | Dihydrotestosterone Receptor; Nuclear Receptor Subfamily 3 Group C Member 4; NR3C4; DHTR; Spinal And Bulbar Muscular Atrophy; Testicular Feminization; Kennedy Disease; HUMARA; HYSP1; SMAX1; SBMA; AIS; AR8; TFM; KD | The androgen receptor gene is more than 90 kb long and codes for a protein that has 3 major functional domains: the N-terminal domain, DNA-binding domain, and androgen-binding domain. The protein functions as a steroid-hormone activated transcription factor. Upon binding the hormone ligand, the receptor dissociates from accessory proteins, translocates into the nucleus, dimerizes, and then stimulates transcription of androgen responsive genes. This gene contains 2 polymorphic trinucleotide repeat segments that encode polyglutamine and polyglycine tracts in the N-terminal transactivation domain of its protein. Expansion of the polyglutamine tract from the normal 9-34 repeats to the pathogenic 38-62 repeats causes spinal bulbar muscular atrophy (SBMA, also known as Kennedy's disease). Mutations in this gene are also associated with complete androgen insensitivity (CAIS). Alternative splicing results in multiple transcript variants encoding different isoforms. |
| *ARID1A* | AT-Rich Interaction Domain 1A | SWI/SNF-Related, Matrix-Associated, Actin-Dependent Regulator Of Chromatin Subfamily F Member 1; AT-Rich Interactive Domain-Containing Protein 1A; AT Rich Interactive Domain 1A (SWI-Like); ARID Domain-Containing Protein 1A; SWI/SNF Complex Protein P270; BRG1-Associated Factor 250a; SWI-Like Protein; Osa Homolog 1; SMARCF1; C1orf4; BAF250; HOSA1; B120; OSA1; HELD; SWI/SNF Related, Matrix Associated, Actin Dependent Regulator Of Chromatin, Subfamily F, Member 1; AT Rich Interactive Domain 1A (SWI- Like); Chromatin Remodeling Factor P250; BRG1-Associated Factor 250; OSA1 Nuclear Protein; Brain Protein 120; BAF250a; BAF250A; BM029; MRD14; CSS2; P270; ELD | This gene encodes a member of the SWI/SNF family, whose members have helicase and ATPase activities and are thought to regulate transcription of certain genes by altering the chromatin structure around those genes. The encoded protein is part of the large ATP-dependent chromatin remodeling complex SNF/SWI, which is required for transcriptional activation of genes normally repressed by chromatin. It possesses at least two conserved domains that could be important for its function. First, it has a DNA-binding domain that can specifically bind an AT-rich DNA sequence known to be recognized by a SNF/SWI complex at the beta-globin locus. Second, the C-terminus of the protein can stimulate glucocorticoid receptor-dependent transcriptional activation. It is thought that the protein encoded by this gene confers specificity to the SNF/SWI complex and may recruit the complex to its targets through either protein-DNA or protein-protein interactions. Two transcript variants encoding different isoforms have been found for this gene. |
| *ARID2* | AT-Rich Interaction Domain 2 | AT-Rich Interactive Domain-Containing Protein 2; Zinc Finger Protein With Activation Potential; AT Rich Interactive Domain 2 (ARID, RFX-Like); ARID Domain-Containing Protein 2; BRG1-Associated Factor 200; Zipzap/P200; BAF200; KIAA1557; CSS6; P200 | This gene encodes a member of the AT-rich interactive domain (ARID)-containing family of DNA-binding proteins. Members of the ARID family have roles in embryonic patterning, cell lineage gene regulation, cell cycle control, transcriptional regulation and chromatin structure modification. This protein functions as a subunit of the polybromo- and BRG1-associated factor or PBAF (SWI/SNF-B) chromatin remodeling complex which facilitates ligand-dependent transcriptional activation by nuclear receptors. Mutations in this gene are associated with hepatocellular carcinomas. A pseudogene of this gene is found on chromosome1. |
| *ASXL1* | ASXL Transcriptional Regulator 1 | Additional Sex Combs Like 1, Transcriptional Regulator; Putative Polycomb Group Protein ASXL1; Additional Sex Combs Like Transcriptional Regulator 1; Additional Sex Combs Like 1 (Drosophila); Additional Sex Combs-Like Protein 1; KIAA0978; BOPS; MDS | This gene is similar to the Drosophila additional sex combs gene, which encodes a chromatin-binding protein required for normal determination of segment identity in the developing embryo. The protein is a member of the Polycomb group of proteins, which are necessary for the maintenance of stable repression of homeotic and other loci. The protein is thought to disrupt chromatin in localized areas, enhancing transcription of certain genes while repressing the transcription of other genes. The protein encoded by this gene functions as a ligand-dependent co-activator for retinoic acid receptor in cooperation with nuclear receptor coactivator 1. Mutations in this gene are associated with myelodysplastic syndromes and chronic myelomonocytic leukemia. Alternative splicing results in multiple transcript variants. |
| *ATM* | ATM Serine/Threonine Kinase | Ataxia Telangiectasia Mutated; Serine-Protein Kinase ATM; A-T Mutated; EC 2.7.11.1; Ataxia Telangiectasia Mutated (Includes Complementation Groups A, C And D); TEL1, Telomere Maintenance 1, Homolog (S. Cerevisiae); TEL1, Telomere Maintenance 1, Homolog; AT Mutated; TELO1; TEL1; ATDC; AT1; ATE; ATA; ATC; ATD | The protein encoded by this gene belongs to the PI3/PI4-kinase family. This protein is an important cell cycle checkpoint kinase that phosphorylates; thus, it functions as a regulator of a wide variety of downstream proteins, including tumor suppressor proteins p53 and BRCA1, checkpoint kinase CHK2, checkpoint proteins RAD17 and RAD9, and DNA repair protein NBS1. This protein and the closely related kinase ATR are thought to be master controllers of cell cycle checkpoint signaling pathways that are required for cell response to DNA damage and for genome stability. Mutations in this gene are associated with ataxia telangiectasia, an autosomal recessive disorder. |
| *AXIN* | Axin 1 | Protein Phosphatase 1, Regulatory Subunit 49; Axis Inhibition Protein 1; Axin-1; AXIN; Fused, Mouse, Homolog Of; Axis Inhibitor 1; PPP1R49; HAxin | This gene encodes a cytoplasmic protein which contains a regulation of G-protein signaling (RGS) domain and a dishevelled and axin (DIX) domain. The encoded protein interacts with adenomatosis polyposis coli, catenin beta-1, glycogen synthase kinase 3 beta, protein phosphate 2, and itself. This protein functions as a negative regulator of the wingless-type MMTV integration site family, member 1 (WNT) signaling pathway and can induce apoptosis. The crystal structure of a portion of this protein, alone and in a complex with other proteins, has been resolved. Mutations in this gene have been associated with hepatocellular carcinoma, hepatoblastomas, ovarian endometriod adenocarcinomas, and medullablastomas. Alternative splicing results in multiple transcript variants. |
| *B2M* | Beta-2-Microglobulin | Beta Chain Of MHC Class I Molecules; Beta-2-Microglobin; IMD43 | This gene encodes a serum protein found in association with the major histocompatibility complex (MHC) class I heavy chain on the surface of nearly all nucleated cells. The protein has a predominantly beta-pleated sheet structure that can form amyloid fibrils in some pathological conditions. The encoded antimicrobial protein displays antibacterial activity in amniotic fluid. A mutation in this gene has been shown to result in hypercatabolic hypoproteinemia. |
| *BCL2* | BCL2, Apoptosis Regulator | Protein Phosphatase 1, Regulatory Subunit 50; Apoptosis Regulator Bcl-2; B-Cell CLL/Lymphoma 2; PPP1R50; Bcl-2 | This gene encodes an integral outer mitochondrial membrane protein that blocks the apoptotic death of some cells such as lymphocytes. Constitutive expression of *BCL2*, such as in the case of translocation of *BCL2* to Ig heavy chain locus, is thought to be the cause of follicular lymphoma. Alternative splicing results in multiple transcript variants. |
| *BRAF* | B-Raf Proto-Oncogene, Serine/Threonine Kinase | V-Raf Murine Sarcoma Viral Oncogene Homolog B1; V-Raf Murine Sarcoma Viral Oncogene Homolog B; Serine/Threonine-Protein Kinase B-Raf; Proto-Oncogene B-Raf; BRAF1; RAFB1; B-Raf Proto-Oncogene Serine/Threonine-Protein Kinase (P94); Murine Sarcoma Viral (V-Raf) Oncogene Homolog B1; B-Raf Serine/Threonine-Protein; 94 KDa B-Raf Protein; EC 2.7.11.1; B-RAF1; B-Raf; NS7; P94 | This gene encodes a protein belonging to the RAF family of serine/threonine protein kinases. This protein plays a role in regulating the MAP kinase/ERK signaling pathway, which affects cell division, differentiation, and secretion. Mutations in this gene, most commonly the V600E mutation, are the most frequently identified cancer-causing mutations in melanoma, and have been identified in various other cancers as well, including non-Hodgkin lymphoma, colorectal cancer, thyroid carcinoma, non-small cell lung carcinoma, hairy cell leukemia and adenocarcinoma of lung. Mutations in this gene are also associated with cardiofaciocutaneous, Noonan, and Costello syndromes, which exhibit overlapping phenotypes. A pseudogene of this gene has been identified on the X chromosome. |
| *BRCA2* | BRCA2, DNA Repair Associated | Breast Cancer Type 2 Susceptibility Protein; BRCA1/BRCA2-Containing Complex, Subunit 2; Fanconi Anemia Group D1 Protein; Breast Cancer 2, Early Onset; FANCD1; FACD; Breast And Ovarian Cancer Susceptibility Gene, Early Onset; Breast And Ovarian Cancer Susceptibility Protein 2; Fanconi Anemia, Complementation Group D1; Breast Cancer 2 Tumor Suppressor; Truncated Breast Cancer 2; Breast Cancer 2; Mutant BRCA2; BROVCA2; XRCC11; BRCC2; PNCA2; FANCD; FAD1; GLM3; FAD | Inherited mutations in *BRCA1* and this gene, *BRCA2*, confer increased lifetime risk of developing breast or ovarian cancer. Both BRCA1 and BRCA2 are involved in maintenance of genome stability, specifically the homologous recombination pathway for double-strand DNA repair. The BRCA2 protein contains several copies of a 70-aa motif called the BRC motif, and these motifs mediate binding to the RAD51 recombinase which functions in DNA repair. *BRCA2* is considered a tumor suppressor gene, as tumors with *BRCA2* mutations generally exhibit loss of heterozygosity (LOH) of the wild-type allele. |
| *BTG1* | BTG Anti-Proliferation Factor 1 | B-Cell Translocation Gene 1, Anti-Proliferative; B-Cell Translocation Gene 1 Protein; Protein BTG1; APRO2 | This gene is a member of an anti-proliferative gene family that regulates cell growth and differentiation. Expression of this gene is highest in the G0/G1 phases of the cell cycle and downregulated when cells progressed through G1. The encoded protein interacts with several nuclear receptors, and functions as a coactivator of cell differentiation. This locus has been shown to be involved in a t(8;12)(q24;q22) chromosomal translocation in a case of B-cell chronic lymphocytic leukemia. |
| *CARD11* | Caspase Recruitment Domain Family Member 11 | Caspase Recruitment Domain-Containing Protein 11; Bcl10-Interacting Maguk Protein 3; CARD-Containing MAGUK Protein 1; Carma 1; CARMA1; Card-Maguk Protein 1; IMD11A; BENTA; BIMP3; IMD11; PPBL | The protein encoded by this gene belongs to the membrane-associated guanylate kinase (MAGUK) family, a class of proteins that functions as molecular scaffolds for the assembly of multiprotein complexes at specialized regions of the plasma membrane. This protein is also a member of the CARD protein family, which is defined by carrying a characteristic caspase-associated recruitment domain (CARD). This protein has a domain structure similar to that of CARD14 protein. The CARD domains of both proteins have been shown to specifically interact with BCL10, a protein known to function as a positive regulator of cell apoptosis and NF-kappaB activation. When expressed in cells, this protein activated NF-kappaB and induced the phosphorylation of BCL10. |
| *CD79B* | CD79b Molecule | B-Cell Antigen Receptor Complex-Associated Protein Beta Chain; CD79b Molecule, Immunoglobulin-Associated Beta; Immunoglobulin-Associated B29 Protein; B-Cell-Specific Glycoprotein B29; Ig-Beta; IGB; B29; CD79b Antigen (Immunoglobulin-Associated Beta); CD79B Antigen (Immunoglobulin-Associated Beta); CD79b Antigen; AGM6 | The B lymphocyte antigen receptor is a multimeric complex that includes the antigen-specific component, surface immunoglobulin (Ig). Surface Ig non-covalently associates with two other proteins, Ig-alpha and Ig-beta, which are necessary for expression and function of the B-cell antigen receptor. This gene encodes the Ig-beta protein of the B-cell antigen component. Alternatively, spliced transcript variants encoding different isoforms have been described. |
| *CDH1* | Cadherin 1 | Cadherin 1, Type 1, E-Cadherin (Epithelial); Epithelial Cadherin; Cadherin-1; Uvomorulin; E-Cadherin; CAM 120/80; CDHE; UVO; Calcium-Dependent Adhesion Protein, Epithelial; Epididymis Secretory Sperm Binding Protein; Cadherin 1, E-Cadherin (Epithelial); Cell-CAM 120/80; CD324 Antigen; E-Cadherin 1; Arc-1; BCDS1; CD324; ECAD; LCAM | This gene encodes a classical cadherin of the cadherin superfamily. Alternative splicing results in multiple transcript variants, at least one of which encodes a preproprotein that is proteolytically processed to generate the mature glycoprotein. This calcium-dependent cell-cell adhesion protein is comprised of five extracellular cadherin repeats, a transmembrane region and a highly conserved cytoplasmic tail. Mutations in this gene are correlated with gastric, breast, colorectal, thyroid and ovarian cancer. Loss of function of this gene is thought to contribute to cancer progression by increasing proliferation, invasion, and/or metastasis. The ectodomain of this protein mediates bacterial adhesion to mammalian cells and the cytoplasmic domain is required for internalization. This gene is present in a gene cluster with other members of the cadherin family on chromosome 16. |
| *CDK12*^2^ | Cyclin Dependent Kinase 12 | Cdc2-Related Kinase, Arginine/Serine-Rich; Cell Division Cycle 2-Related Protein Kinase 7; Cell Division Protein Kinase 12; CDC2-Related Protein Kinase 7; Cyclin-Dependent Kinase 12; EC 2.7.11.22; CRKRS; CRK7; CDC2 Related Protein Kinase 7; EC 2.7.11.23; EC 2.7.11; KIAA0904; HCDK12; CrkRS; CRKR | CDK12 forms protein complexes with cyclin K (CCNK). The CDK12-CCNK complex acts at the level of RNA polymerase II (RNAPII) to regulate expression of long complex genes, several of which have roles in DNA maintenance and repair. CDK12 forms protein complexes with cyclin K (CCNK). The CDK12-CCNK complex acts at the level of RNA polymerase II to regulate expression of long complex genes, several of which have roles in DNA maintenance and repair. |
| *CDKN2A* | Cyclin Dependent Kinase Inhibitor 2A | Cyclin-Dependent Kinase Inhibitor 2A (Melanoma, P16, Inhibits CDK4); Cyclin-Dependent Kinase 4 Inhibitor A; Cyclin-Dependent Kinase Inhibitor 2A; Multiple Tumor Suppressor 1; Alternative Reading Frame; P16INK4A; P14ARF; CDKN2; CDK4I; MTS-1; MTS1; MLM; ARF; Cell Cycle Negative Regulator Beta; CDK4 Inhibitor P16-INK4; Tumor Suppressor ARF; P16-INK4A; P16-INK4a; P16-INK4; P16INK4;  P19ARF; INK4A; CMM2; INK4; TP16; P14; P16; P19 | This gene generates several transcript variants which differ in their first exons. At least three alternatively spliced variants encoding distinct proteins have been reported, two of which encode structurally related isoforms known to function as inhibitors of CDK4 kinase. The remaining transcript includes an alternate first exon located 20 Kb upstream of the remainder of the gene; this transcript contains an alternate open reading frame (ARF) that specifies a protein which is structurally unrelated to the products of the other variants. This ARF product functions as a stabilizer of the tumor suppressor protein p53 as it can interact with, and sequester, the E3 ubiquitin-protein ligase MDM2, a protein responsible for the degradation of p53. In spite of the structural and functional differences, the CDK inhibitor isoforms and the ARF product encoded by this gene, through the regulatory roles of CDK4 and p53 in cell cycle G1 progression, share a common functionality in cell cycle G1 control. This gene is frequently mutated or deleted in a wide variety of tumors and is known to be an important tumor suppressor gene. |
| *CEBPA* | CCAAT Enhancer Binding Protein Alpha | CCAAT/Enhancer Binding Protein (C/EBP), Alpha; CCAAT/Enhancer-Binding Protein Alpha; CEBP; CCAAT/Enhancer Binding Protein Alpha; C/EBP-Alpha; C/EBP Alpha | This intronless gene encodes a transcription factor that contains a basic leucine zipper (bZIP) domain and recognizes the CCAAT motif in the promoters of target genes. The encoded protein functions in homodimers and also heterodimers with CCAAT/enhancer-binding proteins beta and gamma. Activity of this protein can modulate the expression of genes involved in cell cycle regulation as well as in body weight homeostasis. Mutation of this gene is associated with acute myeloid leukemia. The use of alternative in-frame non-AUG (GUG) and AUG start codons results in protein isoforms with different lengths. Differential translation initiation is mediated by an out-of-frame, upstream open reading frame which is located between the GUG and the first AUG start codons. |
| *CREBBP* | CREB Binding Protein | CREB-Binding Protein; EC 2.3.1.48; CBP; Rubinstein-Taybi Syndrome; KAT3A; RSTS1; RSTS | This gene is ubiquitously expressed and is involved in the transcriptional coactivation of many different transcription factors. First isolated as a nuclear protein that binds to cAMP-response element binding protein (CREB), this gene is now known to play critical roles in embryonic development, growth control, and homeostasis by coupling chromatin remodeling to transcription factor recognition. The protein encoded by this gene has intrinsic histone acetyltransferase activity and also acts as a scaffold to stabilize additional protein interactions with the transcription complex. This protein acetylates both histone and non-histone proteins. This protein shares regions of very high sequence similarity with protein p300 in its bromodomain, cysteine-histidine-rich regions, and histone acetyltransferase domain. Mutations in this gene cause Rubinstein-Taybi syndrome (RTS). Chromosomal translocations involving this gene have been associated with acute myeloid leukemia. Alternative splicing results in multiple transcript variants encoding different isoforms. |
| *CTNNB1* | Catenin Beta 1 | Catenin (Cadherin-Associated Protein), Beta 1, 88kDa; Catenin Beta-1; CTNNB; Catenin (Cadherin-Associated Protein), Beta 1 (88kD); Catenin (Cadherin-Associated Protein), Beta 1; Beta-Catenin; Armadillo; MRD19; EVR7 | The protein encoded by this gene is part of a complex of proteins that constitute adherens junctions (AJs). AJs are necessary for the creation and maintenance of epithelial cell layers by regulating cell growth and adhesion between cells. The encoded protein also anchors the actin cytoskeleton and may be responsible for transmitting the contact inhibition signal that causes cells to stop dividing once the epithelial sheet is complete. Finally, this protein binds to the product of the APC gene, which is mutated in adenomatous polyposis of the colon. Mutations in this gene are a cause of colorectal cancer (CRC), pilomatrixoma (PTR), medulloblastoma (MDB), and ovarian cancer. Alternative splicing results in multiple transcript variants. |
| *DNM2* | Dynamin 2 | Dynamin II; EC 3.6.5.5; Dynamin-2; DYN2; Cytoskeletal Protein; DI-CMTB; CMTDI1; CMTDIB; CMT2M; DYNII; LCCS5 | Dynamins represent one of the subfamilies of GTP-binding proteins. These proteins share considerable sequence similarity over the N-terminal portion of the molecule, which contains the GTPase domain. Dynamins are associated with microtubules. They have been implicated in cell processes such as endocytosis and cell motility, and in alterations of the membrane that accompany certain activities such as bone resorption by osteoclasts. Dynamins bind many proteins that bind actin and other cytoskeletal proteins. Dynamins can also self-assemble, a process that stimulates GTPase activity. Five alternatively spliced transcripts encoding different proteins have been described. Additional alternatively spliced transcripts may exist, but their full-length nature has not been determined. |
| *DNMT3A* | DNA Methyltransferase 3 Alpha | DNA (Cytosine-5-)-Methyltransferase 3 Alpha; DNA (Cytosine-5)-Methyltransferase 3A; DNA MTase HsaIIIA; EC 2.1.1.37; M.HsaIIIA; DNA Cytosine Methyltransferase 3A2; DNA Methyltransferase HsaIIIA; DNMT3A2; Dnmt3a; TBRS | CpG methylation is an epigenetic modification that is important for embryonic development, imprinting, and X-chromosome inactivation. Studies in mice have demonstrated that DNA methylation is required for mammalian development. This gene encodes a DNA methyltransferase that is thought to function in de novo methylation, rather than maintenance methylation. The protein localizes to the cytoplasm and nucleus and its expression is developmentally regulated. |
| *EGFR* | Epidermal Growth Factor Receptor | Receptor Tyrosine-Protein Kinase ErbB-1; Erb-B2 Receptor Tyrosine Kinase 1; Proto-Oncogene C-ErbB-1; EC 2.7.10.1; ERBB1; ERBB; HER1; Epidermal Growth Factor Receptor (Avian Erythroblastic Leukemia Viral (V-Erb-B) Oncogene Homolog); Erythroblastic Leukemia Viral (V-Erb-B) Oncogene Homolog (Avian); Avian Erythroblastic Leukemia Viral (V-Erb-B) Oncogene Homolog; Epidermal Growth Factor Receptor Tyrosine Kinase Domain; Cell Proliferation-Inducing Protein 61; Cell Growth Inhibiting Protein 40; EC 2.7.10; NISBD2; PIG61; MENA | The protein encoded by this gene is a transmembrane glycoprotein that is a member of the protein kinase superfamily. This protein is a receptor for members of the epidermal growth factor family. EGFR is a cell surface protein that binds to epidermal growth factor. Binding of the protein to a ligand induces receptor dimerization and tyrosine autophosphorylation and leads to cell proliferation. Mutations in this gene are associated with lung cancer. |
| *EP300* | E1A Binding Protein P300 | Histone Acetyltransferase P300 Protein Propionyltransferase P300; Histone Crotonyltransferase P300; Histone Butyryltransferase P300; E1A-Associated Protein P300; EC 2.3.1.48; P300 HAT; P300; E1A-Binding Protein, 300kD; EC 2.3.1.-; EC 2.3.1; KAT3B; RSTS2 | This gene encodes the adenovirus E1A-associated cellular p300 transcriptional co-activator protein. It functions as histone acetyltransferase that regulates transcription via chromatin remodeling and is important in the processes of cell proliferation and differentiation. It mediates cAMP-gene regulation by binding specifically to phosphorylated CREB protein. This gene has also been identified as a co-activator of HIF1A (hypoxia-inducible factor 1 alpha), and thus plays a role in the stimulation of hypoxia-induced genes such as *VEGF*. Defects in this gene are a cause of Rubinstein-Taybi syndrome and may also play a role in epithelial cancer. |
| *ERBB2* | Erb-B2 Receptor Tyrosine Kinase 2 | V-Erb-B2 Avian Erythroblastic Leukemia Viral Oncogene Homolog 2; Tyrosine Kinase-Type Cell Surface Receptor HER2; Neuro/Glioblastoma Derived Oncogene Homolog; Human Epidermal Growth Factor Receptor 2; Receptor Tyrosine-Protein Kinase ErbB-2; Metastatic Lymph Node Gene 19 Protein; Proto-Oncogene C-ErbB-2; Proto-Oncogene Neu; EC 2.7.10.1; P185erbB2; MLN 19; HER2; NGL; NEU; V-Erb-B2 Avian Erythroblastic Leukemia Viral Oncogene Homolog 2 (Neuro/Glioblastoma Derived Oncogene Homolog); V-Erb-B2 Erythroblastic Leukemia Viral Oncogene Homolog 2, Neuro/Glioblastoma Derived Oncogene Homolog; V-Erb-B2 Avian Erythroblastic Leukemia Viral Oncoprotein 2; Neuroblastoma/Glioblastoma Derived Oncogene Homolog; C-Erb B2/Neu Protein; CD340 Antigen; HER-2/Neu; Herstatin; EC 2.7.10; CD340; HER-2; MLN19; TKR1 | This gene encodes a member of the epidermal growth factor (EGF) receptor family of receptor tyrosine kinases. This protein has no ligand binding domain of its own and therefore cannot bind growth factors. However, it does bind tightly to other ligand-bound EGF receptor family members to form a heterodimer, stabilizing ligand binding and enhancing kinase-mediated activation of downstream signaling pathways, such as those involving mitogen-activated protein kinase and phosphatidylinositol-3 kinase. Allelic variations at amino acid positions 654 and 655 of isoform a (positions 624 and 625 of isoform b) have been reported, with the most common allele, Ile654/Ile655, shown here. Amplification and/or overexpression of this gene has been reported in numerous cancers, including breast and ovarian tumors. Alternative splicing results in several additional transcript variants, some encoding different isoforms and others that have not been fully characterized. |
| *ERBB3* | Erb-B2 Receptor Tyrosine Kinase 3 | V-Erb-B2 Avian Erythroblastic Leukemia Viral Oncogene Homolog 3; Tyrosine Kinase-Type Cell Surface Receptor HER3; Human Epidermal Growth Factor Receptor 3; Receptor Tyrosine-Protein Kinase ErbB-3; Proto-Oncogene-Like Protein C-ErbB-3; EC 2.7.10.1; HER3; Lethal Congenital Contracture Syndrome 2; P180-ErbB3; P45-SErbB3; P85-SErbB3; EC 2.7.10; MDA-BF-1; C-ErbB-3; C-ErbB3; ErbB3-S; ErbB-3; LCCS2 | This gene encodes a member of the epidermal growth factor receptor (EGFR) family of receptor tyrosine kinases. This membrane-bound protein has a neuregulin binding domain but not an active kinase domain. It therefore can bind this ligand but not convey the signal into the cell through protein phosphorylation. However, it does form heterodimers with other EGF receptor family members which do have kinase activity. Heterodimerization leads to the activation of pathways which lead to cell proliferation or differentiation. Amplification of this gene and/or overexpression of its protein have been reported in numerous cancers, including prostate, bladder, and breast tumors. Alternate transcriptional splice variants encoding different isoforms have been characterized. One isoform lacks the intermembrane region and is secreted outside the cell. This form acts to modulate the activity of the membrane-bound form. Additional splice variants have also been reported, but they have not been thoroughly characterized. |
| *ERBB4* | Erb-B2 Receptor Tyrosine Kinase 4 | V-Erb-B2 Avian Erythroblastic Leukemia Viral Oncogene Homolog 4; Tyrosine Kinase-Type Cell Surface Receptor HER4; Human Epidermal Growth Factor Receptor 4; Receptor Tyrosine-Protein Kinase ErbB-4; Proto-Oncogene-Like Protein C-ErbB-4; EC 2.7.10.1; P180erbB4; HER4; V-Erb-A Avian Erythroblastic Leukemia Viral Oncogene Homolog-Like 4; Avian Erythroblastic Leukemia Viral (V-Erb-B2) Oncogene Homolog 4; V-Erb-A Erythroblastic Leukemia Viral Oncogene Homolog 4; ERBB4 Transcript Variant I12DEL; ERBB4 Transcript Variant I20DEL; EC 2.7.10; ALS19 | This gene is a member of the Tyr protein kinase family and the epidermal growth factor receptor subfamily. It encodes a single-pass type I membrane protein with multiple cysteine rich domains, a transmembrane domain, a tyrosine kinase domain, a phosphotidylinositol-3 kinase binding site and a PDZ domain binding motif. The protein binds to and is activated by neuregulins and other factors and induces a variety of cellular responses including mitogenesis and differentiation. Multiple proteolytic events allow for the release of a cytoplasmic fragment and an extracellular fragment. Mutations in this gene have been associated with cancer. Alternatively, spliced variants which encode different protein isoforms have been described; however, not all variants have been fully characterized. |
| *ESR1* | Estrogen Receptor 1 | Nuclear Receptor Subfamily 3 Group A Member 1; Oestrogen Receptor Alpha; Estradiol Receptor; Estrogen Receptor; E2 Receptor Alpha; ER-Alpha; NR3A1; ESR; ER; Estrogen Receptor Alpha E1-N2-E2-1-2; Estrogen Receptor Alpha E1-E2-1-2; Estrogen Nuclear Receptor Alpha; Estrogen Receptor Alpha; ESTRR; ESRA; Era | This gene encodes an estrogen receptor, a ligand-activated transcription factor composed of several domains important for hormone binding, DNA binding, and activation of transcription. The protein localizes to the nucleus where it may form a homodimer or a heterodimer with estrogen receptor 2. Estrogen and its receptors are essential for sexual development and reproductive function, but also play a role in other tissues such as bone. Estrogen receptors are also involved in pathological processes including breast cancer, endometrial cancer, and osteoporosis. Alternative promoter usage and alternative splicing result in dozens of transcript variants, but the full-length nature of many of these variants has not been determined. |
| *EZH2* | Enhancer of Zeste 2 Polycomb Repressive Complex 2 Subunit | Histone-Lysine N-Methyltransferase EZH2; Lysine N-Methyltransferase 6; Enhancer of Zeste Homolog 2; EC 2.1.1.43; ENX-1; KMT6; Enhancer Of Zeste (Drosophila) Homolog 2; Enhancer Of Zeste Homolog 2 (Drosophila); EC 2.1.1.43'); EC 2.1.1; EZH2b; KMT6A; ENX1; WVS2; WVS | This gene encodes a member of the Polycomb-group (PcG) family. PcG family members form multimeric protein complexes, which are involved in maintaining the transcriptional repressive state of genes over successive cell generations. This protein associates with the embryonic ectoderm development protein, the VAV1 oncoprotein, and the X-linked nuclear protein. This protein may play a role in the hematopoietic and central nervous systems. Multiple alternatively spliced transcript variants encoding distinct isoforms have been identified for this gene. |
| *FAT1* | FAT Atypical Cadherin 1 | Cadherin-Related Tumor Suppressor Homolog; Cadherin-Related Family Member 8; Cadherin Family Member 7; Protein Fat Homolog; Protocadherin Fat 1; CDHF7; FAT; FAT Tumor Suppressor Homolog 1 (Drosophila); FAT Tumor Suppressor (Drosophila) Homolog; FAT Tumor Suppressor 1; Cadherin ME5; CDHR8; HFat1; ME5 | This gene is an ortholog of the Drosophila fat gene, which encodes a tumor suppressor essential for controlling cell proliferation during Drosophila development. The gene product is a member of the cadherin superfamily, a group of integral membrane proteins characterized by the presence of cadherin-type repeats. In addition to containing 34 tandem cadherin-type repeats, the gene product has five epidermal growth factor (EGF)-like repeats and one laminin A-G domain. This gene is expressed at high levels in a number of fetal epithelia. Its product probably functions as an adhesion molecule and/or signaling receptor and is likely to be important in developmental processes and cell communication. Transcript variants derived from alternative splicing and/or alternative promoter usage exist, but they have not been fully described. |
| *FAT4* | FAT Atypical Cadherin 4 | Cadherin-Related Family Member 11; Fat-Like Cadherin Protein FAT-J; FAT Tumor Suppressor Homolog 4; Cadherin Family Member 14; Protocadherin Fat 4; CDHF14; FATJ; FAT Tumor Suppressor Homolog 4 (Drosophila); Putative Protein Product of Nbla00548; NBLA00548; CDHR11; HKLLS2; VMLDS2; FAT-J; HFat4 | The protein encoded by this gene is a member of the protocadherin family. This gene may play a role in regulating planar cell polarity (PCP). Studies in mice suggest that loss of PCP signaling may cause cystic kidney disease, and mutations in this gene have been associated with Van Maldergem Syndrome 2. Alternatively spliced transcript variants have been noted for this gene. |
| *FBXW7* | F-Box and WD Repeat Domain Containing 7 | F-Box And WD Repeat Domain Containing 7, E3 Ubiquitin Protein Ligase; F-Box And WD-40 Domain Protein 7 (Archipelago Homolog, Drosophila); F-Box/WD Repeat-Containing Protein 7; F-Box Protein FBX30; SEL-10; FBX30; SEL10; HCdc4; FBW7 HAgo F-Box And WD-40 Domain-Containing Protein 7; Archipelago Homolog (Drosophila); Homolog Of C Elegans Sel-10; F-Box Protein SEL-10; Archipelago Homolog; F-Box Protein FBW7; Archipelago; FBXO30; FBXW6; CDC4; FBW6; AGO | This gene encodes a member of the F-box protein family which is characterized by an approximately 40 amino acid motif, the F-box. The F-box proteins constitute one of the four subunits of ubiquitin protein ligase complex called SCFs (SKP1-cullin-F-box), which function in phosphorylation-dependent ubiquitination. The F-box proteins are divided into 3 classes: Fbws containing WD-40 domains, Fbls containing leucine-rich repeats, and Fbxs containing either different protein-protein interaction modules or no recognizable motifs. The protein encoded by this gene was previously referred to as FBX30 and belongs to the Fbws class; in addition to an F-box, this protein contains 7 tandem WD40 repeats. This protein binds directly to cyclin E and probably targets cyclin E for ubiquitin-mediated degradation. Mutations in this gene are detected in ovarian and breast cancer cell lines, implicating the gene's potential role in the pathogenesis of human cancers. Multiple transcript variants encoding different isoforms have been found for this gene. |
| *FLT3* | Fms Related Tyrosine Kinase 3 | Receptor-Type Tyrosine-Protein Kinase FLT3; Stem Cell Tyrosine Kinase 1; Fms-Like Tyrosine Kinase 3; FL Cytokine Receptor; CD135 Antigen; EC 2.7.10.1; CD135; FLK-2; FLK2; STK1; Growth Factor Receptor Tyrosine Kinase Type III; Fms-Related Tyrosine Kinase 3; Fetal Liver Kinase 2; Fetal Liver Kinase-2; EC 2.7.10; FLT-3; STK-1 | This gene encodes a class III receptor tyrosine kinase that regulates hematopoiesis. This receptor is activated by binding of the fms-related tyrosine kinase 3 ligand to the extracellular domain, which induces homodimer formation in the plasma membrane leading to autophosphorylation of the receptor. The activated receptor kinase subsequently phosphorylates and activates multiple cytoplasmic effector molecules in pathways involved in apoptosis, proliferation, and differentiation of hematopoietic cells in bone marrow. Mutations that result in the constitutive activation of this receptor result in acute myeloid leukemia and acute lymphoblastic leukemia. |
| *FOXA1* | Forkhead Box A1 | Hepatocyte Nuclear Factor 3-Alpha; Forkhead Box Protein A1; Transcription Factor 3A; HNF-3-Alpha; HNF-3A; TCF-3A; HNF3A; TCF3A; Hepatocyte Nuclear Factor 3, Alpha | This gene encodes a member of the forkhead class of DNA-binding proteins. These hepatocyte nuclear factors are transcriptional activators for liver-specific transcripts such as albumin and transthyretin, and they also interact with chromatin. Similar family members in mice have roles in the regulation of metabolism and in the differentiation of the pancreas and liver. |
| *GATA3* | GATA Binding Protein 3 | Trans-Acting T-Cell-Specific Transcription Factor GATA-3; GATA-Binding Factor 3; HDRS; HDR | This gene encodes a protein which belongs to the GATA family of transcription factors. The protein contains two GATA-type zinc fingers and is an important regulator of T-cell development and plays an important role in endothelial cell biology. Defects in this gene are the cause of hypoparathyroidism with sensorineural deafness and renal dysplasia. |
| *GNAS* | GNAS Complex Locus | Guanine Nucleotide Binding Protein (G Protein), Alpha Stimulating Activity Polypeptide 1; Guanine Nucleotide-Binding Protein G(S) Subunit Alpha Isoforms XLas; Adenylate Cyclase-Stimulating G Alpha Protein; Alternative Gene Product Encoded By XL-Exon; Extra Large Alphas Protein; G Protein Subunit Alpha S; Secretogranin VI; Protein ALEX; GNAS1; GSP; Guanine Nucleotide-Binding Protein G(S) Subunit Alpha Isoforms Short; Guanine Nucleotide Regulatory Protein; Neuroendocrine Secretory Protein 55; Neuroendocrine Secretory Protein; Protein SCG6 (Secretogranin VI); Protein GNAS; C20orf45; XLalphas; NESP55; PITA3; GPSA; NESP; SCG6; SgVI; AHO; GSA; POH | This locus has a highly complex imprinted expression pattern. It gives rise to maternally, paternally, and biallelically expressed transcripts that are derived from four alternative promoters and 5' exons. Some transcripts contain a differentially methylated region (DMR) at their 5' exons, and this DMR is commonly found in imprinted genes and correlates with transcript expression. An antisense transcript is produced from an overlapping locus on the opposite strand. One of the transcripts produced from this locus, and the antisense transcript, are paternally expressed noncoding RNAs, and may regulate imprinting in this region. In addition, one of the transcripts contains a second overlapping ORF, which encodes a structurally unrelated protein - Alex. Alternative splicing of downstream exons is also observed, which results in different forms of the stimulatory G-protein alpha subunit, a key element of the classical signal transduction pathway linking receptor-ligand interactions with the activation of adenylyl cyclase and a variety of cellular responses. Multiple transcript variants encoding different isoforms have been found for this gene. Mutations in this gene result in pseudohypoparathyroidism type 1a, pseudohypoparathyroidism type 1b, Albright hereditary osteodystrophy, pseudohypoparathyroidism, McCune-Albright syndrome, progressive osseous heteroplasia, polyostotic fibrous dysplasia of bone, and some pituitary tumors. |
| *GRIN2A* | Glutamate Ionotropic Receptor NMDA Type Subunit 2A | Glutamate Receptor, Ionotropic, N-Methyl D-Aspartate 2A; N-Methyl D-Aspartate Receptor Subtype 2A; Glutamate Receptor Ionotropic, NMDA 2A; NMDAR2A; GluN2A; NR2A; N-Methyl-D-Aspartate Receptor Channel, Subunit Epsilon-1; Glutamate [NMDA] Receptor Subunit Epsilon-1; N-Methyl-D-Aspartate Receptor Subunit 2A; HNR2A; EPND; FESD; LKS | This gene encodes a member of the glutamate-gated ion channel protein family. The encoded protein is an N-methyl-D-aspartate (NMDA) receptor subunit. NMDA receptors are both ligand-gated and voltage-dependent, and are involved in long-term potentiation, an activity-dependent increase in the efficiency of synaptic transmission thought to underlie certain kinds of memory and learning. These receptors are permeable to calcium ions, and activation results in a calcium influx into post-synaptic cells, which results in the activation of several signaling cascades. Disruption of this gene is associated with focal epilepsy and speech disorder with or without cognitive disability. Alternative splicing results in multiple transcript variants. |
| *IDH1* | Isocitrate Dehydrogenase (NADP(+)) 1, Cytosolic | Isocitrate Dehydrogenase [NADP] Cytoplasmic; Isocitrate Dehydrogenase 1 (NADP+), Soluble; Oxalosuccinate Decarboxylase; NADP(+)-Specific ICDH; EC 1.1.1.42; PICD; IDH; IDP; NADP-Dependent Isocitrate Dehydrogenase, Peroxisomal; NADP-Dependent Isocitrate Dehydrogenase, Cytosolic; Epididymis Secretory Sperm Binding Protein; Cytosolic NADP-Isocitrate Dehydrogenase; Epididymis Secretory Protein Li 26; Isocitrate Dehydrogenase 1 (NADP+); Epididymis Luminal Protein 216; HEL-S-26; HEL-216; IDCD; IDPC | Isocitrate dehydrogenases catalyze the oxidative decarboxylation of isocitrate to 2-oxoglutarate. These enzymes belong to two distinct subclasses, one of which utilizes NAD(+) as the electron acceptor and the other NADP(+). Five isocitrate dehydrogenases have been reported: three NAD(+)-dependent isocitrate dehydrogenases, which localize to the mitochondrial matrix, and two NADP(+)-dependent isocitrate dehydrogenases, one of which is mitochondrial and the other predominantly cytosolic. Each NADP(+)-dependent isozyme is a homodimer. The protein encoded by this gene is the NADP(+)-dependent isocitrate dehydrogenase found in the cytoplasm and peroxisomes. It contains the PTS-1 peroxisomal targeting signal sequence. The presence of this enzyme in peroxisomes suggests roles in the regeneration of NADPH for intraperoxisomal reductions, such as the conversion of 2, 4-dienoyl-CoAs to 3-enoyl-CoAs, as well as in peroxisomal reactions that consume 2-oxoglutarate, namely the alpha-hydroxylation of phytanic acid. The cytoplasmic enzyme serves a significant role in cytoplasmic NADPH production. Alternatively spliced transcript variants encoding the same protein have been found for this gene. |
| *IDH2* | Isocitrate Dehydrogenase (NADP(+)) 2, Mitochondrial | Isocitrate Dehydrogenase 2 (NADP+), Mitochondrial; Isocitrate Dehydrogenase [NADP], Mitochondrial; Oxalosuccinate Decarboxylase; NADP(+)-Specific ICDH; EC 1.1.1.42; ICD-M; IDH; IDP; MNADP-IDH; D2HGA2; IDHM; IDPM | Isocitrate dehydrogenases catalyze the oxidative decarboxylation of isocitrate to 2-oxoglutarate. These enzymes belong to two distinct subclasses, one of which utilizes NAD(+) as the electron acceptor and the other NADP(+). Five isocitrate dehydrogenases have been reported: three NAD(+)-dependent isocitrate dehydrogenases, which localize to the mitochondrial matrix, and two NADP(+)-dependent isocitrate dehydrogenases, one of which is mitochondrial and the other predominantly cytosolic. Each NADP(+)-dependent isozyme is a homodimer. The protein encoded by this gene is the NADP(+)-dependent isocitrate dehydrogenase found in the mitochondria. It plays a role in intermediary metabolism and energy production. This protein may tightly associate or interact with the pyruvate dehydrogenase complex. Alternative splicing results in multiple transcript variants. |
| *KEAP1* | Kelch Like ECH Associated Protein 1 | Kelch-Like ECH-Associated Protein 1; Cytosolic Inhibitor Of Nrf2; Kelch-Like Family Member 19; Kelch-Like Protein 19; KLHL19; INrf2; KEAP1 Delta C; KIAA0132; INRF2 | This gene encodes a protein containing KELCH-1 like domains, as well as a BTB/POZ domain. Kelch-like ECH-associated protein 1 interacts with NF-E2-related factor 2 in a redox-sensitive manner and the dissociation of the proteins in the cytoplasm is followed by transportation of NF-E2-related factor 2 to the nucleus. This interaction results in the expression of the catalytic subunit of gamma-glutamylcysteine synthetase. Two alternatively spliced transcript variants encoding the same isoform have been found for this gene. |
| *KIT* | KIT Proto-Oncogene Receptor Tyrosine Kinase | KIT Proto-Oncogene Receptor Tyrosine Kinase; V-Kit Hardy-Zuckerman 4 Feline Sarcoma Viral Oncogene Homolog; Mast/Stem Cell Growth Factor Receptor Kit; Tyrosine-Protein Kinase Kit; Piebald Trait Protein; Proto-Oncogene C-Kit; EC 2.7.10.1; P145 C-Kit; SCFR; PBT; V-Kit Hardy-Zuckerman 4 Feline Sarcoma Viral Oncogene-Like Protein; Proto-Oncogene Tyrosine-Protein Kinase Kit; Soluble KIT Variant 1; C-Kit Protooncogene; CD117 Antigen; Piebald Trait; EC 2.7.10; C-Kit; CD117; MASTC | This gene encodes the human homolog of the proto-oncogene c-kit. C-kit was first identified as the cellular homolog of the feline sarcoma viral oncogene v-kit. This protein is a type 3 transmembrane receptor for MGF (mast cell growth factor, also known as stem cell factor). Mutations in this gene are associated with gastrointestinal stromal tumors, mast cell disease, acute myelogenous leukemia, and piebaldism. Multiple transcript variants encoding different isoforms have been found for this gene. |
| *KMT2A* | Lysine Methyltransferase 2A | Histone-Lysine N-Methyltransferase 2A; Myeloid/Lymphoid Or Mixed-Lineage Leukemia (Trithorax Homolog, Drosophila); Lysine (K)-Specific Methyltransferase 2A; CXXC-Type Zinc Finger Protein 7; Lysine N-Methyltransferase 2A; Zinc Finger Protein HRX; Trithorax-Like Protein; ALL-1; CXXC7; MLL1; TRX1; MLL; HRX; Myeloid/Lymphoid Or Mixed-Lineage Leukemia (Trithorax (Drosophila) Homolog); Myeloid/Lymphoid Or Mixed-Lineage Leukemia Protein 1; Myeloid/Lymphoid Or Mixed-Lineage Leukemia; Mixed Lineage Leukemia 1; EC 2.1.1.43; HTRX1; MLL1A; WDSTS; ALL1; HTRX | This gene encodes a transcriptional coactivator that plays an essential role in regulating gene expression during early development and hematopoiesis. The encoded protein contains multiple conserved functional domains. One of these domains, the SET domain, is responsible for its histone H3 lysine 4 (H3K4) methyltransferase activity which mediates chromatin modifications associated with epigenetic transcriptional activation. This protein is processed by the enzyme Taspase 1 into two fragments, MLL-C and MLL-N. These fragments re-associate and further assemble into different multiprotein complexes that regulate the transcription of specific target genes, including many of the HOX genes. Multiple chromosomal translocations involving this gene are the cause of certain acute lymphoid leukemias and acute myeloid leukemias. Alternate splicing results in multiple transcript variants. |
| *KMT2C* | Lysine Methyltransferase 2C | Histone-Lysine N-Methyltransferase 2C; Myeloid/Lymphoid Or Mixed-Lineage Leukemia Protein 3; Lysine (K)-Specific Methyltransferase 2C; Homologous To ALR Protein; MLL3; HALR; Histone-Lysine N-Methyltransferase, H3 Lysine-4 Specific; Myeloid/Lymphoid Or Mixed-Lineage Leukemia 3; Histone-Lysine N-Methyltransferase MLL3; Lysine N-Methyltransferase 2C; ALR-Like Protein; EC 2.1.1.43; KIAA1506; KLEFS2 | This gene is a member of the myeloid/lymphoid or mixed-lineage leukemia (MLL) family and encodes a nuclear protein with an AT hook DNA-binding domain, a DHHC-type zinc finger, six PHD-type zinc fingers, a SET domain, a post-SET domain and a RING-type zinc finger. This protein is a member of the ASC-2/NCOA6 complex (ASCOM), which possesses histone methylation activity and is involved in transcriptional coactivation. |
| *KMT2D* | Lysine Methyltransferase 2D | Histone-Lysine N-Methyltransferase 2D; Myeloid/Lymphoid Or Mixed-Lineage Leukemia 2; Lysine (K)-Specific Methyltransferase 2D; Trinucleotide Repeat Containing 21; Lysine N-Methyltransferase 2D; ALL1-Related Protein; MLL2; MLL4; ALR; Myeloid/Lymphoid Or Mixed-Lineage Leukemia Protein 2; Histone-Lysine N-Methyltransferase MLL2; Truncated Lysine Methyltransferase 2D; Kabuki Mental Retardation Syndrome; Kabuki Make-Up Syndrome; EC 2.1.1.43; CAGL114; KABUK1; TNRC21; AAD10; KMS | The protein encoded by this gene is a histone methyltransferase that methylates the Lys-4 position of histone H3. The encoded protein is part of a large protein complex called ASCOM, which has been shown to be a transcriptional regulator of the beta-globin and estrogen receptor genes. Mutations in this gene have been shown to be a cause of Kabuki syndrome. |
| *KRAS* | KRAS Proto-Oncogene, GTPase | V-Ki-Ras2 Kirsten Rat Sarcoma 2 Viral Oncogene Homolog; Kirsten Rat Sarcoma Viral Oncogene Homolog; GTPase KRas; C-Ki-Ras; K-Ras 2; KRAS2; RASK2; V-Ki-Ras2 Kirsten Rat Sarcoma Viral Oncogene Homolog; Kirsten Rat Sarcoma Viral Proto-Oncogene; Cellular Transforming Proto-Oncogene; Cellular C-Ki-Ras2 Proto-Oncogene; Transforming Protein P21; PR310 C-K-Ras Oncogene; C-Kirsten-Ras Protein; K-Ras P21 Protein; Oncogene KRAS2; C-Ki-Ras2; C-K-RAS; K-RAS2A; K-RAS2B; K-RAS4A; K-RAS4B; C-K-Ras; KI-RAS; Ki-Ras; K-Ras; KRAS1; CFC2; RALD; NS3; NS | This gene, a Kirsten *ras* oncogene homolog from the mammalian *ras* gene family, encodes a protein that is a member of the small GTPase superfamily. A single amino acid substitution is responsible for an activating mutation. The transforming protein that results is implicated in various malignancies, including lung adenocarcinoma, mucinous adenoma, ductal carcinoma of the pancreas and colorectal carcinoma. Alternative splicing leads to variants encoding two isoforms that differ in the C-terminal region. |
| *LRP1B* | LDL Receptor Related Protein 1B | Low-Density Lipoprotein Receptor-Related Protein 1B; Low Density Lipoprotein Receptor-Related Protein 1B; LRP-Deleted In Tumors; LRP-DIT; LRP-1B; LRPDIT; Low Density Lipoprotein Receptor Related Protein-Deleted In Tumor; Low-Density Lipoprotein Receptor-Related Protein-Deleted In Tumor; Low Density Lipoprotein-Related Protein 1B (Deleted In Tumors); EC 3.4.21.9; EC 1.1.1.94 | This gene encodes a member of the low density lipoprotein (LDL) receptor family. These receptors play a wide variety of roles in normal cell function and development due to their interactions with multiple ligands. Disruption of this gene has been reported in several types of cancer. |
| *MAP2K2* | Mitogen-Activated Protein Kinase Kinase 2 | Dual Specificity Mitogen-Activated Protein Kinase Kinase 2; ERK Activator Kinase 2; MAP Kinase Kinase 2; MAPK/ERK Kinase 2; EC 2.7.12.2; PRKMK2; MEK2; MKK2; Mitogen-Activated Protein Kinase Kinase 2, P45; MAPKK 2; MAPKK2; MEK 2; CFC4 | The protein encoded by this gene is a dual specificity protein kinase that belongs to the MAP kinase kinase family. This kinase is known to play a critical role in mitogen growth factor signal transduction. It phosphorylates and thus activates MAPK1/ERK2 and MAPK2/ERK3. The activation of this kinase itself is dependent on the Ser/Thr phosphorylation by MAP kinase kinase kinases. Mutations in this gene cause cardiofaciocutaneous syndrome (CFC syndrome), a disease characterized by heart defects, cognitive disability, and distinctive facial features similar to those found in Noonan syndrome. The inhibition or degradation of this kinase is also found to be involved in the pathogenesis of Yersinia and anthrax. A pseudogene, which is located on chromosome 7, has been identified for this gene. |
| *MAP2K4* | Mitogen-Activated Protein Kinase Kinase 4 | Dual Specificity Mitogen-Activated Protein Kinase Kinase 4; Stress-Activated Protein Kinase Kinase 1; C-Jun N-Terminal Kinase Kinase 1; JNK-Activating Kinase 1; MAP Kinase Kinase 4; MAPK/ERK Kinase 4; SAPK/ERK Kinase 1; EC 2.7.12.2; MAPKK 4; SAPKK-1; PRKMK4; SAPKK1; SERK1; JNKK1; MEK 4; JNKK; MEK4; MKK4; SEK1; SKK1; JNK-Activated Kinase 1; SAPK Kinase 1; MAPKK4 | This gene encodes a member of the mitogen-activated protein kinase (MAPK) family. Members of this family act as an integration point for multiple biochemical signals and are involved in a wide variety of cellular processes such as proliferation, differentiation, transcription regulation, and development. They form a three-tiered signaling module composed of MAPKKKs, MAPKKs, and MAPKs. This protein is phosphorylated at serine and threonine residues by MAPKKKs and subsequently phosphorylates downstream MAPK targets at threonine and tyrosine residues. A similar protein in mouse has been reported to play a role in liver organogenesis. A pseudogene of this gene is located on the long arm of chromosome X. Alternative splicing results in multiple transcript variants. |
| *MYD88* | MYD88, Innate Immune Signal Transduction Adaptor | Myeloid Differentiation Primary Response Protein MyD88; Myeloid Differentiation Primary Response Gene (88); Myeloid Differentiation Primary Response 88; Mutant Myeloid Differentiation Primary Response 88; MYD88D | This gene encodes a cytosolic adapter protein that plays a central role in the innate and adaptive immune response. This protein functions as an essential signal transducer in the interleukin-1 and Toll-like receptor signaling pathways. These pathways regulate that activation of numerous proinflammatory genes. The encoded protein consists of an N-terminal death domain and a C-terminal Toll-interleukin1 receptor domain. Patients with defects in this gene have an increased susceptibility to pyogenic bacterial infections. Alternate splicing results in multiple transcript variants. |
| *NCOR1* | Nuclear Receptor Corepressor 1 | Thyroid Hormone- And Retinoic Acid Receptor-Associated Corepressor 1; Protein Phosphatase 1, Regulatory Subunit 109; N-CoR1; N-CoR; Nuclear Receptor Co-Repressor 1; PPP1R109; KIAA1047; HN-CoR; TRAC1 | This gene encodes a protein that mediates ligand-independent transcription repression of thyroid-hormone and retinoic-acid receptors by promoting chromatin condensation and preventing access of the transcription machinery. It is part of a complex which also includes histone deacetylases and transcriptional regulators similar to the yeast protein Sin3p. This gene is located between the Charcot-Marie-Tooth and Smith-Magenis syndrome critical regions on chromosome 17. Alternate splicing results in multiple transcript variants. Pseudogenes of this gene are found on chromosomes 17 and 20. |
| *NF1* | Neurofibromin 1 | Neurofibromatosis-Related Protein NF-1; Neurofibromin; Von Recklinghausen Disease; Truncated Neurofibromin 1; Neurofibromatosis 1; Neurofibromatosis; Watson Disease; NFNS; VRNF; WSS | This gene product appears to function as a negative regulator of the ras signal transduction pathway. Mutations in this gene have been linked to neurofibromatosis type 1, juvenile myelomonocytic leukemia and Watson syndrome. The mRNA for this gene is subject to RNA editing (CGA>UGA->Arg1306Term) resulting in premature translation termination. Alternatively spliced transcript variants encoding different isoforms have also been described for this gene. |
| *NFE2L2* | Nuclear Factor, Erythroid 2 Like 2 | Nuclear Factor Erythroid 2-Related Factor 2; NF-E2-Related Factor 2; HEBP1; NRF2; Nuclear Factor, Erythroid Derived 2, Like 2; Nuclear Factor (Erythroid-Derived 2)-Like 2; Nuclear Factor Erythroid-Derived 2-Like 2; Nuclear Factor, Erythroid 2-Like 2; NFE2-Related Factor 2; IMDDHH | This gene encodes a transcription factor which is a member of a small family of basic leucine zipper (bZIP) proteins. The encoded transcription factor regulates genes which contain antioxidant response elements (ARE) in their promoters; many of these genes encode proteins involved in response to injury and inflammation which includes the production of free radicals. Multiple transcript variants encoding different isoforms have been characterized for this gene. |
| *NOTCH1* | Notch 1 | Translocation-Associated Notch Protein TAN-1; Neurogenic Locus Notch Homolog Protein 1; TAN1; HN1; Notch (Drosophila) Homolog 1 (Translocation-Associated); Notch Homolog 1, Translocation-Associated (Drosophila); Notch Homolog 1, Translocation-Associated; EC 3.4.21.68; EC 2.1.2.11; AOVD1; AOS5 | This gene encodes a member of the NOTCH family of proteins. Members of this Type I transmembrane protein family share structural characteristics including an extracellular domain consisting of multiple epidermal growth factor-like (EGF) repeats, and an intracellular domain consisting of multiple different domain types. Notch signaling is an evolutionarily conserved intercellular signaling pathway that regulates interactions between physically adjacent cells through binding of Notch family receptors to their cognate ligands. The encoded preproprotein is proteolytically processed in the trans-Golgi network to generate two polypeptide chains that heterodimerize to form the mature cell-surface receptor. This receptor plays a role in the development of numerous cell and tissue types. Mutations in this gene are associated with aortic valve disease, Adams-Oliver syndrome, T-cell acute lymphoblastic leukemia, chronic lymphocytic leukemia, and head and neck squamous cell carcinoma. |
| *NPM1* | Nucleophosmin 1 | Nucleophosmin; Nucleophosmin (Nucleolar Phosphoprotein B23, Numatrin); Nucleophosmin/Nucleoplasmin Family, Member 1; Nucleolar Phosphoprotein B23; Nucleolar Protein NO38; Numatrin; NPM; Testicular Tissue Protein Li 128; B23 | The protein encoded by this gene is involved in several cellular processes, including centrosome duplication, protein chaperoning, and cell proliferation. The encoded phosphoprotein shuttles between the nucleolus, nucleus, and cytoplasm, chaperoning ribosomal proteins and core histones from the nucleus to the cytoplasm. This protein is also known to sequester the tumor suppressor ARF in the nucleolus, protecting it from degradation until it is needed. Mutations in this gene are associated with acute myeloid leukemia. Dozens of pseudogenes of this gene have been identified. |
| *NRAS* | NRAS Proto-Oncogene, GTPase | Neuroblastoma RAS Viral (V-Ras) Oncogene Homolog; Neuroblastoma RAS Viral Oncogene Homolog; Transforming Protein N-Ras; GTPase NRas; V-Ras Neuroblastoma RAS Viral Oncogene Homolog; N-Ras Protein Part 4; ALPS4; N-Ras; NRAS1; HRAS1; CMNS; NCMS; NS6 | This is an N-*ras* oncogene encoding a membrane protein that shuttles between the Golgi apparatus and the plasma membrane. This shuttling is regulated through palmitoylation and depalmitoylation by the ZDHHC9-GOLGA7 complex. The encoded protein, which has intrinsic GTPase activity, is activated by a guanine nucleotide-exchange factor and inactivated by a GTPase activating protein. Mutations in this gene have been associated with somatic rectal cancer, follicular thyroid cancer, autoimmune lymphoproliferative syndrome, Noonan syndrome, and juvenile myelomonocytic leukemia. |
| *PBRM1* | Polybromo 1 | BRG1-Associated Factor 180; Protein Polybromo-1; Polybromo-1D; BAF180; PB1; HPB1 | This locus encodes a subunit of ATP-dependent chromatin-remodeling complexes. The encoded protein has been identified as in integral component of complexes necessary for ligand-dependent transcriptional activation by nuclear hormone receptors. Mutations at this locus have been associated with primary clear cell renal cell carcinoma. |
| *PRDM1* | PR/SET Domain 1 | PR Domain Containing 1, With ZNF Domain; PR Domain Zinc Finger Protein 1; PR Domain 1; PRDI-BF1; BLIMP-1; BLIMP1; Beta-Interferon Gene Positive-Regulatory Domain I Binding Factor; Beta-Interferon Gene Positive Regulatory Domain I-Binding Factor; Positive Regulatory Domain I-Binding Factor 1; B-Lymphocyte-Induced Maturation Protein 1; PR Domain-Containing Protein 1; PRDI-Binding Factor-1; PRDI-Binding Factor 1; EC 2.1.1.- | This gene encodes a protein that acts as a repressor of beta-interferon gene expression. The protein binds specifically to the PRDI (positive regulatory domain I element) of the beta-IFN gene promoter. Transcription of this gene increases upon virus induction. Two alternatively spliced transcript variants that encode different isoforms have been reported. |
| *PIM1* | Pim-1 Proto-Oncogene, Serine/Threonine Kinase | Serine/Threonine-Protein Kinase Pim-1; EC 2.7.11.1; Proto-Oncogene Serine/Threonine-Protein Kinase Pim-1; Pim-1 Oncogene (Proviral Integration Site 1); Pim-1 Kinase 44 KDa Isoform; Pim-1 Oncogene; Oncogene PIM1; PIM | The protein encoded by this gene belongs to the Ser/Thr protein kinase family, and PIM subfamily. This gene is expressed primarily in B-lymphoid and myeloid cell lines and is overexpressed in hematopoietic malignancies and in prostate cancer. It plays a role in signal transduction in blood cells, contributing to both cell proliferation and survival, and thus provides a selective advantage in tumorigenesis. Both the human and orthologous mouse genes have been reported to encode two isoforms (with preferential cellular localization) resulting from the use of alternative in-frame translation initiation codons, the upstream non-AUG (CUG) and downstream AUG codons |
| *PTCH1* | Patched 1 | Protein Patched Homolog 1; PTCH; PTC1; PTC; Patched Homolog 1 (Drosophila); Patched (Drosophila) Homolog; Patched Homolog (Drosophila); NBCCS; BCNS | This gene encodes a member of the patched family of proteins and a component of the hedgehog signaling pathway. Hedgehog signaling is important in embryonic development and tumorigenesis. The encoded protein is the receptor for the secreted hedgehog ligands, which include sonic hedgehog, Indian hedgehog and desert hedgehog. Following binding by one of the hedgehog ligands, the encoded protein is trafficked away from the primary cilium, relieving inhibition of the G-protein-coupled receptor smoothened, which results in activation of downstream signaling. Mutations of this gene have been associated with basal cell nevus syndrome and holoprosencephaly. |
| *PIK3CA* | Phosphatidylinositol-4,5-Bisphosphate 3-Kinase Catalytic Subunit Alpha | Phosphatidylinositol 4,5-Bisphosphate 3-Kinase Catalytic Subunit Alpha Isoform; Phosphoinositide-3-Kinase, Catalytic, Alpha Polypeptide; Serine/Threonine Protein Kinase PIK3CA; PtdIns-3-Kinase Subunit P110-Alpha; PI3K-Alpha; Phosphatidylinositol-4,5-Bisphosphate 3-Kinase Catalytic Subunit, Alpha Isoform; Phosphatidylinositol-4,5-Bisphosphate 3-Kinase 110 KDa Catalytic Subunit Alpha; Phosphatidylinositol 4,5-Bisphosphate 3-Kinase 110 KDa Catalytic Subunit Alpha; Phosphatidylinositol-4,5-Bisphosphate 3-Kinase, Catalytic Subunit Alpha; Phosphatidylinositol 3-Kinase, Catalytic, Alpha Polypeptide; Phosphatidylinositol 3-Kinase, Catalytic, 110-KD, Alpha; Phosphoinositide-3-Kinase Catalytic Alpha Polypeptide; PI3-Kinase P110 Subunit Alpha; PtdIns-3-Kinase Subunit Alpha; PI3-Kinase Subunit Alpha; EC 2.7.1.153; EC 2.7.11.1; P110-Alpha; PI3Kalpha; P110alpha; EC 2.7.1; CLAPO; CLOVE; MCMTC; CWS5; MCAP; PI3K; MCM | Phosphatidylinositol 3-kinase is composed of an 85 kDa regulatory subunit and a 110 kDa catalytic subunit. The protein encoded by this gene represents the catalytic subunit, which uses ATP to phosphorylate PtdIns, PtdIns4P and PtdIns(4,5)P2. This gene has been found to be oncogenic and has been implicated in cervical cancers. A pseudogene of this gene has been defined on chromosome 22. |
| *PREX2* | Phosphatidylinositol-3,4,5-Trisphosphate Dependent Rac Exchange Factor 2 | Phosphatidylinositol 3,4,5-Trisphosphate-Dependent Rac Exchanger 2 Protein; Protein Phosphatase 1, Regulatory Subunit 129; PtdIns(3,4,5)-Dependent Rac Exchanger 2; DEP Domain-Containing Protein 2; DEPDC2; Phosphatidylinositol-3,4,5-Trisphosphate-Dependent Rac Exchange Factor 2; DEP Domain Containing 2; PPP1R129; P-REX2; P-Rex2; DEP.2 | The protein encoded by this gene belongs to the phosphatidylinositol 3,4,5-trisphosphate (PIP3)-dependent Rac exchanger (PREX) family, which are Dbl-type guanine-nucleotide exchange factors for Rac family small G proteins. Structural domains of this protein include the catalytic diffuse B-cell lymphoma homology and pleckstrin homology (DHPH) domain, two disheveled, EGL-10, and pleckstrin homology (DEP) domains, two PDZ domains, and a C-terminal inositol polyphosphate-4 phosphatase (IP4P) domain that is found in one of the isoforms. This protein facilitates the exchange of GDP for GTP on Rac1, allowing the GTP-bound Rac1 to activate downstream effectors. Studies also show that the pleckstrin homology domain of this protein interacts with the phosphatase and tensin homolog (*PTEN*) gene product to inhibit PTEN phosphatase activity, thus activating the phosphoinositide-3 kinase (PI3K) signaling pathway. Conversely, the *PTEN* gene product has also been shown to inhibit the GEF activity of this protein. This gene plays a role in insulin-signaling pathways, and either mutations or overexpression of this gene have been observed in some cancers. |
| *PTEN* | Phosphatase and Tensin Homolog | Mutated In Multiple Advanced Cancers 1; Phosphatidylinositol 3,4,5-Trisphosphate 3-Phosphatase And Dual-Specificity Protein Phosphatase PTEN; MMAC1; TEP1; Phosphatidylinositol-3,4,5-Trisphosphate 3-Phosphatase And Dual-Specificity Protein Phosphatase PTEN; MMAC1 Phosphatase And Tensin Homolog Deleted On Chromosome 10; Mitochondrial Phosphatase And Tensin Protein Alpha; Phosphatase And Tensin-Like Protein; Protein Tyrosine Phosphatase; Mitochondrial PTENalpha; EC 3.1.3.16; EC 3.1.3.48; EC 3.1.3.67; 10q23del; PTENbeta; PTEN1; CWS1; GLM2; MHAM; DEC; BZS | This gene was identified as a tumor suppressor that is mutated in several cancers at high frequency. The protein encoded by this gene is a phosphatidylinositol-3,4,5-trisphosphate 3-phosphatase. It contains a tensin like domain as well as a catalytic domain similar to that of the dual specificity protein tyrosine phosphatases. Unlike most of the protein tyrosine phosphatases, this protein preferentially dephosphorylates phosphoinositide substrates. It negatively regulates intracellular levels of phosphatidylinositol-3,4,5-trisphosphate in cells and functions as a tumor suppressor by negatively regulating AKT/PKB signaling pathway. The use of a non-canonical (CUG) upstream initiation site produces a longer isoform that initiates translation with a leucine and is thought to be preferentially associated with the mitochondrial inner membrane. This longer isoform may help regulate energy metabolism in the mitochondria. A pseudogene of this gene is found on chromosome 9. Alternative splicing and the use of multiple translation start codons results in multiple transcript variants encoding different isoforms. |
| *PTPN11* | Protein Tyrosine Phosphatase, Non-Receptor Type 11 | Tyrosine-Protein Phosphatase Non-Receptor Type 11; Protein-Tyrosine Phosphatase 1D; Protein-Tyrosine Phosphatase 2C; EC 3.1.3.48; SH-PTP2; SH-PTP3; PTP-1D; PTP-2C; PTP2C; Noonan Syndrome 1; METCDS; SHPTP2; BPTP3; SHP-2; JMML; SHP2; Shp2; CFC; NS1 | The protein encoded by this gene is a member of the protein tyrosine phosphatase (PTP) family. PTPs are known to be signaling molecules that regulate a variety of cellular processes including cell growth, differentiation, mitotic cycle, and oncogenic transformation. This PTP contains two tandem Src homology-2 domains, which function as phospho-tyrosine binding domains and mediate the interaction of this PTP with its substrates. This PTP is widely expressed in most tissues and plays a regulatory role in various cell signaling events that are important for a diversity of cell functions, such as mitogenic activation, metabolic control, transcription regulation, and cell migration. Mutations in this gene are a cause of Noonan syndrome as well as acute myeloid leukemia. |
| *PTPRB* | Protein Tyrosine Phosphatase, Receptor Type B | Vascular Endothelial Protein Tyrosine Phosphatase; Receptor-Type Tyrosine-Protein Phosphatase Beta; EC 3.1.3.48; VE-PTP; PTPB; Protein Tyrosine Phosphatase, Receptor Type, Beta Polypeptide; Protein-Tyrosine Phosphatase Beta; R-PTP-BETA; R-PTP-Beta; HPTP-BETA; HPTPB; VEPTP | The protein encoded by this gene is a member of the protein tyrosine phosphatase (PTP) family. PTPs are known to be signaling molecules that regulate a variety of cellular processes including cell growth, differentiation, mitotic cycle, and oncogenic transformation. This PTP contains an extracellular domain, a single transmembrane segment and one intracytoplasmic catalytic domain, thus belongs to receptor type PTP. The extracellular region of this PTP is composed of multiple fibronectin type_III repeats, which was shown to interact with neuronal receptor and cell adhesion molecules, such as contactin and tenascin C. This protein was also found to interact with sodium channels, and thus may regulate sodium channels by altering tyrosine phosphorylation status. The functions of the interaction partners of this protein implicate the roles of this PTP in cell adhesion, neurite growth, and neuronal differentiation. Alternate transcript variants encoding different isoforms have been found for this gene. |
| *PTPRT* | Protein Tyrosine Phosphatase, Receptor Type T | Receptor-Type Tyrosine-Protein Phosphatase Rho; Receptor-Type Tyrosine-Protein Phosphatase T; EC 3.1.3.48; RPTP-Rho; R-PTP-T; Receptor Protein Tyrosine Phosphatase; KIAA0283; EC 3.1.3; RPTPrho | The protein encoded by this gene is a member of the protein tyrosine phosphatase (PTP) family. PTPs are known to be signaling molecules that regulate a variety of cellular processes including cell growth, differentiation, mitotic cycle, and oncogenic transformation. This PTP possesses an extracellular region, a single transmembrane region, and two tandem intracellular catalytic domains, and thus represents a receptor-type PTP. The extracellular region contains a meprin-A5 antigen-PTP (MAM) domain, Ig-like and fibronectin type III-like repeats. The protein domain structure and the expression pattern of the mouse counterpart of this PTP suggest its roles in both signal transduction and cellular adhesion in the central nervous system. Two alternatively spliced transcript variants of this gene, which encode distinct proteins, have been reported. |
| *RB1* | RB Transcriptional Corepressor 1 | Protein Phosphatase 1, Regulatory Subunit 130; Prepro-Retinoblastoma-Associated Protein; Retinoblastoma-Associated Protein; Retinoblastoma 1; P105-Rb; Pp110; PRb; Exon 17 Tumor GOS561 Substitution Mutation Causes Premature Stop; GOS563 Exon 17 Substitution Mutation Causes Premature Stop; Retinoblastoma Susceptibility Protein; Osteosarcoma; PPP1R130; OSRC; RB; Rb | The protein encoded by this gene is a negative regulator of the cell cycle and was the first tumor suppressor gene found. The encoded protein also stabilizes constitutive heterochromatin to maintain the overall chromatin structure. The active, hypophosphorylated form of the protein binds transcription factor E2F1. Defects in this gene are a cause of childhood cancer retinoblastoma (RB), bladder cancer, and osteogenic sarcoma. |
| *RBM10* | RNA Binding Motif Protein 10 | G Patch Domain-Containing Protein 9; RNA-Binding Protein S1-1; RNA-Binding Protein 10; DXS8237E; GPATCH9; GPATC9; S1-1; Epididymis Secretory Sperm Binding Protein; RNA-Binding Motif Protein 10; KIAA0122; ZRANB5; TARPS | This gene encodes a nuclear protein that belongs to a family of proteins that contain an RNA-binding motif. The encoded protein associates with hnRNP proteins and may be involved in regulating alternative splicing. Defects in this gene are the cause of the X-linked recessive disorder, TARP syndrome. Alternate splicing results in multiple transcript variants. |
| *RET* | Ret Proto-Oncogene | Proto-Oncogene Tyrosine-Protein Kinase Receptor Ret; Cadherin-Related Family Member 16; Rearranged During Transfection; RET Receptor Tyrosine Kinase; Cadherin Family Member 12; Proto-Oncogene C-Ret; EC 2.7.10.1; CDHF12; CDHR16; PTC; Ret Proto-Oncogene (Multiple Endocrine Neoplasia And Medullary Thyroid Carcinoma 1, Hirschsprung Disease); Multiple Endocrine Neoplasia And Medullary Thyroid Carcinoma 1; Hirschsprung Disease 1; EC 2.7.10; RET-ELE1; HSCR1; MEN2A; MEN2B; RET51; MTC1 | This gene encodes a transmembrane receptor and member of the tyrosine protein kinase family of proteins. Binding of ligands such as GDNF (glial cell-line derived neurotrophic factor) and other related proteins to the encoded receptor stimulates receptor dimerization and activation of downstream signaling pathways that play a role in cell differentiation, growth, migration and survival. The encoded receptor is important in development of the nervous system, and the development of organs and tissues derived from the neural crest. This proto-oncogene can undergo oncogenic activation through both cytogenetic rearrangement and activating point mutations. Mutations in this gene are associated with Hirschsprung disease and central hypoventilation syndrome and have been identified in patients with renal agenesis. |
| *RHOA* | Ras Homolog Family Member A | Transforming Protein RhoA; ARH12; RHO12; ARHA; Epididymis Secretory Sperm Binding Protein; Ras Homolog Gene Family, Member A; Aplysia Ras-Related Homolog 12; Small GTP Binding Protein RhoA; Rho CDNA Clone 12; Oncogene RHO H12; RHOH12; H12 | This gene encodes a member of the Rho family of small GTPases, which cycle between inactive GDP-bound and active GTP-bound states and function as molecular switches in signal transduction cascades. Rho proteins promote reorganization of the actin cytoskeleton and regulate cell shape, attachment, and motility. Overexpression of this gene is associated with tumor cell proliferation and metastasis. Multiple alternatively spliced variants have been identified. |
| *RNF43* | Ring Finger Protein 43 | RING-Type E3 Ubiquitin Transferase RNF43; E3 Ubiquitin-Protein Ligase RNF43; RING Finger Protein 43; EC 2.3.2.27; RNF124; SSPCS; URCC | The protein encoded by this gene is a RING-type E3 ubiquitin ligase and is predicted to contain a transmembrane domain, a protease-associated domain, an ectodomain, and a cytoplasmic RING domain. This protein is thought to negatively regulate Wnt signaling, and expression of this gene results in an increase in ubiquitination of frizzled receptors, an alteration in their subcellular distribution, resulting in reduced surface levels of these receptors. Mutations in this gene have been reported in multiple tumor cells, including colorectal and endometrial cancers. Alternative splicing results in multiple transcript variants encoding different isoforms. |
| *RUNX1* | Runt Related Transcription Factor 1 | Runt-Related Transcription Factor 1; Polyomavirus Enhancer-Binding Protein 2 Alpha B Subunit; SL3/AKV Core-Binding Factor Alpha B Subunit; SL3-3 Enhancer Factor 1 Alpha B Subunit; Acute Myeloid Leukemia 1 Protein; Oncogene AML-1; PEBP2-Alpha B; PEA2-Alpha B; CBFA2; AML1; Core-Binding Factor, Runt Domain, Alpha Subunit 2; Core-Binding Factor Subunit Alpha-2; AML1-EVI-1 Fusion Protein; Acute Myeloid Leukemia 1; Aml1 Oncogene; CBF-Alpha-2; AML1-EVI-1; PEBP2alpha; CBF2alpha; PEBP2aB; AMLCR1; EVI-1 | Core binding factor (CBF) is a heterodimeric transcription factor that binds to the core element of many enhancers and promoters. The protein encoded by this gene represents the alpha subunit of CBF and is thought to be involved in the development of normal hematopoiesis. Chromosomal translocations involving this gene are well-documented and have been associated with several types of leukemia. Three transcript variants encoding different isoforms have been found for this gene. |
| *SMAD4* | SMAD Family Member 4 | Deletion Target In Pancreatic Carcinoma 4; Mothers Against Decapentaplegic Homolog 4; MAD Homolog 4; MADH4; DPC4; MAD, Mothers Against Decapentaplegic Homolog 4 (Drosophila); Mothers Against Decapentaplegic, Drosophila, Homolog Of, 4; SMAD, Mothers Against DPP Homolog 4 (Drosophila); Deleted In Pancreatic Carcinoma Locus 4; SMAD, Mothers Against DPP Homolog 4; Mothers Against DPP Homolog 4; SMAD 4; HSMAD4; MYHRS; Smad4; JIP | This gene encodes a member of the Smad family of signal transduction proteins. Smad proteins are phosphorylated and activated by transmembrane serine-threonine receptor kinases in response to transforming growth factor (TGF)-beta signaling. The product of this gene forms homomeric complexes and heteromeric complexes with other activated Smad proteins, which then accumulate in the nucleus and regulate the transcription of target genes. This protein binds to DNA and recognizes an 8-bp palindromic sequence (GTCTAGAC) called the Smad-binding element (SBE). The protein acts as a tumor suppressor and inhibits epithelial cell proliferation. It may also have an inhibitory effect on tumors by reducing angiogenesis and increasing blood vessel hyperpermeability. The encoded protein is a crucial component of the bone morphogenetic protein signaling pathway. The Smad proteins are subject to complex regulation by post-translational modifications. Mutations or deletions in this gene have been shown to result in pancreatic cancer, juvenile polyposis syndrome, and hereditary hemorrhagic telangiectasia syndrome. |
| *SMARCA4* | SWI/SNF Related, Matrix Associated, Actin Dependent Regulator of Chromatin, Subfamily A, Member 4 | Mitotic Growth And Transcription Activator; ATP-Dependent Helicase SMARCA4; Global Transcription Activator Homologous Sequence; Transcription Activator BRG1; Sucrose Nonfermenting-Like 4; BRG1-Associated Factor 190A; Protein Brahma Homolog 1; BRM/SWI2-Related Gene 1; Homeotic Gene Regulator; Brahma Protein-Like 1; Nuclear Protein GRB1; Protein BRG-1; SNF2-Like 4; SNF2-Beta; BAF190A; SNF2L4; BRG1; SWI/SNF-Related Matrix-Associated Actin-Dependent Regulator Of Chromatin Subfamily A Member 4; EC 3.6.4.-; EC 3.6.1; BAF190; SNF2LB; HSNF2b; MRD16; RTPS2; SNF2B; CSS4; SNF2; SWI2 | The protein encoded by this gene is a member of the SWI/SNF family of proteins and is similar to the brahma protein of Drosophila. Members of this family have helicase and ATPase activities and are thought to regulate transcription of certain genes by altering the chromatin structure around those genes. The encoded protein is part of the large ATP-dependent chromatin remodeling complex SNF/SWI, which is required for transcriptional activation of genes normally repressed by chromatin. In addition, this protein can bind BRCA1, as well as regulate the expression of the tumorigenic protein CD44. Mutations in this gene cause rhabdoid tumor predisposition syndrome type 2. Multiple transcript variants encoding different isoforms have been found for this gene. |
| *SOCS1* | Suppressor of Cytokine Signaling 1 | STAT-Induced STAT Inhibitor 1; Tec-Interacting Protein 3; SOCS-1; SSI-1; TIP-3; SSI1; TIP3; JAB; Cytokine-Inducible SH2 Protein 1; STAT Induced SH3 Protein 1; JAK Binding Protein; JAK-Binding Protein; CISH1; CIS1 | This gene encodes a member of the STAT-induced STAT inhibitor (SSI), also known as suppressor of cytokine signaling (SOCS), family. SSI family members are cytokine-inducible negative regulators of cytokine signaling. The expression of this gene can be induced by a subset of cytokines, including IL2, IL3 erythropoietin (EPO), CSF2/GM-CSF, and interferon (IFN)-gamma. The protein encoded by this gene functions downstream of cytokine receptors and takes part in a negative feedback loop to attenuate cytokine signaling. Knockout studies in mice suggested the role of this gene as a modulator of IFN-gamma action, which is required for normal postnatal growth and survival. |
| *SPEN* | Spen Family Transcriptional Repressor | SMART/HDAC1-Associated Repressor Protein; Msx2-Interacting Protein; SHARP; MINT; SPEN Homolog, Transcriptional Regulator (Drosophila); Spen Homolog, Transcriptional Regulator (Drosophila); Msx2 Interacting Nuclear Target (MINT) Homolog; Nuclear Receptor Transcription Cofactor; Spen Homolog, Transcriptional Regulator; SPEN Homolog; HIAA0929; KIAA0929; RBM15C | This gene encodes a hormone inducible transcriptional repressor. Repression of transcription by this gene product can occur through interactions with other repressors, by the recruitment of proteins involved in histone deacetylation, or through sequestration of transcriptional activators. The product of this gene contains a carboxy-terminal domain that permits binding to other corepressor proteins. This domain also permits interaction with members of the NuRD complex, a nucleosome remodeling protein complex that contains deacetylase activity. In addition, this repressor contains several RNA recognition motifs that confer binding to a steroid receptor RNA coactivator; this binding can modulate the activity of both liganded and nonliganded steroid receptors. |
| *SPOP* | Speckle Type BTB/POZ Protein | Speckle-Type POZ Protein; Roadkill Homolog 1; HIB Homolog 1; BTBD32; TEF2 | This gene encodes a protein that may modulate the transcriptional repression activities of death-associated protein 6 (DAXX), which interacts with histone deacetylase, core histones, and other histone-associated proteins. In mouse, the encoded protein binds to the putative leucine zipper domain of macroH2A1.2, a variant H2A histone that is enriched on inactivated X chromosomes. The BTB/POZ domain of this protein has been shown in other proteins to mediate transcriptional repression and to interact with components of histone deacetylase co-repressor complexes. Alternative splicing of this gene results in multiple transcript variants encoding the same protein. |
| *SRSF2* | Serine And Arginine Rich Splicing Factor 2 | Splicing Factor, Arginine/Serine-Rich 2; Serine/Arginine-Rich Splicing Factor 2; Splicing Component, 35 KDa; Splicing Factor SC35; SR Splicing Factor 2; SFRS2; SC-35; Protein PR264; SFRS2A; SRp30b; PR264; SC35 | The protein encoded by this gene is a member of the serine/arginine (SR)-rich family of pre-mRNA splicing factors, which constitute part of the spliceosome. Each of these factors contains an RNA recognition motif (RRM) for binding RNA and an RS domain for binding other proteins. The RS domain is rich in serine and arginine residues and facilitates interaction between different SR splicing factors. In addition to being critical for mRNA splicing, the SR proteins have also been shown to be involved in mRNA export from the nucleus and in translation. Two transcript variants encoding the same protein and one non-coding transcript variant have been found for this gene. In addition, a pseudogene of this gene has been found on chromosome 11. |
| *STAT6* | Signal Transducer And Activator Of Transcription 6 | Signal Transducer and Activator Of Transcription 6, Interleukin-4 Induced; Transcription Factor IL-4 STAT; STAT, Interleukin4-Induced; EC 2.4.1.227; EC 2.7.7.6; IL-4-STAT; IL-4 Stat; D12S1644; STAT6B; STAT6C | The protein encoded by this gene is a member of the STAT family of transcription factors. In response to cytokines and growth factors, STAT family members are phosphorylated by the receptor associated kinases, and then form homo- or heterodimers that translocate to the cell nucleus where they act as transcription activators. This protein plays a central role in exerting IL4 mediated biological responses. It is found to induce the expression of BCL2L1/BCL-X(L), which is responsible for the anti-apoptotic activity of IL4. Knockout studies in mice suggested the roles of this gene in differentiation of T helper 2 (Th2) cells, expression of cell surface markers, and class switch of immunoglobulins. Alternative splicing results in multiple transcript variants. |
| *STK11* | Serine/Threonine Kinase 11 | Serine/Threonine-Protein Kinase STK11; Renal Carcinoma Antigen NY-REN-19; Polarization-Related Protein LKB1; Liver Kinase B1; EC 2.7.11.1; HLKB1; LKB1; PJS; Serine/Threonine Kinase 11 (Peutz-Jeghers Syndrome); Serine/Threonine-Protein Kinase LKB1; Serine/Threonine-Protein Kinase 11 | This gene, which encodes a member of the serine/threonine kinase family, regulates cell polarity and functions as a tumor suppressor. Mutations in this gene have been associated with Peutz-Jeghers syndrome, an autosomal dominant disorder characterized by the growth of polyps in the gastrointestinal tract, pigmented macules on the skin and mouth, and other neoplasms. Alternate transcriptional splice variants of this gene have been observed but have not been thoroughly characterized. |
| *TBX3* | T-Box 3 | T-Box Transcription Factor TBX3; T-Box Protein 3; Bladder Cancer Related Protein XHL; Ulnar Mammary Syndrome; TBX3-ISO; XHL; UMS | This gene is a member of a phylogenetically conserved family of genes that share a common DNA-binding domain, the T-box. T-box genes encode transcription factors involved in the regulation of developmental processes. This protein is a transcriptional repressor and is thought to play a role in the anterior/posterior axis of the tetrapod forelimb. Mutations in this gene cause ulnar-mammary syndrome, affecting limb, apocrine gland, tooth, hair, and genital development. Alternative splicing of this gene results in three transcript variants encoding different isoforms; however, the full-length nature of one variant has not been determined. |
| *TCF7L2* | Transcription Factor 7 Like 2 | Transcription Factor 7-Like 2 (T-Cell Specific, HMG-Box); T-Cell-Specific Transcription Factor 4; HMG Box Transcription Factor 4; Transcription Factor 7-Like 2; T-Cell Factor 4; HTCF-4; TCF-4; TCF4 | This gene encodes a high mobility group (HMG) box-containing transcription factor that plays a key role in the Wnt signaling pathway. The protein has been implicated in blood glucose homeostasis. Genetic variants of this gene are associated with increased risk of type 2 diabetes. Several transcript variants encoding multiple different isoforms have been found for this gene. |
| *TERT* | Telomerase Reverse Transcriptase | Telomerase-Associated Protein 2; Telomerase Catalytic Subunit; EC 2.7.7.49; HEST2; EST2; TCS1; TP2; TRT; EC 2.7.7; PFBMFT1; DKCA2; DKCB4; CMM9; HTRT | Telomerase is a ribonucleoprotein polymerase that maintains telomere ends by addition of the telomere repeat TTAGGG. The enzyme consists of a protein component with reverse transcriptase activity, encoded by this gene, and an RNA component which serves as a template for the telomere repeat. Telomerase expression plays a role in cellular senescence, as it is normally repressed in postnatal somatic cells resulting in progressive shortening of telomeres. Deregulation of telomerase expression in somatic cells may be involved in oncogenesis. Studies in mouse suggest that telomerase also participates in chromosomal repair, since de novo synthesis of telomere repeats may occur at double-stranded breaks. Alternatively spliced variants encoding different isoforms of telomerase reverse transcriptase have been identified; the full-length sequence of some variants has not been determined. Alternative splicing at this locus is thought to be one mechanism of regulation of telomerase activity. |
| *TET2* | Tet Methylcytosine Dioxygenase 2 | KIAA1546; Methylcytosine Dioxygenase TET2; Tet Oncogene Family Member 2; EC 1.14.11.n2; Probable Methylcytosine Dioxygenase TET2; MDS | The protein encoded by this gene is a methylcytosine dioxygenase that catalyzes the conversion of methylcytosine to 5-hydroxymethylcytosine. The encoded protein is involved in myelopoiesis, and defects in this gene have been associated with several myeloproliferative disorders. Two variants encoding different isoforms have been found for this gene. |
| *TGFBR2* | Transforming Growth Factor Beta Receptor 2 | Transforming Growth Factor, Beta Receptor II (70/80kDa); Transforming Growth Factor Beta Receptor II; TGF-Beta Type II Receptor; TGF-Beta Receptor Type-2; EC 2.7.11.30; TbetaR-II; TGFR-2; Transforming Growth Factor, Beta Receptor II Epsilon; Transforming Growth Factor, Beta Receptor II Alpha; Transforming Growth Factor, Beta Receptor II Delta; Transforming Growth Factor, Beta Receptor II Gamma; Transforming Growth Factor Beta Receptor Type IIC; Transforming Growth Factor, Beta Receptor II Beta; Transforming Growth Factor-Beta Receptor Type II; TGF-Beta Receptor Type IIB; TGF-Beta Receptor Type II; TGFbeta-RII; EC 2.7.11; TBR-Ii; LDS1B; LDS2B; TAAD2; TBRII; AAT3; FAA3; LDS2; RIIC; MFS2 | The protein encoded by this gene is a transmembrane protein that has a protein kinase domain, forms a heterodimeric complex with TGF-beta receptor type-1, and binds TGF-beta. This receptor/ligand complex phosphorylates proteins, which then enter the nucleus and regulate the transcription of genes related to cell proliferation, cell cycle arrest, wound healing, immunosuppression, and tumorigenesis. Mutations in this gene have been associated with Marfan Syndrome, Loeys-Deitz Aortic Aneurysm Syndrome, and the development of various types of tumors. Alternatively spliced transcript variants encoding different isoforms have been characterized. |
| *TNFAIP3* | TNF Alpha Induced Protein 3 | Tumor Necrosis Factor Alpha-Induced Protein 3; Putative DNA-Binding Protein A20; OTU Domain-Containing Protein 7C; Zinc Finger Protein A20; OTUD7C; Tumor Necrosis Factor, Alpha Induced Protein 3; Tumor Necrosis Factor, Alpha-Induced Protein 3; Tumor Necrosis Factor Inducible Protein A20; TNF Alpha-Induced Protein 3; EC 3.4.19.12; EC 2.3.2.-; TNFA1P2; AISBL; A20 | This gene was identified as a gene whose expression is rapidly induced by the tumor necrosis factor (TNF). The protein encoded by this gene is a zinc finger protein and ubiquitin-editing enzyme and has been shown to inhibit NF-kappa B activation as well as TNF-mediated apoptosis. The encoded protein, which has both ubiquitin ligase and deubiquitinase activities, is involved in the cytokine-mediated immune and inflammatory responses. Several transcript variants encoding the same protein have been found for this gene. |
| *TNFRSF14* | TNF Receptor Superfamily Member 14 | Tumor Necrosis Factor Receptor Superfamily, Member 14 (Herpesvirus Entry Mediator); Tumor Necrosis Factor Receptor Superfamily Member 14; Herpes Virus Entry Mediator A; HVEA; HVEM; TR2; Tumor Necrosis Factor Receptor Superfamily, Member 14; Tumor Necrosis Factor Receptor-Like Gene2; Tumor Necrosis Factor Receptor-Like 2; Herpesvirus Entry Mediator A; Herpesvirus Entry Mediator; CD40-Like Protein; CD270 Antigen; LIGHTR; CD270; ATAR; HveA | This gene encodes a member of the TNF (tumor necrosis factor) receptor superfamily. The encoded protein functions in signal transduction pathways that activate inflammatory and inhibitory T-cell immune response. It binds herpes simplex virus (HSV) viral envelope glycoprotein D (gD), mediating its entry into cells. Alternative splicing results in multiple transcript variants. |
| *TP53* | Tumor Protein P53 | Cellular Tumor Antigen P53; Phosphoprotein P53; Antigen NY-CO-13; P53; Transformation-Related Protein 53; Mutant Tumor Protein 53; P53 Tumor Suppressor; Tumor Suppressor P53; Li-Fraumeni Syndrome; Tumor Suppressor P53; Tumor Protein 53; TRP53; BCC7; LFS1 | This gene encodes a tumor suppressor protein containing transcriptional activation, DNA binding, and oligomerization domains. The encoded protein responds to diverse cellular stresses to regulate expression of target genes, thereby inducing cell cycle arrest, apoptosis, senescence, DNA repair, or changes in metabolism. Mutations in this gene are associated with a variety of human cancers, including hereditary cancers such as Li-Fraumeni syndrome. Alternative splicing of this gene and the use of alternate promoters result in multiple transcript variants and isoforms. Additional isoforms have also been shown to result from the use of alternate translation initiation codons from identical transcript variants. |
| *TRRAP* | Transformation/Transcription Domain Associated Protein | 350/400 KDa PCAF-Associated Factor; Tra1 Homolog; PAF350/400; PAF400; STAF40; TR-AP; Tra1 | This gene encodes a large multidomain protein of the phosphoinositide 3-kinase-related kinases (PIKK) family. The encoded protein is a common component of many histone acetyltransferase (HAT) complexes and plays a role in transcription and DNA repair by recruiting HAT complexes to chromatin. Deregulation of this gene may play a role in several types of cancer including glioblastoma multiforme. Alternatively spliced transcript variants encoding multiple isoforms have been observed for this gene. |
| *TSC2* | TSC Complex Subunit 2 | Protein Phosphatase 1, Regulatory Subunit 160; Tuberous Sclerosis 2 Protein; Tuberin; TSC4; Tuberous Sclerosis 2; PPP1R160; LAM | Mutations in this gene lead to tuberous sclerosis complex. Its gene product is believed to be a tumor suppressor that can stimulate specific GTPases. The protein associates with hamartin in a cytosolic complex, possibly acting as a chaperone for hamartin. Alternative splicing results in multiple transcript variants encoding different isoforms. |
| *WT1* | Wilms Tumor 1 | Wilms Tumor Protein; WT33; NPHS4; WIT-2; AWT1; WAGR; GUD | This gene encodes a transcription factor that contains four zinc-finger motifs at the C-terminus and a proline/glutamine-rich DNA-binding domain at the N-terminus. It has an essential role in the normal development of the urogenital system, and it is mutated in a small subset of patients with Wilms tumor. This gene exhibits complex tissue-specific and polymorphic imprinting pattern, with biallelic, and monoallelic expression from the maternal and paternal alleles in different tissues. Multiple transcript variants have been described. In several variants, there is evidence for the use of a non-AUG (CUG) translation initiation codon upstream of, and in-frame with the first AUG. Authors of PMID:7926762 also provide evidence that WT1 mRNA undergoes RNA editing in human and rat, and that this process is tissue-restricted and developmentally regulated. |
| *ZFHX3* | Zinc Finger Homeobox 3 | AT-Binding Transcription Factor 1; Zinc Finger Homeodomain Protein 3; Zinc Finger Homeobox Protein 3; AT Motif-Binding Factor 1; ATBF1; ZFH-3; Alpha-Fetoprotein Enhancer Binding Protein; Alpha-Fetoprotein Enhancer-Binding Protein; ZNF927; ATBT | This gene encodes a transcription factor with multiple homeodomains and zinc finger motifs and regulates myogenic and neuronal differentiation. The encoded protein suppresses expression of the alpha-fetoprotein gene by binding to an AT-rich enhancer motif. The protein has also been shown to negatively regulate c-Myb, and transactivate the cell cycle inhibitor cyclin-dependent kinase inhibitor 1A (also known as p21CIP1). This gene is reported to function as a tumor suppressor in several cancers, and sequence variants of this gene are also associated with atrial fibrillation. Multiple transcript variants expressed from alternate promoters and encoding different isoforms have been found for this gene. |
| *ZNF21* | Zinc Finger Protein 182 | Zinc Finger Protein 21 (KOX 14); Zinc Finger Protein KOX14; ZNF21; KOX14; Zinc Finger Protein 182 (HHZ150); Zinc Finger Protein 21; HHZ150; Zfp182 | Zinc-finger proteins bind nucleic acids and play important roles in various cellular functions, including cell proliferation, differentiation, and apoptosis. This gene encodes a zinc finger protein and belongs to the krueppel C2H2-type zinc-finger protein family. It may be involved in transcriptional regulation. Multiple alternatively spliced transcript variants encoding different isoforms have been identified. |
| *ZNF521*^2^ | Zinc Finger Protein 521 | Early Hematopoietic Zinc Finger Protein; LYST-Interacting Protein 3; EHZF; Early Hematopoietic Zinc Finger; Evi3; LIP3 | Northern blot analysis showed that expression of ZNF521 was high in CD34-positive hematopoietic progenitor cells, and its expression decreased following cytokine-induced differentiation. In response to BMP2 or BMP4, ZNF521 complexed SMAD1 and SMAD4 and bound to and enhanced transcriptional activity of a BMP-responsive element. ZNF521 inhibited transcriptional activity of early B-cell factor (EBF), a transcription actor essential for specification of B-cell lineage.  A zinc finger nuclear protein essential and sufficient for driving the intrinsic neural differentiation of mouse embryonic stem (ES) cells. In the absence of the neural differentiation inhibitor Bmp4, strong ZFP521 expression is intrinsically induced in differentiating ES cells. Forced expression of ZFP521 enables the neural conversion of ES cells even in the presence of BMP4. Conversely, in differentiation culture, ZFP521-depleted ES cells do not undergo neural conversion but tend to halt at the epiblast state. ZFP521 directly activates early neural genes by working with the coactivator p300. Thus, the transition of ES cell differentiation from the epiblast state into neuroectodermal progenitors specifically depends on the cell-intrinsic expression and activator function of ZFP521. |

^1^Data collected from: GeneCards, Human Gene Database, <https://www.genecards.org/>

^2^Data assembled from: OMIM, Online Mendelian Inheritance in Man, An Online Catalog of Human Genes and Genetic Disorders (March 29, 2019 versions), <https://www.omim.org/>

**Supporting Information Table 3. Cancer type distribution of the 94 most highly-mutated genes in the ten deadliest cancers.**

| **Gene** | **Trachae Bronchus Lung** | **Colon Rectum** | **Stomach** | **Liver** | **Breast** | **Pancreas** | **Esophagus** | **Prostate** | **Leukemia (AML)** | **Non-Hodgkin Lymphoma (DLBCL)** | **Total** |
| --- | --- | --- | --- | --- | --- | --- | --- | --- | --- | --- | --- |
| *ACVR2A* |  | X |  |  |  | X |  |  |  |  | 2 |
| *AKT1* |  |  |  |  | X |  |  |  |  |  | 1 |
| *APC* |  | X | X | X |  | X |  | X |  |  | 5 |
| *AR* |  |  |  |  |  |  |  | X |  |  | 1 |
| *ARID1A* | X | X | X | X | X | X | X |  |  |  | 7 |
| *ARID2* |  |  |  | X |  |  |  |  |  |  | 1 |
| *ASXL1* |  |  |  |  |  |  |  |  | X |  | 1 |
| *ATM* | X |  |  | X |  | X |  | X | X |  | 5 |
| *AXIN* |  |  |  | X |  |  |  |  |  |  | 1 |
| *B2M* |  |  |  |  |  |  |  |  |  | X | 1 |
| *BCL2* |  |  |  |  |  |  |  |  |  | X | 1 |
| *BRAF* |  | X |  |  |  |  |  |  |  |  | 1 |
| *BRCA2* |  |  |  |  |  | X | X | X |  |  | 3 |
| *BTG1* |  |  |  |  |  |  |  |  |  | X | 1 |
| *CARD11* |  |  |  |  |  |  |  |  |  | X | 1 |
| *CD79B* |  |  |  |  |  |  |  |  |  | X | 1 |
| *CDH1* |  |  | X |  | X |  |  |  |  |  | 2 |
| *CDK12* |  |  |  |  |  |  |  | X |  |  | 1 |
| *CDKN2A* | X |  |  | X |  | X | X |  |  | X | 5 |
| *CEBPA* |  |  |  |  |  |  |  |  | X |  | 1 |
| *CREBBP* |  |  |  |  |  |  | X |  |  | X | 2 |
| *CTNNB1* |  |  |  | X |  | X |  | X |  |  | 3 |
| *DNM2* |  |  |  |  |  |  |  |  | X |  | 1 |
| *DNMT3A* |  |  |  |  |  |  |  |  | X |  | 1 |
| *EGFR* | X |  |  |  |  |  |  |  |  |  | 1 |
| *EP300* |  |  |  |  |  |  | X |  |  |  | 1 |
| *ERBB2* |  |  |  |  | X |  |  |  |  |  | 1 |
| *ERBB3* |  |  | X |  |  |  |  |  |  |  | 1 |
| *ERBB4* |  |  |  |  |  |  | X |  |  |  | 1 |
| *ESR1* |  |  |  |  | X |  |  |  |  |  | 1 |
| *EZH2* |  |  |  |  |  |  |  |  |  | X | 1 |
| *FAT1* | X | X | X |  |  |  | X |  |  |  | 4 |
| *FAT4* | X | X | X | X |  | X | X | X |  |  | 7 |
| *FBXW7* |  | X |  |  |  |  | X |  |  |  | 2 |
| *FLT3* |  |  |  |  |  |  |  |  | X |  | 1 |
| *FOXA1* |  |  |  |  | X |  |  | X |  |  | 2 |
| *GATA3* |  |  |  |  | X |  |  |  |  |  | 1 |
| *GNAS* |  |  |  |  |  | X |  |  |  |  | 1 |
| *GRIN2A* | X |  |  |  |  |  |  | X |  |  | 2 |
| *IDH1* |  |  |  |  |  |  |  |  | X |  | 1 |
| *IDH2* |  |  |  |  |  |  |  |  | X |  | 1 |
| *KEAP1* | X |  |  |  |  |  |  |  |  |  | 1 |
| *KIT* |  |  |  |  |  |  |  |  | X |  | 1 |
| *KMT2A* |  |  |  | X |  |  |  |  |  |  | 1 |
| *KMT2C* | X | X | X | X | X | X | X | X |  | X | 9 |
| *KMT2D* | X | X | X | X | X | X | X | X |  | x | 9 |
| *KRAS* | X | X | X |  |  | X |  | X |  |  | 5 |
| *LRP1B* | X | X | X | X | X | X | X | X |  | X | 9 |
| *MAP2K2* |  |  |  |  |  |  |  |  | X |  | 1 |
| *MAP2K4* |  |  |  |  | X |  |  |  |  |  | 1 |
| *MYD88* |  |  |  |  |  |  |  |  |  | X | 1 |
| *NCOR1* |  |  |  |  | X |  |  |  |  |  | 1 |
| *NF1* | X |  |  |  | X |  |  |  |  |  | 2 |
| *NFE2L2* |  |  |  | X |  |  | X |  |  |  | 2 |
| *NOTCH1* |  |  |  |  |  |  | X |  |  |  | 1 |
| *NPM1* |  |  |  |  |  |  |  |  | X |  | 1 |
| *NRAS* |  |  |  |  |  |  |  |  | X |  | 1 |
| *PBRM1* |  |  |  |  |  |  |  |  | X |  | 1 |
| *PRDM1* |  |  |  |  |  |  |  |  |  | X | 1 |
| *PIM1* |  |  |  |  |  |  |  |  |  | X | 1 |
| *PTCH1* |  |  |  |  |  |  | X |  |  |  | 1 |
| *PIK3CA* |  | X | X | X | X | X | X | X |  |  | 7 |
| *PREX2* | X | X | X | X |  |  | X |  |  |  | 5 |
| *PTEN* |  |  |  |  | X |  |  | X |  |  | 2 |
| *PTPN11* |  |  |  |  |  |  |  |  | X |  | 1 |
| *PTPRB* |  |  |  | X |  |  |  |  |  |  | 1 |
| *PTPRT* | X |  | X |  |  |  | X |  |  |  | 3 |
| *RB1* | X |  |  |  | X |  |  | X |  |  | 3 |
| *RBM10* |  |  |  |  |  | X |  |  |  |  | 1 |
| *RET* |  |  |  |  |  |  |  |  | X |  | 1 |
| *RHOA* |  |  | X |  |  |  |  |  |  |  | 1 |
| *RNF43* |  | X | X |  |  | X |  |  |  |  | 3 |
| *RUNX1* |  |  |  |  | X |  |  |  | X |  | 2 |
| *SMAD4* |  | X |  |  |  | X |  |  |  |  | 2 |
| *SMARCA4* | X |  |  |  |  |  |  |  |  |  | 1 |
| *SOCS1* |  |  |  |  |  |  |  |  |  | X | 1 |
| *SPEN* |  |  | X |  | X |  |  | X |  |  | 3 |
| *SPOP* |  |  |  |  |  |  |  | X |  |  | 1 |
| *SRSF2* |  |  |  |  |  |  |  |  | X |  | 1 |
| *STAT6* |  |  |  |  |  |  |  |  |  | X | 1 |
| *STK11* | X |  |  |  |  |  |  |  |  |  | 1 |
| *TBX3* |  |  |  |  | X |  |  |  |  |  | 1 |
| *TCF7L2* |  | X |  |  |  |  |  |  |  |  | 1 |
| *TERT* |  |  |  | X |  |  |  |  |  |  | 1 |
| *TET2* |  |  |  |  |  |  |  |  | X | X | 2 |
| *TGFBR2* |  | X | X |  |  | X |  |  |  |  | 3 |
| *TNFAIP3* |  |  |  |  |  |  |  |  |  | X | 1 |
| *TNFRSF14* |  |  |  |  |  |  |  |  |  | X | 1 |
| *TP53* | X | X | X | X | X | X | X | X | X | X | 10 |
| *TRRAP* |  | X | X | X |  |  |  |  |  |  | 3 |
| *TSC2* |  |  |  | X |  |  |  |  |  |  | 1 |
| *WT1* |  |  |  |  |  |  |  |  | X |  | 1 |
| *ZFHX3* |  | X | X |  |  |  | X | X |  |  | 4 |
| *ZNF21* | X |  |  |  |  |  |  |  |  |  | 1 |
| *ZNF521* |  |  |  |  |  | X |  |  |  |  | 1 |

**Supporting Information Table 4. Hotspot CDMs within the 94 most highly-mutated genes in the ten deadliest cancers (CDMs representing >1% of a cancer type).**

| **Gene ID** | **Hotspot Codon** | **Cancer Type^1^** | **Percent Gene-specific Mutant Cancers each Codon Represents** | **Percent Total Cancers the Codon Represents** | **Percent Mutated Gene Incidence across 10 Deadliest Cancer Types^2^** |
| --- | --- | --- | --- | --- | --- |
| *AKT1* | E17 | Breast | 86.14 | 2.44 | 0.24 |
| *APC* | R213 | Large Intestine | 3.22 | 1.40 | 1.42 |
|  | R216 | Large Intestine | 5.75 | 2.51 |  |
|  | R876 | Large Intestine | 4.66 | 2.03 |  |
|  | R1114 | Large Intestine | 7.19 | 3.14 |  |
|  | R1450 | Large Intestine | 6.04 | 2.64 |  |
| *AR* | L702 | Prostate | 17.91 | 1.32 | 0.48 |
|  | H875 | Prostate | 23.99 | 1.76 |  |
|  | T878 | Prostate | 23.31 | 1.71 |  |
| *ATM* | D1853 | Acute Myeloid Leukemia | 25.74 | 2.09 | 0.90 |
|  | N1983 | Acute Myeloid Leukemia | 96.04 | 7.80 |  |
| *B2M* | M1 | Diffuse Large B Cell Lymphoma | 26.09 | 2.88 | 0.29 |
| *BCL2* | A2 | Diffuse Large B Cell Lymphoma | 6.17 | 1.62 | 2.47 |
|  | R6 | Diffuse Large B Cell Lymphoma | 7.41 | 1.95 |  |
|  | T7 | Diffuse Large B Cell Lymphoma | 13.58 | 3.57 |  |
|  | K17 | Diffuse Large B Cell Lymphoma | 4.94 | 1.30 |  |
|  | K22 | Diffuse Large B Cell Lymphoma | 4.94 | 1.30 |  |
|  | G47 | Diffuse Large B Cell Lymphoma | 6.17 | 1.62 |  |
|  | P59 | Diffuse Large B Cell Lymphoma | 6.17 | 1.62 |  |
|  | A60 | Diffuse Large B Cell Lymphoma | 7.41 | 1.95 |  |
|  | T74 | Diffuse Large B Cell Lymphoma | 6.17 | 1.62 |  |
|  | L86 | Diffuse Large B Cell Lymphoma | 4.94 | 1.30 |  |
|  | S87 | Diffuse Large B Cell Lymphoma | 7.41 | 1.95 |  |
|  | R129 | Diffuse Large B Cell Lymphoma | 4.94 | 1.30 |  |
|  | A131 | Diffuse Large B Cell Lymphoma | 13.58 | 3.57 |  |
| *BRAF* | V600 | Large Intestine | 97.43 | 9.68 | 0.97 |
| *BTG1* | Q36 | Diffuse Large B Cell Lymphoma | 22.73 | 2.14 | 0.21 |
| *CD79B* | Y196 | Diffuse Large B Cell Lymphoma | 78.00 | 7.96 | 0.80 |
| *CREBBP* | R1446 | Diffuse Large B Cell Lymphoma | 10.78 | 1.85 | 0.40 |
|  | Y1503 | Diffuse Large B Cell Lymphoma | 12.75 | 2.18 |  |
| *CTNNB1* | D32 | Liver | 14.06 | 2.61 | 2.14 |
|  | D32 | Pancreas | 33.77 | 2.73 |  |
|  | S33 | Liver | 12.03 | 2.24 |  |
|  | S33 | Pancreas | 15.58 | 1.26 |  |
|  | G34 | Liver | 8.54 | 1.59 |  |
|  | G34 | Pancreas | 15.58 | 1.26 |  |
|  | S37 | Liver | 9.02 | 1.68 |  |
|  | S37 | Pancreas | 25.97 | 2.10 |  |
|  | T41 | Liver | 14.06 | 2.61 |  |
|  | S45 | Liver | 17.65 | 3.28 |  |
| *DNM2* | A713 | Acute Myeloid Leukemia | 84.38 | 5.71 | 0.57 |
| *DNMT3A* | R882 | Acute Myeloid Leukemia | 61.09 | 10.92 | 1.09 |
| *EGFR* | T790 | Lung | 5.74 | 1.48 | 0.43 |
|  | L858 | Lung | 10.96 | 2.83 |  |
| *ERBB2* | L755 | Breast | 27.65 | 1.38 | 0.14 |
| *ESR1* | K303 | Breast | 34.70 | 2.34 | 0.57 |
|  | Y537 | Breast | 24.25 | 1.64 |  |
|  | D538 | Breast | 25.37 | 1.71 |  |
| *EZH2* | Y646 | Diffuse Large B Cell Lymphoma | 75.85 | 5.67 | 0.57 |
| *FBXW7* | R465 | Large Intestine | 25.22 | 2.31 | 0.33 |
|  | R505 | Large Intestine | 11.21 | 1.03 |  |
| *FLT3* | D835 | Acute Myeloid Leukemia | 8.01 | 1.91 | 0.19 |
| *GNAS* | R201 | Pancreas | 99.12 | 6.20 | 0.62 |
| *IDH1* | R132 | Acute Myeloid Leukemia | 25.94 | 1.64 | 0.16 |
| *IDH2* | R140 | Acute Myeloid Leukemia | 72.50 | 6.80 | 0.87 |
|  | R172 | Acute Myeloid Leukemia | 19.88 | 1.86 |  |
| *KIT* | D816 | Acute Myeloid Leukemia | 51.85 | 5.67 | 0.70 |
|  | N822 | Acute Myeloid Leukemia | 12.25 | 1.34 |  |
| *KRAS* | G12 | Large Intestine | 77.28 | 26.36 | 11.77 |
|  | G12 | Lung | 92.74 | 14.92 |  |
|  | G12 | Pancreas | 97.65 | 60.79 |  |
|  | G12 | Prostate | 75.25 | 1.96 |  |
|  | G12 | Stomach | 62.11 | 3.10 |  |
|  | G13 | Large Intestine | 20.02 | 6.83 |  |
|  | G13 | Lung | 6.77 | 1.09 |  |
|  | G13 | Pancreas | 2.03 | 1.26 |  |
|  | G13 | Stomach | 27.75 | 1.39 |  |
| *MAP2K2* | V64 | Acute Myeloid Leukemia | 18.18 | 1.69 | 1.19 |
|  | D151 | Acute Myeloid Leukemia | 29.55 | 2.75 |  |
|  | I220 | Acute Myeloid Leukemia | 80.68 | 7.50 |  |
| *MYD88* | L265 | Diffuse Large B Cell Lymphoma | 83.39 | 13.21 | 1.32 |
| *NRAS* | G12 | Acute Myeloid Leukemia | 48.22 | 5.80 | 1.24 |
|  | G13 | Acute Myeloid Leukemia | 29.51 | 3.55 |  |
|  | Q61 | Acute Myeloid Leukemia | 25.50 | 3.07 |  |
| *PBRM1* | T737 | Acute Myeloid Leukemia | 77.22 | 6.44 | 1.95 |
|  | P1199 | Acute Myeloid Leukemia | 78.48 | 6.55 |  |
|  | P1445 | Acute Myeloid Leukemia | 78.48 | 6.55 |  |
| *PIK3CA* | E542 | Breast | 12.58 | 3.37 | 4.56 |
|  | E542 | Large Intestine | 18.34 | 2.48 |  |
|  | E542 | Stomach | 13.33 | 1.07 |  |
|  | E545 | Breast | 23.63 | 6.34 |  |
|  | E545 | Esophagus | 61.43 | 4.36 |  |
|  | E545 | Large Intestine | 35.11 | 4.74 |  |
|  | E545 | Stomach | 32.96 | 2.65 |  |
|  | H1047 | Breast | 55.76 | 14.95 |  |
|  | H1047 | Large Intestine | 24.31 | 3.28 |  |
|  | H1047 | Stomach | 28.89 | 2.32 |  |
| *RBM10* | E698 | Pancreas | 62.22 | 2.11 | 0.21 |
| *RET* | A45 | Acute Myeloid Leukemia | 89.22 | 9.61 | 3.67 |
|  | A432 | Acute Myeloid Leukemia | 87.25 | 9.40 |  |
|  | G691 | Acute Myeloid Leukemia | 33.33 | 3.59 |  |
|  | L769 | Acute Myeloid Leukemia | 87.25 | 9.40 |  |
|  | S836 | Acute Myeloid Leukemia | 10.78 | 1.16 |  |
|  | S904 | Acute Myeloid Leukemia | 33.33 | 3.59 |  |
| *RHOA* | Y42 | Stomach | 26.32 | 1.47 | 0.15 |
| *SMAD4* | R361 | Large Intestine | 24.28 | 2.15 | 0.36 |
|  | R361 | Pancreas | 10.34 | 1.41 |  |
| *SOCS1* | A3 | Diffuse Large B Cell Lymphoma | 10.00 | 1.72 | 0.88 |
|  | A17 | Diffuse Large B Cell Lymphoma | 10.00 | 1.72 |  |
|  | Y64 | Diffuse Large B Cell Lymphoma | 7.50 | 1.29 |  |
|  | F79 | Diffuse Large B Cell Lymphoma | 7.50 | 1.29 |  |
|  | C111 | Diffuse Large B Cell Lymphoma | 6.25 | 1.08 |  |
|  | S116 | Diffuse Large B Cell Lymphoma | 10.00 | 1.72 |  |
| *SPOP* | F102 | Prostate | 18.91 | 1.34 | 0.47 |
|  | F133 | Prostate | 47.76 | 3.39 |  |
| *SRSF2* | D48 | Acute Myeloid Leukemia | 38.52 | 2.39 | 0.80 |
|  | P95 | Acute Myeloid Leukemia | 51.23 | 3.18 |  |
|  | S134 | Acute Myeloid Leukemia | 39.75 | 2.46 |  |
| *STAT6* | D419 | Diffuse Large B Cell Lymphoma | 62.50 | 6.49 | 0.65 |
| *TGFBR2* | R528 | Pancreas | 43.28 | 1.94 | 0.19 |
| *TP53* | P72 | Acute Myeloid Leukemia | 34.00 | 3.01 | 12.86 |
|  | R158 | Lung | 3.72 | 1.93 |  |
|  | R175 | Breast | 4.41 | 2.20 |  |
|  | R175 | Diffuse Large B Cell Lymphoma | 5.73 | 1.75 |  |
|  | R175 | Esophagus | 5.90 | 4.54 |  |
|  | R175 | Large Intestine | 11.79 | 7.13 |  |
|  | R175 | Pancreas | 6.17 | 2.99 |  |
|  | R175 | Stomach | 8.59 | 4.37 |  |
|  | C176 | Esophagus | 2.14 | 1.65 |  |
|  | H179 | Diffuse Large B Cell Lymphoma | 4.69 | 1.43 |  |
|  | H179 | Esophagus | 1.88 | 1.45 |  |
|  | H193 | Esophagus | 1.57 | 1.21 |  |
|  | R196 | Large Intestine | 2.17 | 1.31 |  |
|  | R196 | Stomach | 2.17 | 1.10 |  |
|  | R213 | Breast | 2.51 | 1.25 |  |
|  | R213 | Esophagus | 2.66 | 2.05 |  |
|  | R213 | Large Intestine | 3.32 | 2.01 |  |
|  | R213 | Pancreas | 3.54 | 1.71 |  |
|  | R213 | Stomach | 2.86 | 1.46 |  |
|  | Y220 | Breast | 2.10 | 1.05 |  |
|  | Y220 | Esophagus | 1.93 | 1.49 |  |
|  | Y220 | Pancreas | 2.17 | 1.05 |  |
|  | G245 | Breast | 2.04 | 1.01 |  |
|  | G245 | Diffuse Large B Cell Lymphoma | 5.21 | 1.59 |  |
|  | G245 | Esophagus | 3.92 | 3.01 |  |
|  | G245 | Large Intestine | 4.29 | 2.60 |  |
|  | G245 | Stomach | 5.03 | 2.56 |  |
|  | R248 | Breast | 6.75 | 3.36 |  |
|  | R248 | Diffuse Large B Cell Lymphoma | 11.46 | 3.50 |  |
|  | R248 | Esophagus | 6.01 | 4.62 |  |
|  | R248 | Large Intestine | 10.41 | 6.30 |  |
|  | R248 | Lung | 3.98 | 2.07 |  |
|  | R248 | Pancreas | 6.17 | 2.99 |  |
|  | R248 | Prostate | 7.24 | 1.49 |  |
|  | R248 | Stomach | 7.99 | 4.07 |  |
|  | R249 | Liver | 28.11 | 11.97 |  |
|  | R273 | Breast | 5.53 | 2.75 |  |
|  | R273 | Diffuse Large B Cell Lymphoma | 4.69 | 1.43 |  |
|  | R273 | Esophagus | 4.02 | 3.09 |  |
|  | R273 | Large Intestine | 7.61 | 4.61 |  |
|  | R273 | Lung | 5.90 | 3.07 |  |
|  | R273 | Pancreas | 7.20 | 3.48 |  |
|  | R273 | Prostate | 7.24 | 1.49 |  |
|  | R273 | Stomach | 6.86 | 3.49 |  |
|  | P278 | Esophagus | 1.46 | 1.13 |  |
|  | R282 | Esophagus | 4.44 | 3.42 |  |
|  | R282 | Large Intestine | 5.75 | 3.48 |  |
|  | R282 | Pancreas | 4.46 | 2.16 |  |
|  | R282 | Stomach | 3.47 | 1.77 |  |
|  | R342 | Esophagus | 1.46 | 1.13 |  |
| *ZNF521* | R991 | Pancreas | 68.29 | 3.01 | 0.30 |

^1^ Leukemia and lymphoma are among the 10 deadliest cancers. Data summarized relative to these cancer types are acute myeloid leukemia and diffuse large B cell lymphoma, respectively. These were chosen because are major contributors to leukemia and lymphoma deaths and are well-represented in the COSMIC database.

^2^ Percentages of individual genes mutated in total cancers (for different cancer types) were added then divided by 10, to summarize mutated gene representation across the ten deadliest cancers.

**Supporting Information Figure 1. Percentages of amino acid codons that are targets for hotspots mutation varies by cancer type.**


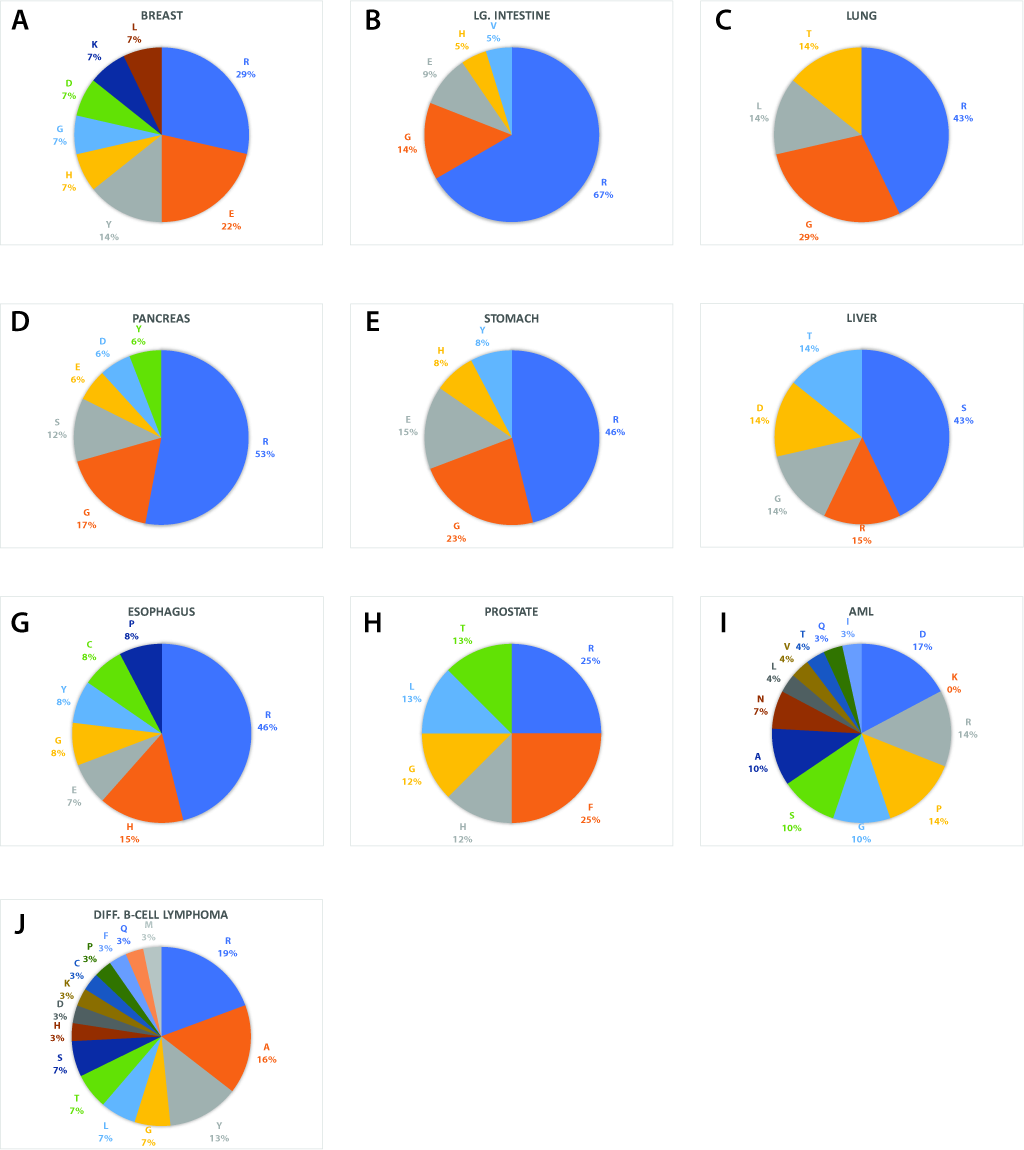


**Supporting Information Figure 2. Venn diagrams indicate the cell communication function(s) ascribed to hotspot CD gene products (A) and COSMIC Tier 1 and Tier 2 gene products (B).** The percentages correspond to the number of genes related to each type of cell communication (or combination of cell communication types) relative to the total number of genes analyzed (with and without functions in cell communication, 40 and 348 gene products, respectively).


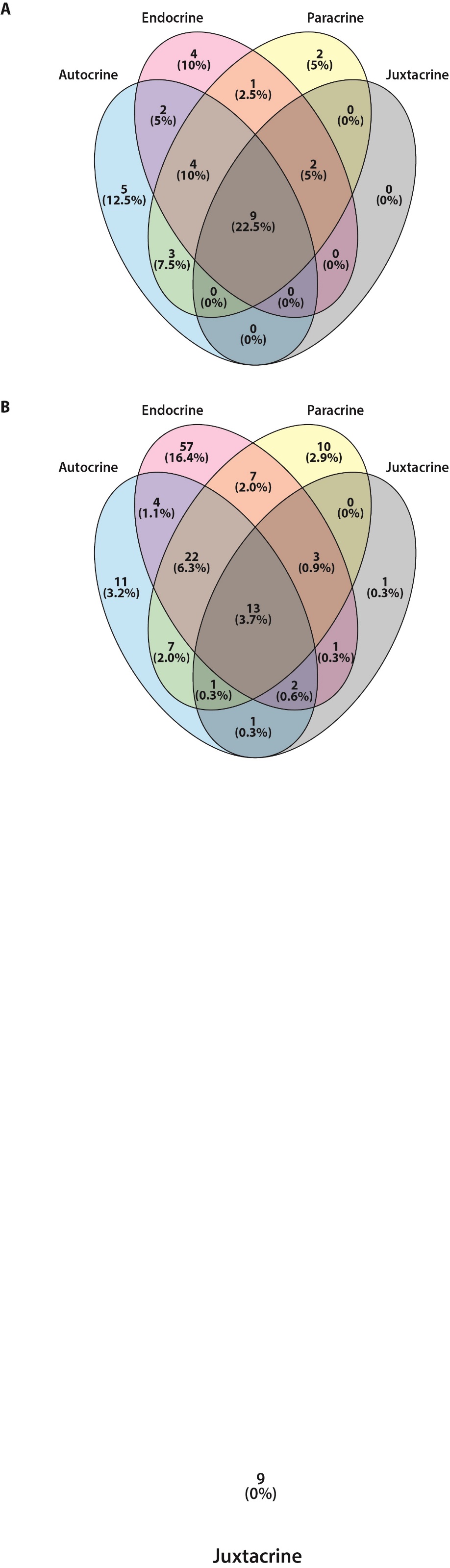

Supplement: Supplementary file 1 — Appendix S1: Supporting Information [file EM-61-152-s001.docx]
